# Single-cell RNA sequencing identifies CXADR as a fate determinant of the placental exchange surface

**DOI:** 10.1038/s41467-024-55597-w

**Published:** 2025-01-02

**Authors:** Dafina M. Angelova, Aleksandra Tsolova, Malwina Prater, Noura Ballasy, Wendi Bacon, Russell S. Hamilton, Danielle Blackwell, Ziyi Yu, Xin Li, Xin Liu, Myriam Hemberger, D. Stephen Charnock-Jones

**Affiliations:** 1 Department of Obstetrics and Gynaecology, University of Cambridge, NIHR Cambridge Biomedical Research Centre, Cambridge, United Kingdom; 2 Loke Centre for Trophoblast Research, Department of Physiology, Development, and Neuroscience, University of Cambridge, Cambridge, United Kingdom; 3 Department of Biochemistry and Molecular Biology, Cumming School of Medicine, University of Calgary, 3330 Hospital Drive NW, Calgary, Alberta Canada; 4 Alberta Children’s Hospital Research Institute, University of Calgary, 3330 Hospital Drive NW, Calgary, Alberta Canada; 5 Functional Genomics Centre, Cancer Research Horizons, Milner Therapeutics Institute, Jeffrey Cheah Biomedical Centre, University of Cambridge, Cambridge, United Kingdom; 6 School of Life, Health & Chemical Sciences, The Open University, Milton Keynes, United Kingdom; 7 College of Chemical Engineering, Nanjing Tech University, Nanjing, People’s Republic of China; 8 Sphere Fluidics Ltd., Building One, Granta Centre, Granta Park, Great Abington, Cambridge, England United Kingdom

**Keywords:** Differentiation, Stem-cell differentiation, Stem-cell differentiation

## Abstract

The placenta is the critical interface between mother and fetus, and consequently, placental dysfunction underlies many pregnancy complications. Placental formation requires an adequate expansion of trophoblast stem and progenitor cells followed by finely tuned lineage specification events. Here, using single-cell RNA sequencing of mouse trophoblast stem cells during the earliest phases of differentiation, we identify gatekeepers of the stem cell state, notably *Nicol1*, and uncover unsuspected trajectories of cell lineage diversification as well as regulators of lineage entry points. We show that junctional zone precursors and precursors of one of the two syncytial layers of the mouse placental labyrinth, the Syncytiotrophoblast-I lineage, initially share similar trajectories. Importantly, our functional analysis of one such lineage precursor marker, CXADR, demonstrates that this cell surface protein regulates the differentiation dynamics between the two syncytial layers of the mouse labyrinth, ensuring the correct establishment of the placental exchange surface. Deciphering the mechanisms underlying trophoblast lineage specification will inform our understanding of human pregnancy in health and disease.

## Abstract (web summary)

Placental formation requires an adequate expansion of trophoblast stem and progenitor cells followed by finely tuned lineage specification events. Here, using single-cell analysis of mouse trophoblast stem cells the authors identify *Nicol1* as a gatekeeper of the stem cell state and show that CXADR regulates the differentiation dynamics between the two syncytial layers of the mouse placenta.

## Introduction

The placenta provides the critical interface between the maternal and fetal bloodstreams and, as such, is the site of exchange of nutrients, oxygen, metabolites and other molecules. The great obstetric syndromes (e.g., preeclampsia, fetal growth restriction, preterm birth) are linked to disorders of placentation^1^. Low birthweight and preeclampsia are associated with increased perinatal mortality and long-term morbidities such as cardiovascular disease risk^2,3^. These impose substantial health and socioeconomic burdens^4^. Thus, understanding the mechanisms underlying placental dysfunction is clinically and economically important. Despite this importance, the molecular pathways involved in placenta formation remain comparatively poorly studied and incompletely understood.

The extra-embryonic trophoblast lineage gives rise to the major constituents of the placenta. The trophoblast lineage first emerges as the outer layer of the blastocyst. During subsequent development, proliferative trophoblast cells with stem-like potential differentiate into multiple lineages with differing locations, characteristics and functions^5–7^. The layer of trophoblast in direct contact with maternal blood is a syncytium (a single layer in human and 2 layers in the mouse). This syncytiotrophoblast constitutes the interface for all nutrient and gas exchange between mother and fetus and, thus, is critical for fetal growth and survival. Yet the characterisation of the molecular mechanisms that underlie trophoblast lineage specification and further differentiation into mature trophoblast cell types, such as syncytiotrophoblast, remains a key challenge.

Trophoblast stem cells (TSCs) are a self-renewing stem cell population representative of the trophoblast lineage^8^. Their derivation from early mouse blastocysts was achieved 26 years ago and, more recently, was also successful from the human placenta^8–11^. The ability to maintain TSCs as a self-renewing stem cell population in culture represented a milestone in developmental biology. TSCs endow us with unprecedented possibilities to explore molecular, biochemical, genetic and environmental aspects that direct trophoblast proliferation and differentiation in vitro. These advances have collectively spurred our understanding of the developmental principles governing placentation^12–15^.

Maintenance of the stem cell state of murine TSCs is usually achieved through the addition of fibroblast growth factor (FGF) and mouse embryonic fibroblast conditioned medium (CM) to the culture medium. CM provides Activin and/or TGFβ components that contribute to the maintenance of the self-renewal capacity of TSCs^16,17^. Removal of both FGF and CM induces TSCs to differentiate along the two major lineage trajectories towards junctional zone and trophoblast giant cells on the one hand, and towards syncytiotrophoblast cells (SynT) of the placental labyrinth on the other. However, under these standard differentiation conditions, TSCs preferentially form trophoblast giant cells, which typify the junctional zone route over a time course of ≥6 days.

Comparing the relative importance of the FGF and CM-induced signalling cascades for TSC self-renewal has demonstrated that FGF is the dominant pathway^18,19^. Chemical inhibition of MEK, the major kinase downstream of the FGF receptor and a key signalling hub of the MAPK cascade, leads to the abrupt exit from self-renewal and onset of differentiation within a few hours^18,19^. This is best demonstrated by the acute down-regulation of genes that are direct targets of MAPK signalling such as *Esrrb* and *Sox2*^18,19^. By contrast, the CM-mediated effects are more subtle and continue to direct TSC fate even after exit from the self-renewal state. Continued exposure to Activin in the absence of FGF prolongs the expression of SynT lineage markers specifically^20^. Thus, TGFβ/Activin have nuanced roles in both TSC self-renewal and in directing differentiation that depend on the concomitant presence or absence of FGF.

It has long been recognized that TSCs appear morphologically heterogenous even when grown in stem cell conditions. On the molecular level this is underpinned by notable differences in the levels of key stem cell transcription factors such as ESRRB, SOX2, CDX2, EOMES, and TFAP2C between individual cells^18,21,22^. Single cell cloning approaches have tied this heterogeneity to heritable morphological differences of emerging colonies that relate to the degree of epithelial characteristics^22^. However, whether these differences reflect first biases in cellular potency that would skew the differentiation potential of any given cell in favour of either junctional zone or SynT lineage entry has remained undetermined. This knowledge gap pertains in particular to the fact that molecular drivers of early TSC differentiation, encompassing the phase when the critical lineage bifurcation events occur between junctional zone precursors (JZP) and labyrinth precursors (LP), have remained poorly defined and only rarely functionally explored.

Here, we used a single-cell RNA sequencing (scRNA-seq) approach coupled to an experimental design that was specifically aimed at identifying molecular drivers of early trophoblast lineage differentiation. To this end, we employed the traditional TSC differentiation method by withdrawal of FGF and CM, as well as MEK inhibition in the continued presence of FGF and CM, to tease out candidate factors governing lineage entry towards the JZP and LP differentiation trajectory, respectively. Our data reveal previously unknown regulators of the stem cell state, notably NICOL1, as well as of specific differentiation trajectories. Our analysis culminates in the identification of CXADR as a factor of critical importance for regulating the cell fusion dynamics in the differentiation of the two SynT layers, thereby ensuring the correct establishment of the placental exchange surface.

## Results

### Resolving early lineage priming at single-cell level by Drop-seq

**Fig. 1:**
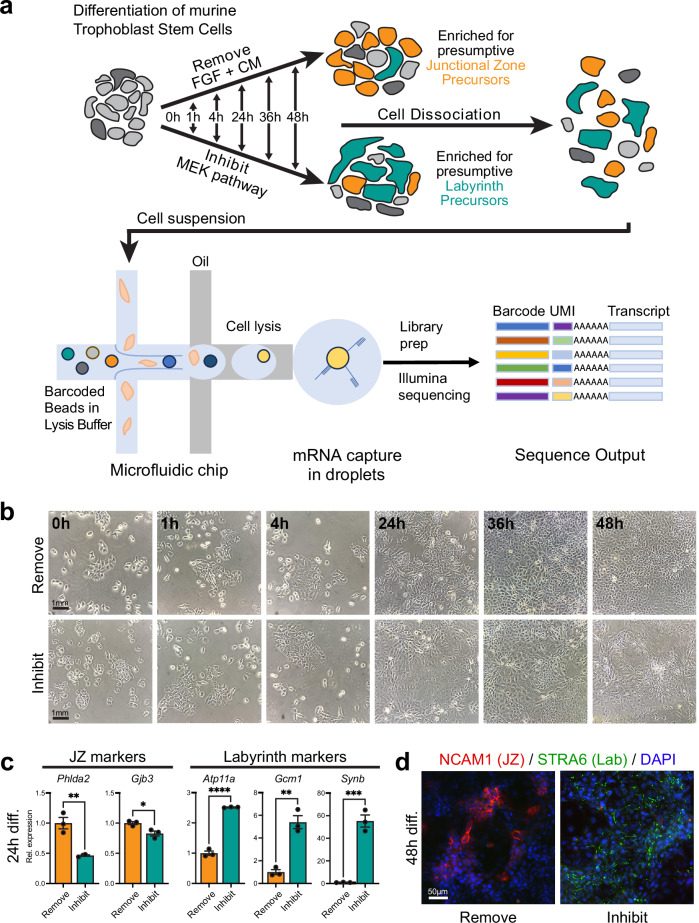
Description of the mouse TSC differentiation time course experiment. **a** In vitro differentiation of mouse TSCs was induced by withdrawal of the stemness-maintaining components FGF and embryonic fibroblast conditioned medium (CM; Remove dataset) or by inhibition of MEK with PD0325901 (Inhibit dataset). Remove conditions are expected to favour initial lineage entry towards junctional zone precursors, whereas the continued presence of CM in Inhibit conditions is expected to enrich for labyrinth precursors. Samples were collected at t0 (stem cell state, *n* = 2 independent experiments), 1 h (*n* = 4 independent experiments Inhibit and Remove), 4 h (*n* = 4 independent experiments Inhibit and *n* = 3 independent experiments Remove), 24 h (n = 4 independent experiments Inhibit and *n* = 2 Remove), 36 h (*n* = 4 independent experiments Inhibit and Remove) and 48 h (*n* = 4 independent experiments Inhibit and Remove). Cells were dissociated and single cell barcoded transcriptome libraries were generated using Drop-seq and sequenced. UMI = Unique molecular identifier. **b** Phase contrast images of cells cultured over the outlined time course in Remove and Inhibit conditions. Images are representative of three independent experiments. **c** RT-qPCR for junctional zone (JZ) and labyrinth markers after 24 h of differentiation in Remove or Inhibit conditions. Data are normalised to Remove conditions at 24 h and plotted as mean +/- SEM of *n* = 3 independent replicates. Statistical significance was calculated using two-sided unpaired t-test. * *p* < 0.05, ** *P* < 0.01, *** *p* < 0.001, **** *p* < 0.0001. Source data are provided as a Source Data file which contains exact P values. Rel. expression = relative expression. **d** Immunofluorescence staining of cells grown for 48 h in Remove and Inhibit conditions for JZ marker NCAM1 (red) and labyrinth (Lab) marker STRA6 (green). Image is representative of three independent experiments.

We generated transcriptome libraries from 136,291 TSCs in the stem cell state and at various stages of differentiation. Differentiation was induced either by MEK inhibition (hereafter, Inhibit) or in the conventional way by withdrawal of FGF and CM (Remove). This experimental strategy was designed to reveal genes that are regulated primarily by FGF versus TGFβ/Activin, the latter being contained in the CM. Specifically, the differentiation bias introduced by these methods towards the LP (Inhibit) versus JZP (Remove) lineage was aimed at identifying markers and drivers of lineage entry and early lineage priming. Using the stem cell state (0 h = t0) as the starting point, we profiled cells after 1 h (t1), 4 h (t4), 24 h (t24), 36 h (t36) and 48 h (t48) of differentiation induced by the Remove or Inhibit conditions (Fig. 1a, Supplementary Data 1 for replicates, sequencing and analysis batches). We obtained an average of 1740 reads and 1,059 genes per cell.

Morphological inspection of culture dishes over this time course period showed that cells differentiated more rapidly in Inhibit conditions, as expected^18,19^, with no obvious impact on cell viability (Fig. 1b, Supplementary Fig. 1). This was evident by patches of cells with a more flattened appearance and less obvious cell boundaries, and reduced cell growth overall (Fig. 1b). We also verified that the two differentiation strategies achieved a lineage bias in favour of junctional zone and labyrinth differentiation in Remove and Inhibit conditions, respectively (Fig. 1c, d). Thus, after 24 h, junctional zone markers *Phlda2* and *Gjb3* were enriched in Remove conditions, whereas SynT markers *Atp11a*, *Gcm1* and *Synb* were more highly expressed in Inhibit conditions (Fig. 1c). Similarly, using cell type-specific markers previously identified in placental scRNA-seq approaches^6^ for immunostaining of cells after 48 h, Remove cells stained more pervasively for junctional zone-expressed NCAM1, whereas Inhibit cells were enriched for labyrinth marker STRA6 (Fig. 1d).

### Heterogeneity of TSCs in stem cell conditions

**Fig. 2:**
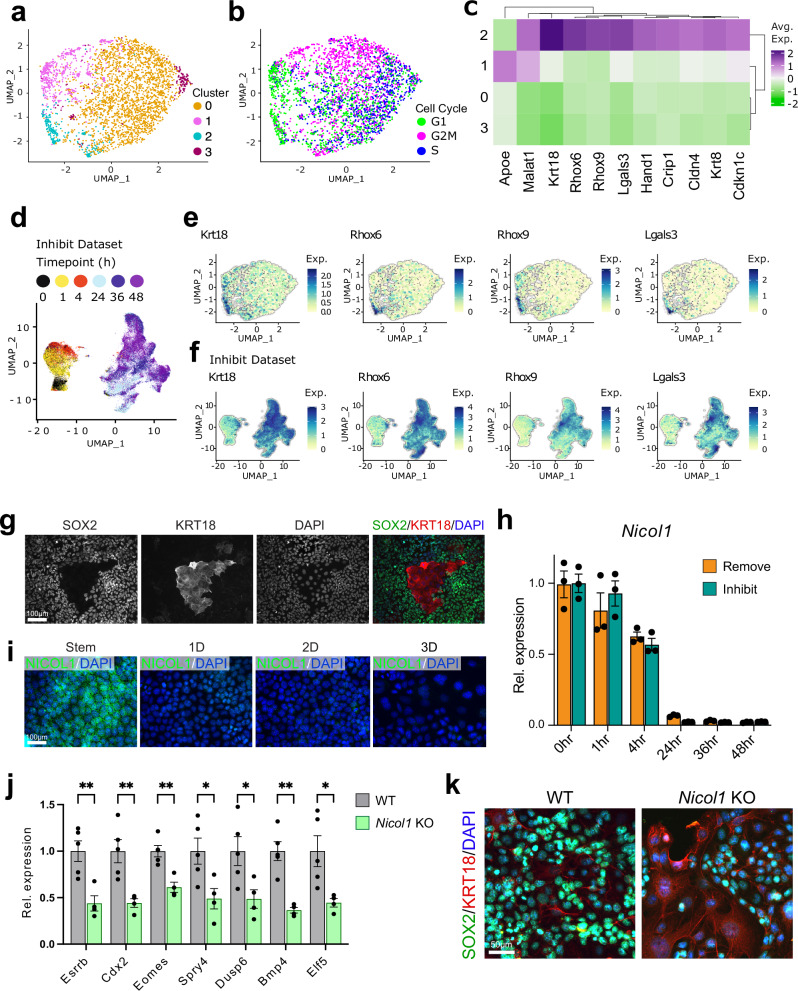
Heterogeneity of TSCs. **a-b, d** UMAP grid for Harmony-integrated t0 dataset. Dimensional reduction of the dataset using UMAP (uniform manifold approximation and projection, calculated in Seurat) after harmony integration. **a** UMAP coloured by Clusters, resolution 0.4 (b) UMAP coloured by assigned Cell Cycle Phase using the Seurat function CellCycleScoring. **c** Heatmap of expression of t0 cluster markers. All values were mean centred for each gene. Colour represents mean centered average expression of marker gene in a cluster. **d** UMAP plot of Inhibit dataset with cells coloured by timepoint. **e** UMAP plots highlighting the top 4 cluster 2-enriched genes. **f** Elevated genes from Cluster 2 of t0 cells are up-regulated during further differentiation: UMAP plots of *Krt18*, *Rhox6*, *Rhox9* and *Lgals3* in entire Inhibit dataset. Exp. = Normalised expression. **g** Double immunofluorescence staining for TSC transcription factors SOX2 (green) and early differentiation marker KRT18 (red) of TSCs grown in stem cell conditions. Image is representative of three independent experiments. **h** RT-qPCR for *Nicol1* in TSCs in the stem cell state and upon differentiation in Remove and Inhibit conditions for the indicated time periods. Data are normalised to stem cell conditions and plotted as mean +/- SEM of three independent replicates. **i** Immunofluorescence staining for NICOL1 (green) in TSCs grown in stem cell conditions and upon differentiation in Remove for 1, 2, and 3 days (D). Images are representative of three independent experiments. **j** Assessment of stem cell potential of *Nicol1* knockout (KO) TSCs showing reduced levels of acutely sensitive marker genes of the stem cell state in the trophoblast compartment. Data are normalized to wild-type (WT) TSCs in stem cell conditions and are shown as mean +/- SEM of *n* = 5 (WT) and *n* = 4 (KO) independently derived cell clones. Statistical significance was calculated using two-sided unpaired t-test. * *p* < 0.05, *** *p* < 0.001, **** *p* < 0.0001. Source data are provided as a Source Data file which contains exact P values. **k** Double immunofluorescence staining for stem cell marker SOX2 (green) and early differentiation marker KRT18 (red) on WT and *Nicol1* KO TSCs grown in stem cell conditions. Images are representative of *n* = 3 clones stained each.

As a starting point to our analysis, we aimed at determining the heterogeneity of TSCs in the stem cell state. To this end, we first investigated the sub-populations of cells derived from the t0 ( = TSC conditions) time point separately. The t0 dataset contained 3,432 cells with a mean of 1,632 reads and 996 genes per cell. We visualised the cells on a UMAP plot at resolution 0.4, which identified 4 dispersed clusters (Fig. 2a, Supplementary Fig. 2a, Supplementary Data 2). We also scored each stem cell for cell cycle stage and found cells in all three stages of the cell cycle, i.e. S, G1 and G2/M, indicating that the t0 cells were highly proliferative, as expected (Fig. 2b).

We identified differentially expressed genes (DEGs) for each t0 cluster with *P*_adj_ < 0.05 and absolute fold-change ≥1.5 using the Wilcoxon rank sum test and determined the average expression for the detected marker genes above the threshold for each cluster (Supplementary Data 3). Cluster 2 was distinct from the other clusters with marker genes linked to differentiation such as *Krt18*, *Rhox6*, *Rhox9*, and *Lgals3* (Fig. 2c, e), suggesting that this cluster may consist of cells primed for differentiation^23,24^. This was further investigated by assessing the expression levels of these genes at later time points of differentiation, specifically in t48 cells of the Inhibit data set (Fig. 2d-f), which confirmed their further upregulation on differentiation. We also performed immunostaining for the stem cell marker SOX2 in combination with cluster 2 factor KRT18 on TSCs and observed that KRT18-positive cells had a flattened, enlarged appearance indicative of differentiation onset and had strikingly lost SOX2 as an acutely sensitive TSC marker (Fig. 2g). When we plotted the cluster 0 cells on the full-time course UMAP we found that only 3% of them cluster together with t24-t48 cells compared to 30% of cluster 2 cells (Supplementary Fig. 2b-e). These data show that TSCs contain a small subset of cells that are already primed for differentiation even in stem cell culture conditions, akin to what is observed in embryonic stem cells and other stem cell types^25–27^.

To investigate classical TSC markers in the t0 dataset we plotted the expression of *Elf5*, *Esrrb*, *Cdx2*, *Eomes*, *Tead4*, *Sox2* in each cluster and found that clusters 0 and 3 had the highest expression levels, whereas expression of these stem cell markers was lowest in cluster 2 (Supplementary Fig. 2f). To identify other markers which might distinguish between cells in the clusters with highest and lowest expression of stem cell markers, we calculated pairwise differentially expressed genes between the t0 cluster 0 and cluster 2 (Supplementary Data 4). We found that the top 6 genes elevated in cluster 0 (Supplementary Fig. 2g, h) were more highly expressed than classical TSC markers; of these, *Gm1673* (a.k.a. *Nicol1*) appeared most specific for clusters 0 and 3. *Nicol1* encodes a recently identified small secreted protein highly expressed in male and female reproductive tissues^28^. To explore a potential functional role of *Nicol1* in TSCs, we first confirmed its acute down-regulation upon onset of differentiation on the mRNA and protein level (Fig. 2h, i). These data establish *Nicol1* as a strictly stem cell-associated gene in the trophoblast compartment that may be important to maintain the stem cell state. We further determined such a potential role by generating knockout (KO) TSCs for *Nicol1* (Supplementary Fig. 3a-c) and assessed the impact of *Nicol1* deficiency on expression levels of various stem cell markers. Indeed, we found that *Nicol1* ablation causes a significant down-regulation of stem cell marker expression levels, implying that *Nicol1* is essential to maintain stemness in TSCs (Fig. 2j, k, Supplementary Fig. 3d). This proof-of-concept example underscores the value of our data in the identification of critical TSC factors.

### Differentiation events induced by MEK inhibition and FGF/CM removal

Next, we integrated all time points of the scRNA-seq data while assessing the Inhibit and Remove data sets separately. For simplicity and because the MEK inhibition exerts a stronger effect on early TSC differentiation, we show analyses using the Inhibit dataset in the main text with corresponding Remove plots presented in the Supplementary Data (Supplementary Figs. 4, 8, 13).

**Fig. 3:**
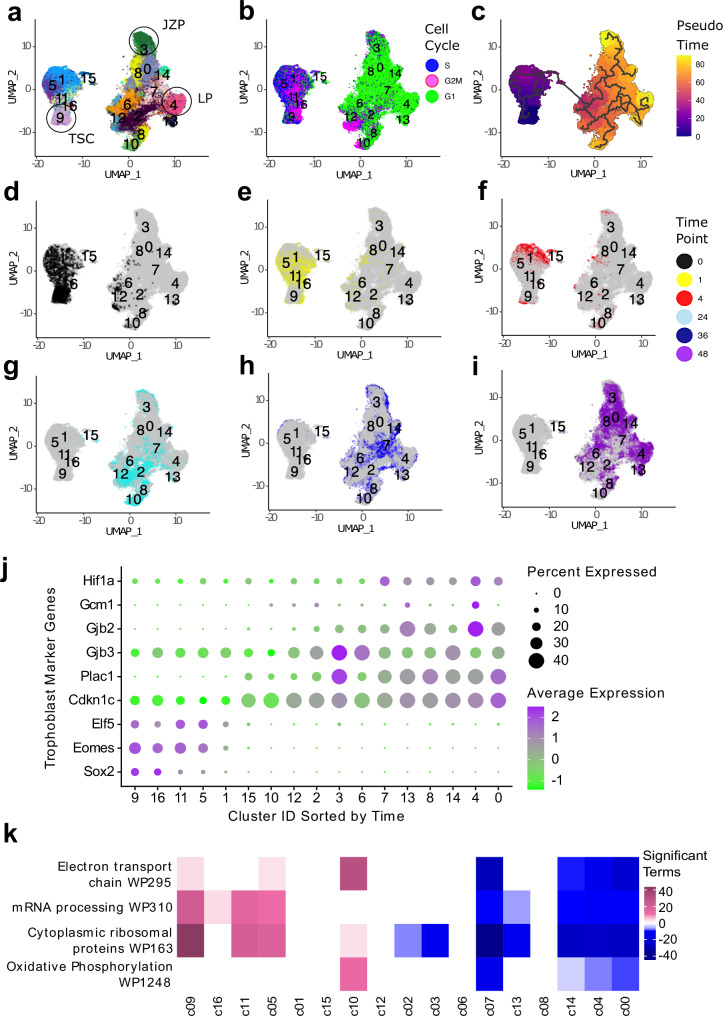
Clustering of Inhibit dataset. **a-i** UMAP grid of the Harmony-integrated Inhibit TSC differentiation dataset. **a** UMAP coloured by cluster, resolution = 0.8. TSC = Trophoblast stem cells, JZP = Junctional zone progenitors, LP = Labyrinth progenitors. **b** UMAP coloured by assigned cell cycle phase using the Seurat function CellCycleScoring. **c** UMAP coloured by Pseudotime using Monocle3. **d–i** UMAPs with cells coloured at timepoints: 0 h (**d**) 1 h (**e**) 4 h (**f**) 24 h (**g**) 36 h (**h**) and 48 h (**i**). **j** Dotplot of marker genes expressed in the Inhibit dataset. Markers were selected from literature and grouped by differentiation trajectory. (TSC markers: *Eomes*, *Elf5*, *Sox2*. JZP markers: *Cdkn1c*, *Gjb3*, *Plac1*. LP markers: *Gjb2*, *Gcm1*, *Hif1a*.) The size of the dots represents number of cells that express the marker. The colour of dots represents the average expression of the marker in each cluster, scaled for the dataset. All values were mean centred for each gene. Clusters were ordered on the x-axis by time using the R package Tempora with undifferentiated cells to the left of the x-axis. **k** Heatmap of gene expression in top WikiPathways in the Inhibit dataset. Pink and blue represent enrichment or under-representation of a pathway in each specific cluster, respectively. Clusters on the x-axis are ordered by Tempora with stem cells on the left.

The Inhibit dataset separated into 17 clusters which self-segregated into two large groups split by time point (t0-t4 and t24-t48, Fig. 3a, Supplementary Data 5 and 6). There was good agreement between the top 50 most variable genes in the Inhibit scRNA-seq data and the similarly treated bulk RNA-seq data set (Supplementary Fig. 5a).

As TSCs differentiate, the fraction of cells in the S and G2/M phases of the cell cycle diminishes^8^. We performed cell cycle scoring in Seurat to observe the temporal changes in the differentiating TSC. We expected these changes to happen more rapidly in cells in which FGF signalling was rapidly blocked (i.e. the Inhibit cells) than in Remove conditions. Examination of the UMAP plots corroborated this expectation. Cells from early time points (t0-t4) (Fig. 2d) were predominantly in S or G2/M phase, whereas cells at t24 and later time points were overwhelmingly in G1 phase (Fig. 3b). Moreover, cells underwent this transition quicker in Inhibit conditions than in Remove conditions (Supplementary Fig. 4, 5b), in line with expectations. To identify distinct differentiation trajectories as cell lineages emerge in differentiating TSCs, we performed Pseudotime trajectory analysis with Monocle 3^29^. Monocle traced paths from the t0 cells in cluster 9 (c9) to t36-t48 cells in c3 and c4, suggesting that the cells in these later clusters were the furthest along in their differentiation trajectory (Fig. 3c-i).

### Temporal progression of differentiation into lineage precursors

Our next approach was to infer the order of cell clusters using the Tempora R package^30^. This package makes explicit use of the known temporal relationships in the data. To validate the gene expression patterns over time, we compared our Inhibit data to bulk RNA-seq time-course data of TSCs treated with MEK inhibitor^18^ We identified the 50 most variable genes from the single cell dataset and plotted their average expression score in the bulk and the single cell Inhibit dataset (Supplementary Fig. 5a). The single cell data showed a strong similarity to the bulk data with a pronounced shift in transcript profiles at t24. These observations support the Tempora ordering of single cell clusters over the time course. To confirm the identities and further evaluate the temporal ordering of our cell clusters, we selected three markers each for TSCs in the stem cell state, the JZP differentiation trajectory and for SynT-directed LP lineages and plotted their expression in each cluster^31^. As expected, the expression for stem cell markers *Eomes*, *Elf5* and *Sox2* decreased over the course of differentiation while *Cdkn1c*, *Plac1* and *Gjb3* (for JZP) and *Gcm1*, *Gjb2* and *Hif1a* (for LP) increased in a cluster-specific pattern (Fig. 3j). In the Remove dataset, trophoblast differentiation markers showed similar temporal trends, but these were slightly delayed with TSC markers persisting longer into the time course (Supplementary Fig. 4j). Thus, the ordering of clusters is in good agreement with what is observed in bulk RNA analysis.

We identified the marker genes that characterised each cluster with the Seurat function Findallmarkers using a Wilcoxon rank sum test with P_adj_ < 0.05 and 1.2 times fold-change cut-offs (Supplementary Fig. 6, Supplementary Data 7 and 8). Marker genes for clusters containing cells from early time points were related to stem cell maintenance, ribosomal proteins and mitochondrial enzymes, while at later time points, markers were related to trophoblast differentiation such as *Cdkn1c*, *Plac1*, keratins and galectins. We also identified genes that were differentially expressed between all cluster pairs. We used these marker genes to perform pathway enrichment analysis using EnrichR with the WikiPathways and GO databases (Supplementary Figs. 7, 8, 9, Supplementary Data 9-20). The most highly enriched terms were related to transcription, translation, ribosomal biogenesis, mitochondrial respiration, regulation of pluripotency and cytoskeletal remodelling. These were highly enriched at early time points and decreased over time (Fig. 3k; Supplementary Fig. 4k). No significant enrichment scores were identified for genes enriched at later time points. Overall, this data analysis confirmed that our cluster calling and pseudotime trajectory analysis represented a correct reflection of the differentiation processes occurring during TSC lineage specification.

### Identification of transcription factor networks enriched during trophoblast differentiation

TSC differentiation can be characterised by the activity of transcription factors (TFs) whose target genes change in expression levels as trophoblast lineages are specified. To identify such transcription factor modules governing TSC self-renewal, as well as JZP and LP lineage entry, we used SCENIC^32,33^. We calculated transcription factor regulon activity (ie modules of co-expressed genes and transcription factors with binding motif support) in the dataset (Supplementary Fig. 10a, b).

**Fig. 4:**
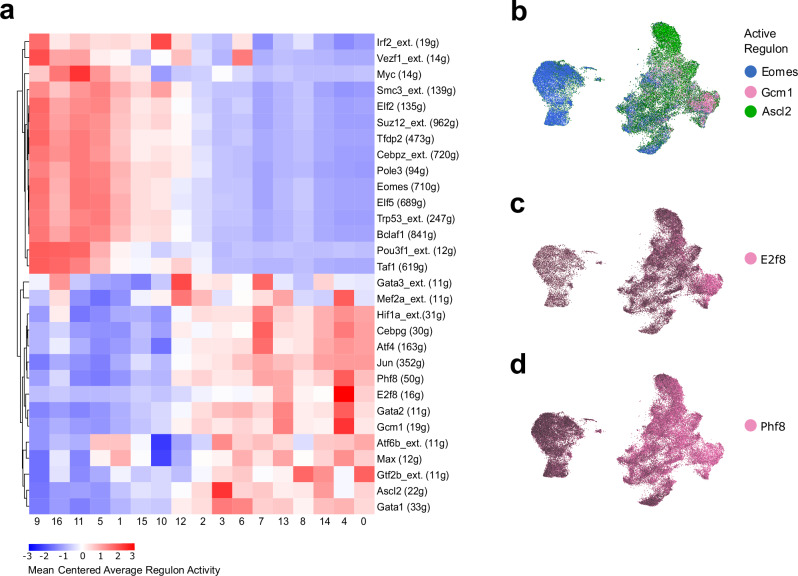
Active regulons in the Inhibit dataset. **a** Heatmap of regulon activity that co-occurs with known TF markers for LP and JZP identities. JZP identity was assigned by detecting clusters with high activity of the *Ascl2* regulon (c3), and LP identity by the *Gcm1* regulon (c4). Difference between JZP cluster activity and LP cluster activity was calculated for each regulon. Top 10 regulons for highest and lowest ratio, as well as top 10 regulons with highest activity in t0 cluster (cluster 9) were selected for the heatmap. Colour represents average score for the activity of a regulon for each cluster. All values were mean centred for each regulon and number of genes in the regulon is shown in brackets. The suffix _ext. in the regulon name indicates lower confidence annotations (by default inferred by motif similarity) are also used. **b-d** Specific SCENIC transcription factor regulons co-localize with TSCs, and cells associated with JZP and LP lineage. Cells were coloured by AUC score for regulons of interest. (b) UMAP plot of Inhibit dataset coloured by regulon activity. The TSCs-specific regulons *Eomes* are coloured blue, JZP regulon *Ascl2* is coloured green, and LP regulon *Gcm1* is in pink. **c** UMAP plot of Inhibit dataset coloured by *E2f8* regulon activity. *E2f8* regulon activity overlaps *Gcm1* activity. **d** UMAP plot of Inhibit dataset coloured by *Phf8* regulon activity. *Phf8* regulon activity is increased over time in LP cells and overlaps with *E2f8* and *Gcm1* activity.

To identify the most active regulons in TSCs, JZP cells and LP cells, we calculated ratios between the regulon activity scores of clusters (TSC cluster = c9, JZP cluster = c3, LP cluster = c4) and selected the highest and lowest 10 values (Fig. 4a). The TSC cluster was enriched for *Eomes*- and *Elf5*-directed regulons – TFs known to be enriched in TSCs^34–36^. *Ascl2* was enriched in JZP and *Gcm1* in LP, confirming known TF circuits and corroborating our marker-based cluster identification.

Some active regulons specific to the TSC clusters (*Eomes*, *Suz12*, *Myc*, *Smc3*, *Elf2*, *Tfdp2*, *Pole3*, *Trp53, Taf1, Irf2, Pou3f1*), have been previously reported to act in trophoblast^37–42^. TSC-specific regulons such as *Vezf1*, *Bclaf1* and *Cebpz* have not been linked to the maintenance of mouse trophoblast stemness, suggesting that these may be regulators revealed by our analysis. *Vezf1*, encoding for a vascular endothelial zinc finger, is highly expressed in the mature placental labyrinth^43^ but in vitro is enriched in TSCs compared to differentiated trophoblast cell types^21,44^. This supports the notion that TSCs are able to acquire pseudo-endothelial characteristics^45^.

We only identified a single regulon (*Ascl2*) specific to the JZP cells in the Inhibit dataset. In contrast, we identified several regulons specifically associated with the LP lineage: *E2f8*, *Phf8*, *Gata2* and *Mef2a* (Fig. 4b-d, Supplementary Fig. 11). *E2f8* was the most active regulon in the LP cluster c4.

**Fig. 5:**
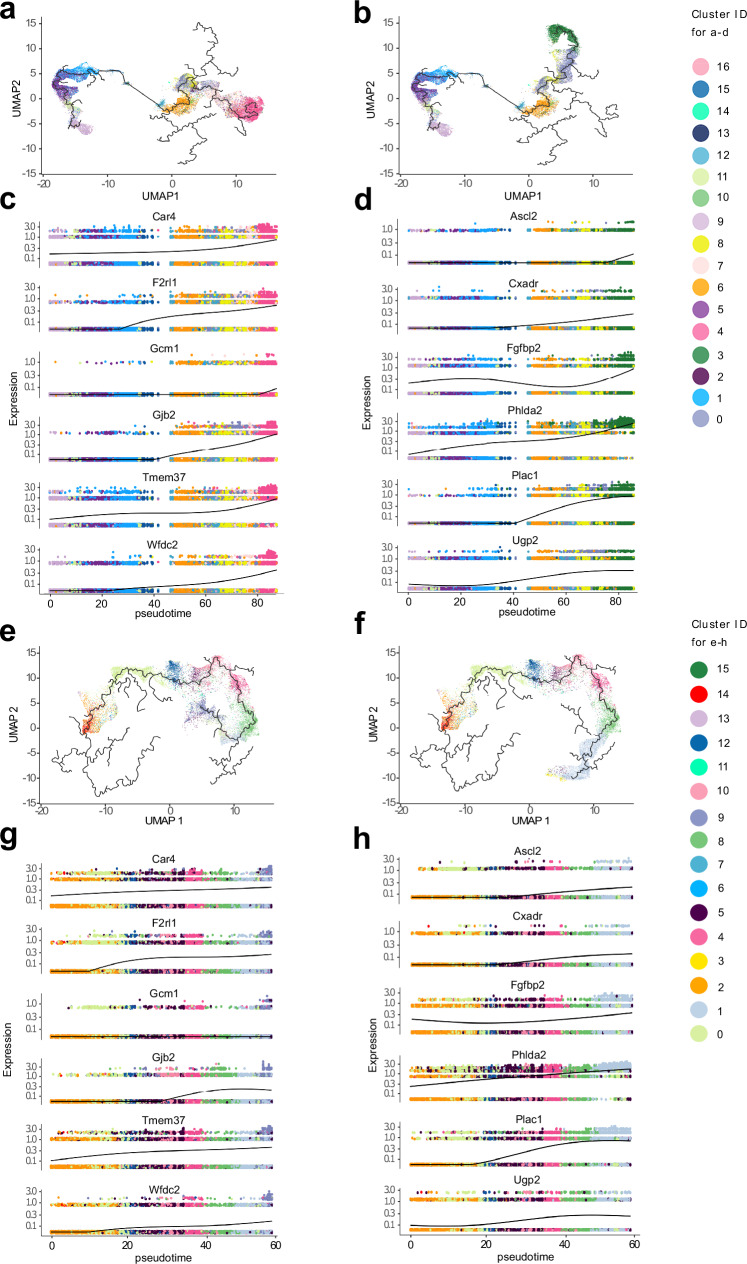
Pseudotime trajectories of Inhibit and Remove datasets. **a,b** JZP and LP trajectories can be defined from pseudotime. Analysis of branches in single cell pseudotime trajectories in Inhibit dataset. Pseudotime was calculated with Monocle 3 to measure how much progress an individual cell has made through a process of TSC differentiation. Cells were coloured by cluster identity. **a** LP trajectory in Inhibit dataset. **b** JZP trajectory in Inhibit Dataset. Branches were selected by identifying start node (cluster 9 with T0 cells) and end node (nodes at the end of JZP and LP identified clusters). **c, d** Expression of JZP and LP-specific genes in pseudotime across their trajectory branch. Branches of pseudotime trajectories were selected by assigning earliest principal node with highest number of t0 cells as a start and end principal node of differentiated JZP or LP cluster as end and connecting the shortest path between the points. Branches were then plotted on UMAP with cells coloured by cluster. Genes shown are a combination of known markers and ones from the top list. **c** Expression of LP genes across pseudotime in Inhibit dataset. **d** Expression of JZP genes across pseudotime in Inhibit dataset. **e** JZP trajectory in Remove dataset. **f** LP trajectory in Remove dataset. Branches were selected by identifying start node (cluster 14 with T0 cells) and end node (nodes at the end of JZP and LP identified clusters). **g** Expression of JZP genes across pseudotime in Remove dataset. **h** Expression of LP genes across pseudotime in Remove Dataset.

We used pseudotime trajectory analysis in Monocle to follow gene expression over TSC differentiation. The branches following the differentiation trajectories were drawn from TSCs to JZP and LP cells (Inhibit in Fig. 5a-b, Remove in Fig. 5e-f). We identified Monocle gene expression modules associated with the JZP and LP clusters (top genes shown on UMAP in Supplementary Fig. 12a-d for both Inhibit and Remove). We extracted well-known markers of JZP and LP identity from these modules and plotted them over the differentiation trajectories together with markers enriched in either population by Monocle Score (Fig. 5c-d, g-h). The Remove dataset showed an increase in JZP markers *Ascl2* and *Plac1* earlier in pseudotime than in the Inhibit dataset, which supports the rationale underlying our experimental design as well as our earlier data (Fig. 1c, d) that the Remove conditions favour JZP lineage differentiation.

To determine whether the regulons identified by SCENIC drive the expression of genes in the modules identified by Monocle, we cross-referenced the high confidence targets of E2F8 and PHF8 with the gene expression modules linked with LP identity. Among the targets of E2F8, 17 were present in the LP gene expression module (total 115 genes), including *Gjb2*, *Tmem150a*, *Wfdc2*, *Maged1*, a statistically highly significant enrichment (*p* = 1.68E-09 in a hypergeometric test) (Supplementary Fig. 13a). Those genes were also in the top 10 genes in the module by Monocle score. Interestingly, the targets of PHF8 included *E2f8* but also *Gcm1* and *Gjb2*. Hence, we conclude that E2F8 might define a transcriptional hub that is largely specific to SynT differentiation.

### Comparison between JZP and LP lineage-biased differentiation strategies

**Fig. 6:**
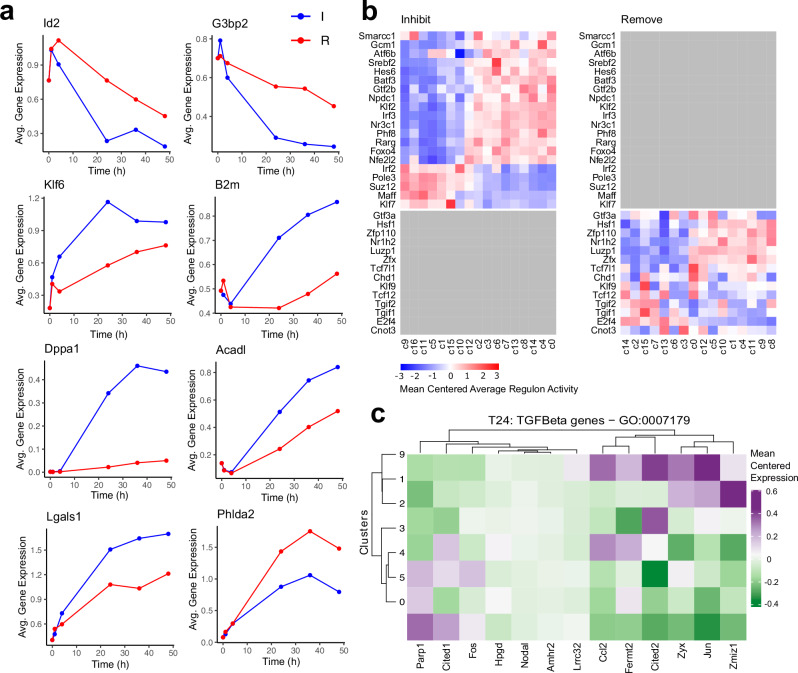
Comparison between Inhibit and Remove datasets. **a** Average expression of top markers between Remove (R) and Inhibit (I) datasets over time (**h**). Dots represent the average expression of the gene at each time point. Top 8 genes consistently higher in Inhibit and Remove were selected. Source data are provided as a Source Data file. **b** SCENIC Heatmap showing unique regulons for Remove and Inhibit datasets with clusters ordered by timepoint using Tempora. Colour represents average score for the activity of the regulon for each cluster. Grey fields are regulons absent in one of the datasets. All values were mean centred for each regulon. Inhibit regulons were hierarchically clustered and the same order was applied to the Remove dataset to allow for direct comparison of regulon activity. **c** Heatmap showing expression of detected TGFβ pathway-related genes (defined by GO:0007179) across Inhibit and Remove t24 clusters. Clusters that contain >85% cells from one treatment were selected. Gene list was filtered to include only marker genes (l2fc0.6) for t24 dataset. Genes and clusters were hierarchically clustered. Values were mean centred for each gene. Colour represents average expression of marker gene in a cluster.

Our experimental design provided an opportunity to compare the process of TSC differentiation through MEK inhibition (Inhibit) to withdrawal of FGF and CM (Remove), which is the more widely used method. We identified DEGs between Inhibit and Remove datasets with P_adj_ < 0.05 and 1.5 times fold-change and plotted the top 8 most differentially expressed genes over the time-course (Fig. 6a). The average expression of DEGs at each time point was altered more in Inhibit cells than in Remove, in line with the previously noted accelerated differentiation in Inhibit conditions. For example, the average expression of TSC marker *Id2* increased from t0 to t1 (like *Eomes*)^21^ and then decreased substantially at later time points under both differentiation conditions. However, it showed a much stronger decrease in MEK inhibitor-treated cells (Fig. 6a). Other markers that decreased in expression over the time course, such as *G3bp2* also showed a more rapid and greater decrease in the Inhibit condition. Conversely, the average expression of *Klf6*, a TF activated by TGFβ/Activin signalling^46^ was higher in Inhibit cells in which the TGFβ/Activin signal is retained in the supplemented CM. Similarly, other genes that increased in expression over the time course (*B2m*, *Dppa1, Acadl* and *Lgals1*) showed a stronger effect in the Inhibit cells^18^. Interestingly, the JZP marker *Phlda2* rose much faster in Remove cells, suggesting that its expression was supressed by TGFβ/Activin. This was the only gene revealed by this analysis that increased more dramatically in the Remove than in the Inhibit conditions, which overall confirmed that differentiation was biased towards the JZP path in Remove conditions.

We compared TF regulon activity in SCENIC between the Inhibit and Remove datasets (Fig. 6b and Supplementary Fig. 10). Most regulons identified were shared between the two datasets, but a subset of regulons was unique. Unique regulons whose scores increased with differentiation only in Inhibit were *Gcm1*, *Atf6b*, *Srebf2*, *Hes6*, *Batf3*, *Gtf2b*, *Npdc1*, *Klf2*, *Irf3*, *Nr3c1*, *Phf8*, *Rarg*, *Foxo4*, and *Nfe2l2*. As exemplified by *Gcm1*, these regulons are strong candidates for driving entry into the LP lineage and ultimately towards SynT differentiation. Regulons that uniquely decreased during differentiation in the Inhibit dataset included *Irf2*, *Pole3*, *Suz12*, *Maff*, and *Klf7*, making these particularly prevalent FGF/MAPK targets. Unique regulons in Remove that increased over time were *Hsf1*, *Zfp110*, *Nr1h2*, *Luzp1*, *Zfx* and *Tcf7l1*, while those that decreased were *Tcf12*, *Tgif2*, *Tgif1* and *E2f4*. To corroborate this finding, we treated TSCs under Remove conditions with rosiglitazone to block JZP differentiation^47^ and found much-reduced expression levels of the identified markers as well as of *Tpbpa* as a junctional zone-expressed control (Supplementary Fig. 13b). These data indicate that these regulons are likely governed by the Activin/TGFβ signalling cascade and may be involved in junctional zone formation.

One of the main differences between the two datasets was that the Inhibit cells were expected to maintain TGFβ pathway activity due to the continued presence of CM, while MEK was inhibited. The t24 time point was the earliest one where a large difference was observed in TF activity. Therefore, we extracted data from all cells from the t24 time point and clustered them separately at res 0.4. They formed 10 clusters out of which clusters 0, 5 and 4 were composed of predominantly Remove cells and clusters 1, 2, 3 and 9 of Inhibit cells (Supplementary Fig. 14, Supplementary Data 21). Using these clusters, we plotted expression levels of genes that fall into the gene ontology term GO:00007179 (transforming growth factor beta receptor signalling pathway) and found that they segregated by differentiation method (Fig. 6c). The expression of genes detected in the dataset that promoted TGFß signalling such as *Jun*, *Ccl2*, *Cited2*, and *Zyx* was largely elevated in Inhibit clusters and decreased in Remove. Collectively, these data reveal multiple nodes of gene networks that likely govern JZP and LP formation.

### JZP and SynT-I precursors share similar differentiation trajectories

Although the pseudotime trajectory analysis in Monocle clearly identified a LP path demarcated by *Gcm1* expression, *Gcm1* is a precursor gene of only one specific SynT subtype, the SynT-II layer that faces the fetal endothelial cells in the placental labyrinth^48^. Other genes in this cluster (c4 in Fig. 3a) were also associated with a SynT-II-specific expression (Supplementary Fig. 15a). Hence, we set out to confirm the SynT-II specificity of this particular LP branch by assessing additional genes that we found enriched in this cluster. To this end, we performed a differentiation time course experiment of TSCs under standard differentiation conditions (Remove) and in the presence of Chiron99021 (CHIR), which specifically induces TSC differentiation towards the SynT-II lineage^49^, and profiled the expression of *Gabrp*, *Abcb1* and *Gjb2* (Supplementary Fig. 15b). The expression of these genes was strongly up-regulated in the CHIR-treated cells, indicating SynT-II specificity of gene expression. We also stained placentas for EMB, another c4-specific gene, in conjunction with SynT-II marker MCT4 (encoded by the *Slc16a3* gene) and found perfect overlap in staining patterns (Supplementary Fig. 15c). Conversely, double staining with SynT-I marker MCT1 highlighted the close juxtaposition but non-overlap of both syncytial layers (Supplementary Fig. 15c). These data confirmed that Inhibit cluster 4 demarcates the SynT-II differentiation trajectory specifically. Hence, c4-enriched genes are markers of SynT-II, including *Abcb1*, *Gabrp* and *Emb*.

**Fig. 7:**
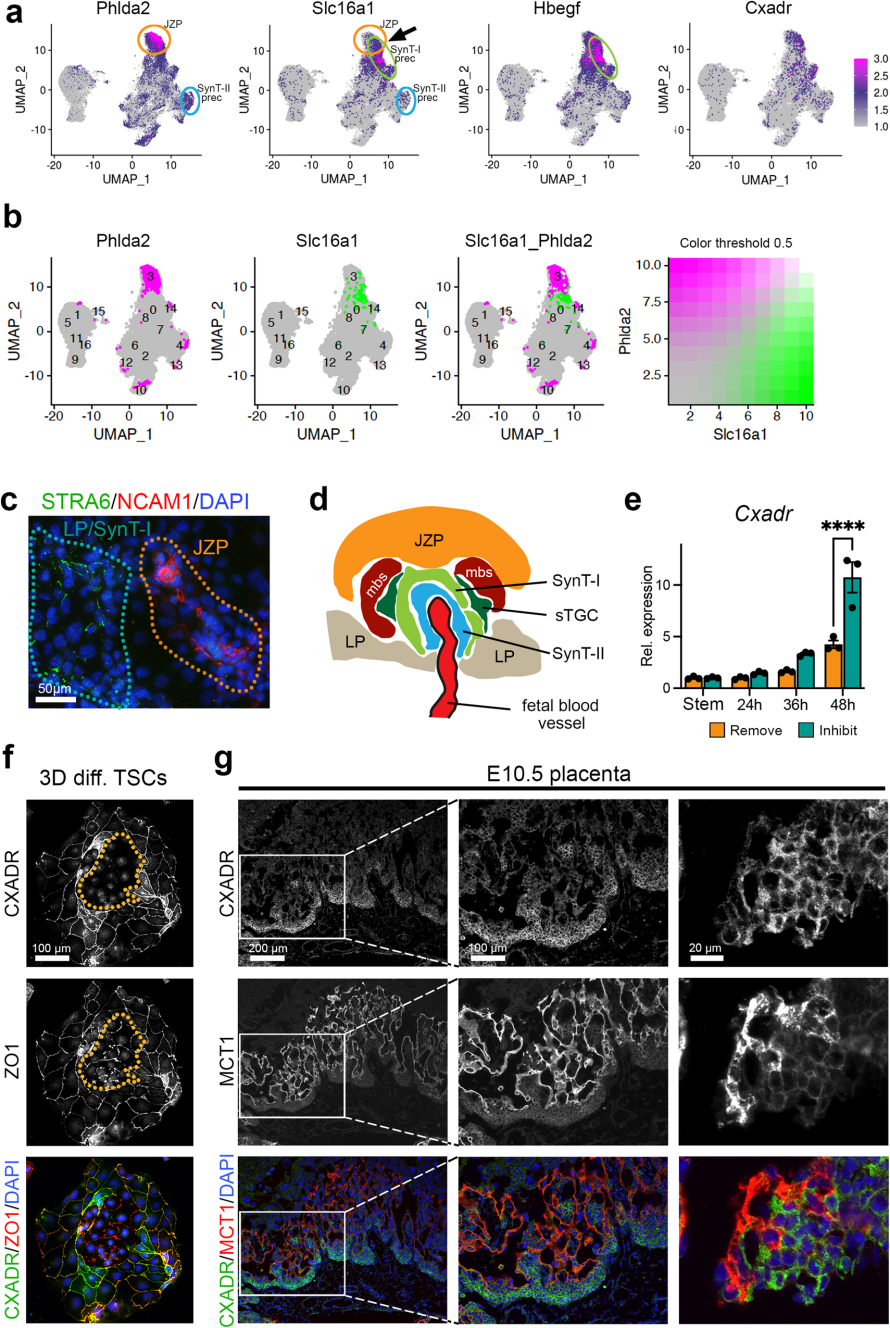
Expression dynamics of labyrinth progenitor marker CXADR. **a** UMAP plots of the Inhibit dataset of genes enriched in the presumptive JZP cluster. *Phlda2* is a well-known marker of the JZ. Yet SynT-I markers *Slc16a1* and *Hbegf* are enriched in the same or adjacent cluster (arrow), as is *Cxadr*. Colour gradient shows normalised expression. **b** UMAP plots of locations of cells with top 10% expression of *Phlda2*, *Slc16a1* and overlap between the two cell populations. **c** Immunostaining for SynT-I marker STRA6 (green) and junctional zone marker NCAM1 (red) reveals non-overlap between individual LP and JZP cells entering these lineages. Staining was carried out after 48 h of culture in Inhibit conditions. Image is representative of three independent experiments. **d** Diagram depicting the organization of emerging trophoblast cell types at sites of fetal vessel invagination into the chorionic ectoderm. JZP = junctional zone precursor, mbs = maternal blood space, sTGC =sinusoidal trophoblast giant cell, LP = labyrinth precursor. **e** *Cxadr* expression dynamics across the 48 h differentiation time course in Inhibit and Remove conditions. During these early stages of differentiation, *Cxadr* expression is up-regulated, in particular in Inhibit conditions that promote LP differentiation. Data are normalized to stem cell conditions and plotted as mean +/- SEM of three independent replicates. Statistical significance was calculated using 2-way ANOVA. **** *p* < 0.0001. Source data are provided as a Source Data file which contains exact *P* values. **f** Representative image of differentiated TSCs at 3 days (3D) of differentiation stained for CXADR (green) and cell membrane marker ZO1 (red). The encircled area highlights early-stage syncytializing cells that have lost CXADR while still retaining some ZO1. Image is representative of three independent experiments. **g** E10.5 placenta stained for CXADR (green) and MCT1 (red) showing the immediately adjacent localisation of CXADR-positive single cells to syncytial MCT1-positive cells. Images are representative of *n* = 4 placentas. The middle column of images depicts a higher magnification of the boxed area in the left column. The right-hand column of images depicts an area at the base of the placental labyrinth taken from a consecutive section of that shown on the left. Images are representative of three independent samples.

Our specific interest, however, was triggered by the observation that the JZP trajectory (c3 in Fig. 3a), demarcated by well-known hallmark genes such as *Phlda2* (Fig. 7a), *Ascl2* (Fig. 5d) and *Gjb3* (Fig. 3j), seemed to be very similar to, and partially overlapping with, SynT-I specific genes such as *Slc16a1* (*Mct1*) and *Hbegf* (Fig. 7a, Supplementary Fig. 15d)^50^. We further explored this unexpected similarity bioinformatically and found that while markers like *Phlda2* and *Slc16a1* are co-expressed, the cells with the highest expression (top 10%) of *Phlda2* and *Slc16a1* are two adjacent but not overlapping populations (Fig. 7b). This was further confirmed by cell staining of differentiating TSCs in which cells adopting JZP or LP fate remain distinct (Fig. 7c). In vivo, SynT-I cells emerge at the tip of the chorionic folds formed by invaginating fetal blood vessels, in relatively close physical proximity to JZP^51^, whereas SynT-II cells differentiate underneath, closer to the chorionic side (Fig. 7d). In line with this anatomical organisation, our data raise the intriguing possibility that early SynT-I precursors are more closely aligned with JZP than with *Gcm1*-positive SynT-II precursors during TSC differentiation.

Next, we wanted to pursue this observation that early SynT-I precursors share a similar differentiation trajectory to JZP. Given the fact that *Slc16a1* (*Mct1*) and *Hbegf* are widely used markers specific to SynT-I cells in the murine labyrinth^50,52^ but clustered in the UMAPs in a cell group partially overlapping with JZP markers such as *Phlda2*, we asked whether other genes that shared a similar cluster enrichment as *Slc16a1* were also LP markers. Interrogating the Monocle modules to this effect identified the Coxsackie virus and adenovirus receptor *Cxadr* as one such candidate (Fig. 7a, Supplementary Fig. 15d)^53^. Indeed, *Cxadr* has been identified in a recent single nuclei RNA-seq study of mouse placentas as labyrinth precursor gene that was most highly enriched in the earliest stages of labyrinth formation at E9.5 (Supplementary Fig. 15e)^6^. This was corroborated by another study^7^ where *Cxadr* was identified as marker of labyrinth trophoblast progenitors. Yet in our pseudotime analysis, *Cxadr* followed closely the pattern of other JZP genes (Fig. 5d).

To further characterize the expression dynamics of *Cxadr*, we profiled its expression levels across the 48 h Remove-Inhibit differentiation time course. *Cxadr* was up-regulated as differentiation progressed, in particular in the Inhibit conditions that are LP-enriched (Fig. 7e). Extended TSC differentiation time course experiments in standard Remove conditions revealed that *Cxadr* peaked at 3 days (3D) of differentiation, preceding the onset of overt cellular syncytialization (Supplementary Fig. 15f). Upon enforced syncytialization which can be achieved by treating TSCs with the WNT activator CHIR, *Cxadr* expression declined, in line with our observations that fusing cells down-regulate CXADR protein (Supplementary Fig. 15g). These data corroborate the notion of *Cxadr* as a LP gene.

Immunostaining of 3D-differentiated TSCs showed membrane localization of CXADR which co-localized with the membrane marker ZO1 (Fig. 7f). Intriguingly, in areas where cells started to fuse, membrane-localized CXADR staining was lost prior to the onset of membrane breakdown as indicated by the changes in ZO1 staining pattern from a smooth cellular outline of single cells to a ruffled appearance and then to a merely punctate staining of membrane remnants (Fig. 7f). In the E10.5 placenta, CXADR staining was strongest in the cuboidal epithelial cells of the chorionic ectoderm at the base of the forming labyrinth and in smaller cell patches within the forming labyrinth (Fig. 7g), which is fully in line with previous observations^53^. Double staining with SynT-I and -II markers MCT1 and MCT4, respectively, showed a transition of CXADR-positive single cells to MCT1- or MCT4-positive mature syncytial cells, in a mutually exclusive staining pattern (Fig. 7g, Supplementary Fig. 15h). These data confirmed that CXADR may mark an LP population both in TSCs in vitro and in the forming placenta in vivo.

### *Cxadr* ablation in TSCs reveals a role in LP cells to control SynT formation

**Fig. 8:**
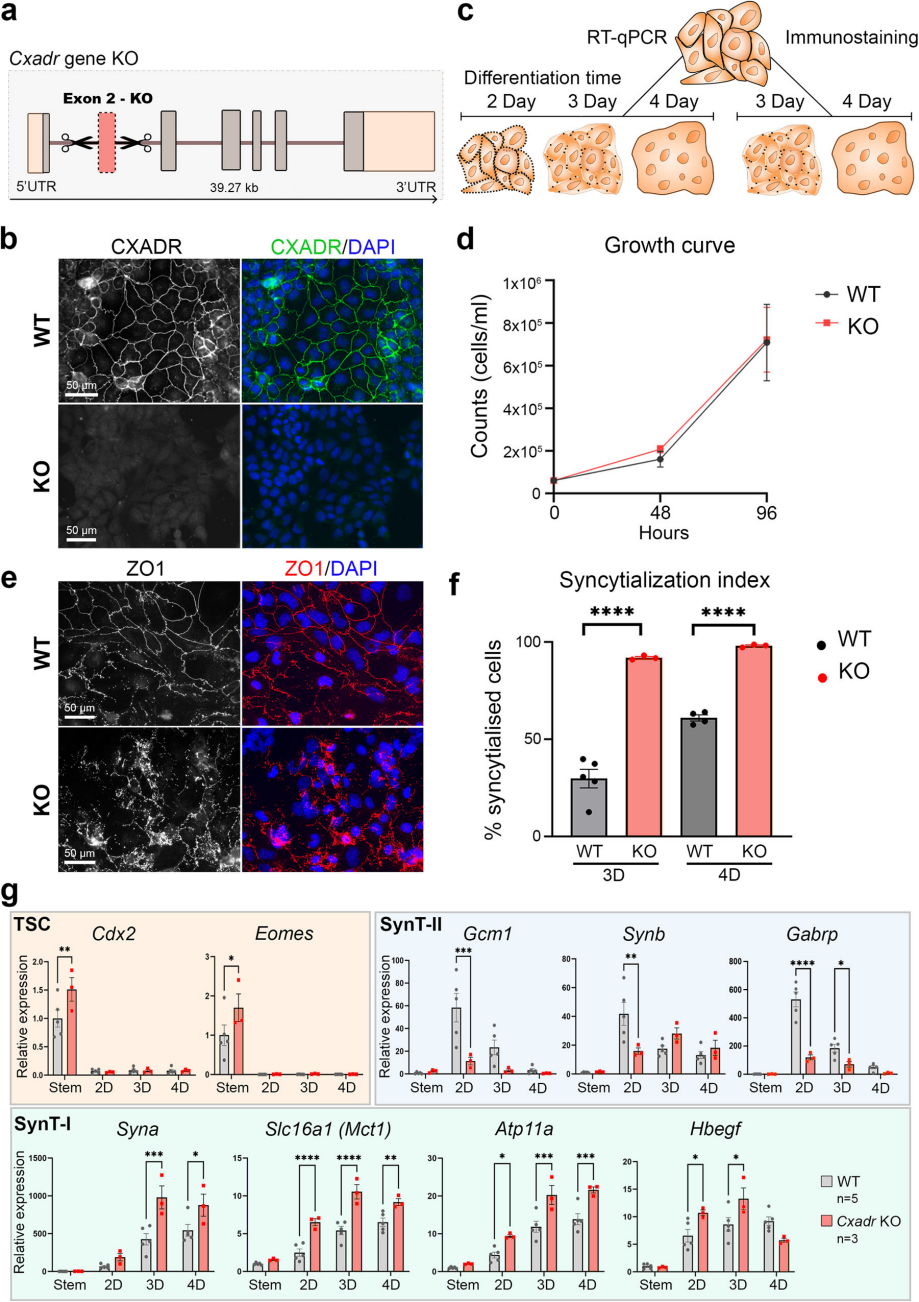
Functional characterization of CXADR. **a** Schematic depicting the generation of *Cxadr*-null TSCs by CRISPR-Cas9 targeting of exon 2. **b** Immunofluorescence staining of wild-type (WT) and knockout (KO) TSCs against CXADR, showing cell membrane localisation in WT cells, and lack of positive staining in KO TSCs. Images are representative of *n* = 5 WT independently derived clones and n = 3 independently derived KO clones. **c** Diagram depicting experimental set-up, where WT and KO TSCs were used for RT-qPCR and immunofluorescence staining across a differentiation time course. **d** Proliferation rates of WT *(n* = 3 clones) and KO (*n* = 3 clones) TSCs in differentiation conditions (Remove) over a 4-day period. No differences were observed. Statistical analysis was performed by two-sided unpaired t-test per day. Data are displayed as mean +/- SEM. **e** Immunofluorescence staining of WT and KO TSCs after 4 days of differentiation against cell membrane marker ZO1, depicting the presence of a smooth continuous ZO1-stained cell border in non-syncytial, single cells in the WT clone, and complete syncytialisation in the KO clone. Images are representative of n = 4 WT clones and *n* = 3 KO clones. **f** Bar chart showing the significant increase in syncytialized cells in KO (*n* = 3 clones) compared to WT TSCs at 3 days (3D) and 4 days (4D) of differentiation (WT *n* = 5 TSC clones at 3D and n = 4 at 4D). Statistical significance was calculated using two-sided unpaired t-test; data are plotted as mean +/- SEM. **** p < 0.0001. **g** RT-qPCR analysis of wild-type (WT, *n* = 5 clones) and *Cxadr* knockout (KO, *n* = 3 clones) TSC clones grown in stem cell conditions and after 2-, 3-, and 4-days of differentiation in Remove conditions. Genes assessed included TSC markers (*Cdx2*, *Eomes*), and markers of the syncytiotrophoblast layers II ( = SynT-II; *Gcm1*, *Synb*, *Gabrp*) and SynT-I (*Syna*, *Slc16a*/*Mct1*, *Atp11a*, *Hbegf*). Data are normalized to WT TSCs in stem cell conditions and plotted as mean +/- SEM. Statistical significance was calculated using 2-way ANOVA. * *p* < 0.05, ** *p* < 0.01, *** *p* < 0.001, **** *p* < 0.0001. Source data and full P values for d, f, and g are provided as a Source Data file.

To further investigate the potential function of CXADR in LP cells, we generated *Cxadr* KO TSCs by deleting exon2 using CRISPR-Cas9 (Fig. 8a, Supplementary Data 22)^50,54,55^. Successful deletion of the exon was confirmed by genotyping PCR and by RT-qPCR (Supplementary Fig. 16a, b), and the absence of a functional protein was ascertained by immunostaining (Fig. 8b). We then assessed the impact of *Cxadr* deletion on TSC behaviour in stem cell conditions and upon differentiation (Fig. 8c). *Cxadr* KO TSCs appeared indistinguishable from their wild-type (WT) counterparts when grown in regular TSC conditions. Although the nuclei of *Cxadr* KO TSCs appeared reduced in size, proliferation rates remained unchanged (Fig. 8d). However, when induced to differentiate using standard Remove conditions, we observed many more fused cells in *Cxadr* KO TSCs, as indicated by the absence of ZO1-demarcated cell boundaries (Fig. 8e). Quantification of fused versus single cells after 3D and 4 days (4D) of differentiation indeed demonstrated an enormous increase in fusion rates in *Cxadr*-null TSCs (Fig. 8f).

To assess the molecular changes accompanying syncytialization, we quantified trophoblast cell type-specific marker gene expression by RT-qPCR in WT and KO TSCs grown in stem cell conditions and after 2 days (2D), 3D and 4D of differentiation. This analysis demonstrated that in the absence of *Cxadr*, SynT-I markers *Atp11a*, *Hbegf*, *Slc16a1* (*Mct1*) and *Syna* were significantly up-regulated (Fig. 8g). TSC markers as well as LP markers *Met*, *Rhox4b* and *Epcam* also exhibited higher levels in KOs compared to WT cells (Supplementary Fig. 16c). In conditions which favour LP or SynT-II (Inhibit or CHIR, respectively), *Gcm1*, a marker of SynT-II precursors, was more rapidly induced at earlier time points. However, a lasting enrichment of SynT-II cells was not observed (Fig. 8g, Supplementary Fig. 16d). Instead, at 2D of differentiation in Remove conditions, *Gcm1* and *Synb* expression was significantly lower in *Cxadr* KO than in WT cells (Fig. 8g). These data are in line with the reported overabundance of mature SynT-I and the depletion of SynT-II cells in *Cxadr* mutant placentas^53^. JZP and TGC markers remained unchanged between WT and KO across the differentiation time course (Supplementary Fig. 16c). Overall, these data demonstrated that CXADR is a marker of single-cell LP cells that is down-regulated prior to cell fusion and critically regulates the formation of mature labyrinth cells.

**Fig. 9:**
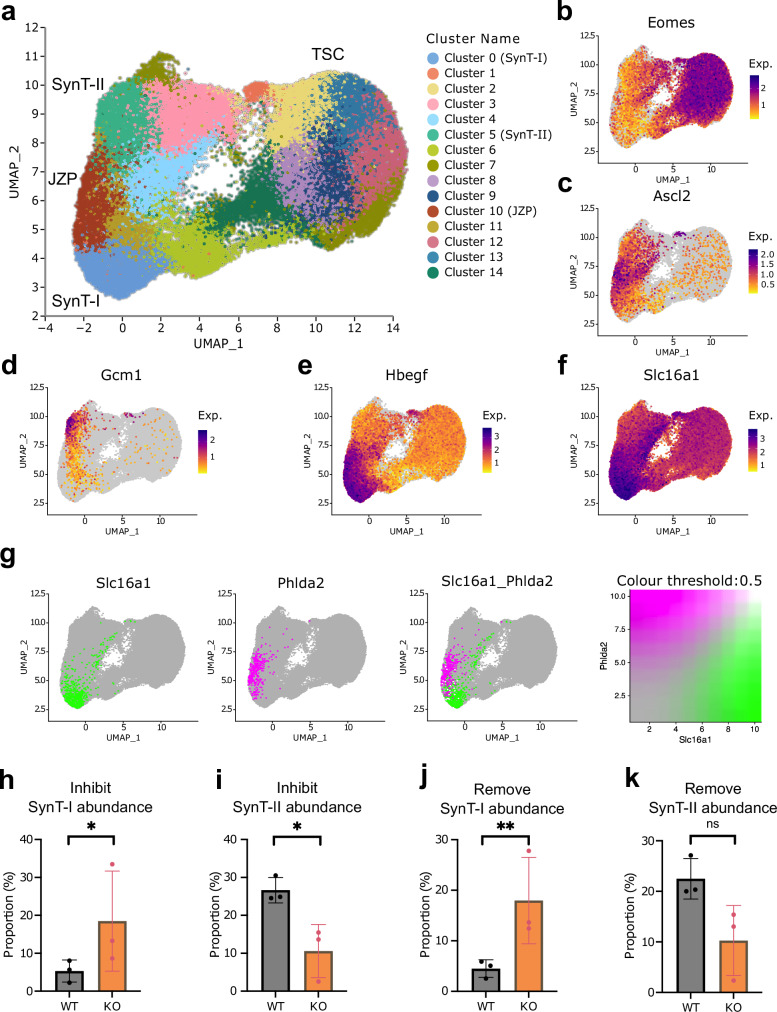
scRNA-seq of *Cxadr* KO TSCs. **a** UMAP of Inhibit dataset at t0 and t24 containing WT and *Cxadr* KO cells (resolution 0.7). Cluster 5 is identified as SynT-II, Cluster 0 as SynT-I, Cluster 10 as JZP. **b** UMAP plot of *Eomes* expression. **c** UMAP plot of *Ascl2* expression. **d** UMAP plot of *Gcm1* expression. **e** UMAP plot of *Hbegf* expression. **f** UMAP plot of *Slc16a1* expression. Exp = Normalised expression **g** UMAP plot of the locations of the top 10% cells by *Slc16a1* (green) and *Phlda2* (magenta) gene expression in the Inhibit dataset and the overlap between those populations (white). **h** Proportion of SynT-I cells (cluster 0) in the Inhibit dataset split by sample of origin. *p* = 0.035. **i** Proportion of SynT-II cells (cluster 5) in the Inhibit dataset split by sample of origin. *p* = 0.016. **j** Proportion of SynT-I cells (cluster 13) in the Remove dataset split by sample of origin. p = 0.007. **k** Proportion of SynT-II cells (cluster 6) in the Remove dataset split by sample of origin. *p* = 0.094. Data shown in **h**–**k** are mean +/- SD and n = 3 independently derived cell clones. * <0.05, ** <0.01, ns = not significant in an empirical Bayes framework moderated two-sided t-test using the *propeller* function from R package speckle (see Methods). Source data are provided as a Source Data file.

To further investigate the effect of *Cxadr* ablation on TSC differentiation dynamics, we performed scRNA-seq on three independent WT and *Cxadr* KO clones each. We analysed these at t0 (in the stem cell state) and after 24 hours of Inhibit or Remove treatment. We generated scRNA-seq data from approximately 100,000 cells sequenced to a mean depth of approximately 60,000 reads per cell. Data from the Inhibit treatment are presented in Fig. 9 and Supplementary Fig. 17a and data from the Remove treatment are presented in Supplementary Fig. 17b and Supplementary Fig. 18. In the UMAP (Fig. 9a, Supplementary Fig. 17a-b, Supplementary Fig. 18a) the t0 cells (TSC) cluster together and separate from the t24 cells (SynT-I, JZP, SynT-II). Next, we determined the expression levels of known lineage markers in the clusters (Fig. 9b-f, Supplementary Fig. 18b-f) and used these to assign labels to the key clusters. Among the more differentiated cells, clusters can clearly be identified as containing JZP, SynT-I and SynT-II progenitor cells (Fig. 9a, Supplementary Fig. 18a). These cluster assignments were further supported by the expression of marker genes in individual clusters when displayed as dot plots (Supplementary Fig. 18h-i).

Importantly, this additional analysis corroborated our Drop-seq data insofar as we again observed a juxtaposition of the JZP and SynT-I clusters (Fig. 9a, g and Supplementary Fig. 18a, g). At the onset of differentiation some cells in these clusters co-expressed *Phlda2* (JZP marker) and *Slc16a1* (SynT-I marker), however, the more differentiated cells (i.e., those with the highest transcript levels) were discrete and non-overlapping, akin to our observations in the Drop-seq data (Figure 7b, Fig. 9g and Supplementary Fig. 18g). These data strongly support the notion that early SynT-I precursors are more closely aligned with JZP than with *Gcm1*-positive SynT-II precursors during early TSC differentiation.

To further examine our observation of the biased differentiation of *Cxadr* KO TSCs towards SynT-I, we examined the proportions of WT and *Cxadr* KO TSCs in the SynT-I and SynT-II progenitor clusters under both Inhibit and Remove conditions (Fig. 9h-k and Supplementary Fig. 18j-k). Intriguingly, the *Cxadr* KO cells have approximately 3 times as many SynT-I progenitor cells and approximately half the number of SynT-II progenitor cells compared to the WT cells under both differentiation conditions. This data confirms that loss of CXADR indeed favours SynT-I differentiation at the expense of SynT-II differentiation.

## Discussion

In this study, we set out to identify molecular hallmarks of early cell lineage entry points as mouse TSCs exit self-renewal and start to diverge into specific trophoblast subtypes. We applied two differentiation strategies to induce lineage bias and performed single cell transcriptomic analyses across a time course between 0-48 h to map out the emerging cell fates. Our approaches identify candidate factors that likely contribute to the stem cell state of TSCs, as demonstrated by the example of *Nicol1* that we find is essential for TSC maintenance. By extrapolation, lineage marker genes and regulons identified as part of this study may equally prove to play important roles in specifying defined differentiation trajectories of placental trophoblast cells. For instance, the *E2f8*-directed regulon was particularly prevalent in the LP SynT-II branch, which is consistent with the demonstrated importance of *E2f7*/*E2f8* in the placental labyrinth^56–58^.

Surprisingly, we find that despite the morphological heterogeneity of TSCs in the stem cell state, their transcriptomic profiles appear remarkably similar to each other, with only a small subset of cells that has already started to differentiate and to change its cell cycle dynamics. Using signature genes identified in our study, we demonstrate that these cells can be readily identified in TSC cultures by up-regulation of KRT18. These findings are consistent with single-cell cloning and colony assessment studies that have equally revealed a fraction of cells within TSC cultures that have lost full stem cell potential^22^. However, the lack of overt cell clustering in our scRNA-seq data has interesting implications, insofar as the commonly observed variations in cell-to-cell abundance of several key TSC transcription factors may be a consequence of post-transcriptional or post-translational events. This highlights a frequently overlooked aspect, which pertains to the critical regulation of many, if not most, stem cell transcription factors by post-translational mechanisms^59,60^.

Recent scRNA-seq and snRNA-seq analyses of the developing placenta have profiled cellular fate trajectories^6,7^. However, these approaches have remained restricted to cells during later stages of differentiation, i.e. capturing developmental time windows when the vast majority of trophoblast cells are no longer in a stem cell state^50,61^. This makes it difficult, if not impossible, to outline trajectories of early lineage entry points. TSCs constitute a major advantage in this regard, as they can be maintained in a self-renewing stem cell state in the presence of FGF and TGFβ/Activin (the latter routinely supplied as CM), while withdrawal of these components or inhibition of their signalling action causes TSC differentiation. Here, we employed two different differentiation protocols that were aimed at biasing differentiation towards the JZP and LP routes. Our data demonstrate that this expectation was indeed met, with a particular enrichment of JZP differentiation in Remove conditions, whereas LP differentiation was favoured in Inhibit conditions.

This experimental design allowed us to resolve the differentiation trajectories into the various trophoblast cell types at a greater degree of resolution, in particular during the earliest phases of cell fate specification. MAPK signalling is known to be of particular importance for SynT-II differentiation^52^. This was corroborated by our Inhibit data that exhibited a clear bias towards the SynT-II path, which was confirmed by the expression of fibronectin receptor Embigin, a marker of the SynT-II lineage, which was initially identified in the SynT-II cluster of a previous study^6^. Here, we prove the SynT-II specificity of Embigin expression.

**Fig. 10:**
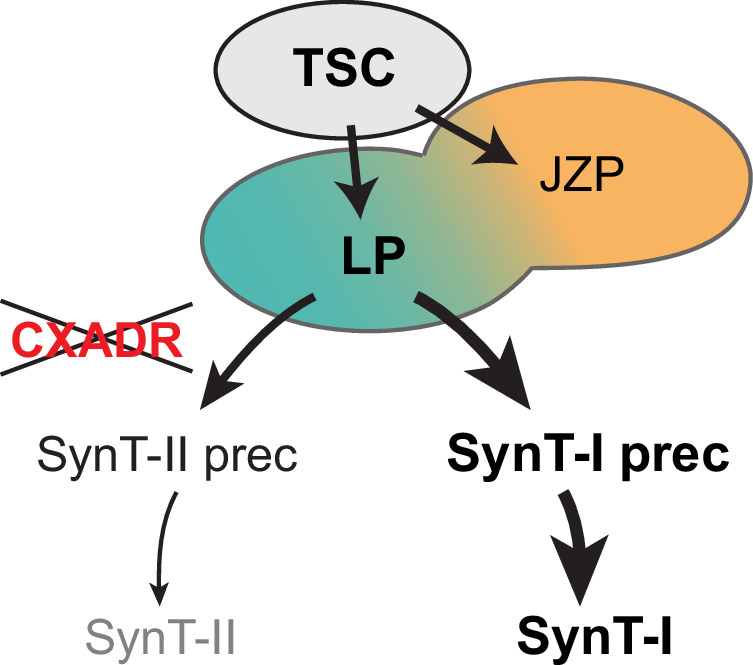
Model of early trophoblast stem cell (TSC) differentiation and CXADR function in regulating trophoblast cell fusion dynamics. TSCs differentiate along the two main routes into junctional zone progenitors (JZP) and labyrinth progenitors (LP). Open circles between LP and JZP indicate partially shared differentiation trajectories, specifically between JZP and SynT-I precursors. CXADR was identified as a factor critical for regulating the cell fusion dynamics during subsequent differentiation into syncytiotrophoblast (SynT) cells. *Cxadr* KO TSCs display an enhanced differentiation towards SynT-I cells, a prolonged retention of TSCs and labyrinth progenitor (LP) (shown in bold), while differentiation into JZP and other trophoblast subtypes remains unchanged.

Perhaps most surprisingly, however, we find that the early paths towards SynT-I and SynT-II specification diverge significantly, with SynT-I cells being more closely aligned with JZP (Fig. 10). We observe this cell lineage alignment in the two independent scRNA-seq datasets presented in the current study, thus strongly corroborating this lineage juxtaposition. This is an intriguing observation that is consistent with the previous notion of some shared transcription factors between SynT-I precursor cells and cells of the ectoplacental cone, a structure that goes on to form the junctional zone^7^. This observation is further underpinned by the anatomical organization of these layers, where emerging SynT-I cells are directly juxtaposed to JZP (Fig. 7d). The single cell pseudotime trajectories in our current study imply that SynT-I precursors and JZP may also share similar signalling cues.

Amongst the genes that clustered within this partially overlapping population of JZP and SynT-I precursors was the Coxsackie virus and adenovirus receptor *Cxadr*, a presumptive LP gene. Therefore, we investigated the role of CXADR in junctional zone and/or labyrinth differentiation in greater detail. The impact of CXADR ablation has been studied previously in mice and caused embryonic lethality at E11.5 with severe placental labyrinth defects. However, these placental pathologies were specifically deemed to be induced by defective endothelial cells^53^. In line with these previous reports, we also find that CXADR is expressed in labyrinth progenitor cells at the base of the chorionic plate at mid-gestation where it demarcates the membranes of trophoblast cells prior to cell fusion, immediately adjacent to maturing MCT1 and MCT4-positive SynT-I and SynT-II cells, respectively. However, in contrast to the previous data, we now identify a critical role for CXADR in the trophoblast lineage itself.

Tracing CXADR membrane localization in TSCs shows that its down-regulation precedes the breakdown of cell membranes prior to cell fusion. These data are in line with our functional data that demonstrate a regulatory role of CXADR in controlling trophoblast cell fusion dynamics. Thus, *Cxadr* KO results in increased numbers of syncytiotrophoblast. While overall fusion rates are elevated, CXADR appears to specifically control the entry point into the SynT-I differentiation route. As a consequence, *Cxadr*-ablated TSCs fuse excessively, and predominantly into SynT-I cells, a finding we confirm both on the molecular and on the single cell level (Fig. 10). This observation is entirely consistent with the prevalence of MCT1-positive SynT-I cells but near-absence of MCT4-positive SynT-II cells in *Cxadr*-null placentas^48,51,53^. As a tight junction-associated protein, CXADR may normally prevent cell fusion by strengthening cell membrane integrity. Yet, how CXADR has a more prominent (inhibitory) effect on SynT-I fusion remains to be determined. Importantly, we identify a role for CXADR as a protein that functions to balance differentiation rates of the two syncytial layers of the mouse labyrinth, thereby ensuring that adequate cell numbers are maintained between LP and maturing SynT cells.

Importantly, previous *Cxadr* KO studies in mice suggested that the placental defects are secondarily induced by endothelial cell failure^53^. This was despite the fact that *Cxadr* is explicitly not expressed in fetal endothelial cells of the placental labyrinth. These data were based on the analysis of conditional KO (cKO) conceptuses in which the gene was ablated only in the embryonic lineages, but not in trophoblast, using the *Sox2*-Cre driver^53,62^. These cKOs still died around E11.5 with heart and placental defects, similar to the constitutive KO. To explain their findings, the authors inferred an influence by other embryonic lineages and/or from the yolk sac that may signal to the forming labyrinth and cause these defects. However, this indirect conclusion does not necessarily rule out that a gene may also have an important role in the trophoblast lineage proper. Indeed, our data demonstrate that CXADR functions cell-autonomously in trophoblast cells and that its ablation causes an apparent imbalance in syncytiotrophoblast differentiation. These insights strengthen the notion of functional studies to be conducted in the cell type of interest to rule out pleiotropic effects and interactions between cell lineages. In the context of placentation, this means to knock out a gene in the trophoblast lineage directly - a goal that has long been hampered by the absence of reliable Cre lines to drive gene deletion in the early trophoblast lineage^63^ but that has recently been overcome with the generation of *Sox2*-Flp mice that can achieve this desired genetic constellation when using the widely available knock-out first alleles from the KOMP/EUCOMM consortium^50^.

The finely tuned and tightly controlled regulation of early cell fate specification events is critical for embryogenesis and tissue formation, and as such our findings will have important implications for human developmental processes. In general, these processes dictate the establishment of precursor cell pools available for further differentiation, thereby laying the foundations for future organ anatomy and function. Deviations from optimal progenitor cell numbers often has detrimental consequences. Formation of the placenta is a key process where this particularly matters, especially for the establishment of the placental exchange surface^55,64^.

The pertinence of our data for gaining a better understanding of developmental processes in health and disease is exemplified by the candidate factors identified in our analyses. While no studies have been conducted on *Nicol1* in the context of placental development, its importance has been demonstrated in the brain and male reproductive organs. *Nicol1* is one of three genes within a DNA microdeletion identified in a patient with a mild form of Wolf-Hirschhorn syndrome^65^. Based on its expression in developing and adult cortical and subcortical mouse neurons, *Nicol1* has been suggested to have a role in the etiology of intellectual and fine-motor disabilities. It is also identified as a secreted peptide involved in lumicrine-mediated sperm maturation in the epididymis, and male mice lacking *Nicol1* are infertile^28^. NICOL1 has also been shown to enhance renal fibrosis in mice by interacting with and stabilising mRNAs of profibrotic molecules, an effect which was reversed upon *Nicol1* ablation in mice^66^. These findings suggest that *Nicol1* has distinct roles in different organs; our study highlights its importance in maintaining the stem cell state of mouse TSCs in the extra-embryonic compartment.

The virus receptor CXADR has been implicated in various cardiac conditions in humans including in the pathogenesis of myocarditis and dilated cardiomyopathy^67–69^. CXADR is also involved in the development of congenital heart defects^69,70^, and based on our data presented here, a contributory role of the placenta in these defects is conceivable^50,53^. As in the mouse, CXADR is absent from mature syncytiotrophoblast cells in the human placenta, and this lack of expression has been postulated to limit the transplacental transmission of viral pathogens. By contrast, invasive extravillous cytotrophoblast cells continue to express CXADR and are susceptible to adenovirus-induced cell death^71^, a pathology that will predispose the pregnancy to adverse reproductive outcomes, notably preeclampsia and even miscarriage. In addition, CXADR is downregulated in trophectoderm of hatched blastocysts while ZO1 remained present – a pattern identical to what we observed in TSCs prior to cell fusion^72^. These examples highlight the importance of CXADR during critical periods of development and emphasize the relevance of our data in providing pivotal insights into the molecular regulation of trophoblast cell type-specific differentiation pathways^1,73,74^.

## Methods

### Placenta collection

Placentas were collected from timed pregnancies of C57BL/6 N crosses. The date of the vaginal plug was considered E0.5. All animal work was conducted with approval by the University of Calgary’s animal care committee, and with appropriate Health Sciences Animal Care Committee (HSACC)-approved animal use protocols in place (protocol numbers AC22-0147 and AC22-0118). Mice were housed in IVC cages with 12 hour light-dark cycles under ambient temperature ( ~ 22 °C) and humidity conditions. The age range of the animals used in this study was 8-20 weeks. Euthanasia of pregnant females was conducted as stipulated in the animal care protocol, using isoflurane followed by cervical dislocation.

### Cell culture

TSC lines used in this study were TS-EGFP and TS-Rs26, both a kind gift of the Rossant laboratory (Toronto, Canada)^8^. For single-cell RNA sequencing, the TS-EGFP line was used throughout. The *Cxadr* and *Nicol1* KO TSC lines were generated in TS-Rs26 cells. TSCs were routinely cultured as previously described^8,44,50^. Briefly, TSCs were cultured in Full Medium consisting of 20% fetal bovine serum (FBS) (Wisent 098150), 1 mM sodium pyruvate (ThermoFisher Scientific 11360-039), 1x Anti-mycotic/Antibiotic (ThermoFisher Scientific 15240-062), 50 μM 2-mercaptoethanol (Gibco 31350), 37.5 ng/ml bFGF (Cambridge Stem Cell Institute), and 1 μg/ml heparin in RPMI 1640 with L-Glutamine (ThermoFisher Scientific 21875-034), with 70% of the medium pre-conditioned on mouse embryonic fibroblasts (CM). The medium was changed every two days, and cells passaged before reaching confluency. Trypsinization was carried out with 0.25% Trypsin/EDTA (ThermoFisher Scientific 25200-056) at 37 °C for about 5 min.

For the scRNAseq timecourse experiment differentiation of TSCs was induced through the withdrawal of conditioned medium and bFGF (Remove), or by inhibition of the MAPK pathway by addition of the selective MEK inhibitor PD0325901 to Full Medium at 2 µM final concentration (Inhibit). For the t36 and t48 time points, media was changed every 12 h from start (t0). Cells were harvested for single-cell sequencing before treatment (t0) and at 1, 4, 24, 36 and 48 h time points.

#### Generation of mutant TSC clones

For generation of CRISPR/Cas9-mediated knockout TSCs, gRNAs flanking exon(s) selected because their deletion results in frameshift mutations or a premature STOP codon were designed using the http://crispor.tefor.net/ design software and checked for high specificity by nucleotide blast searches. gRNA sequences were cloned into the Cas9.2 A.EGFP plasmid (Plasmid #48138 Addgene) (Supplementary Data 22). Transfection was carried out with Lipofectamine 2000 (ThermoFisher Scientific 11668019) reagent according to the manufacturer’s protocol. Single cell sorting was performed on a BD FACsAria cell sorter by gating the top 4-10% GFP-positive cells as putative KOs. GFP-negative cells from the same transfection were sorted as wild-type (WT) controls. KO clones were confirmed by genotyping using primers spanning the deleted exon, and by RT-qPCR with primers within the deleted exon. WT clones were confirmed by the presence of the targeted exon in genotyping PCRs. Three independent KO clones and 5 WT clones were analysed throughout, unless stated otherwise.

#### Immunofluorescence staining

Immunofluorescence staining was performed on E10.5 mouse placentas fixed in 4% paraformaldehyde (PFA) overnight and either embedded for cryosectioning in Clear Frozen Section Compound (VWR 959570838, for Embigin staining), or processed for routine paraffine histology (for CXADR, MCT1, MCT4 staining). Prior to staining, sections were deparaffinized and rehydrated, and antigen retrieval performed by boiling in 10 mM NaCitrate buffer. Blocking was performed using PBS, 0.1% Tween-20, 0.5% bovine serum albumin (PBT/BSA) for 30 min at room temperature. Primary antibody incubations were performed overnight at 4°C. Sections were washed three times in PBT/BSA, and then incubated with corresponding AlexaFluor-conjugated secondary antibodies (ThermoFisher Scientific) diluted 1:500 in PBT/BSA. Counterstaining was performed with DAPI.

For staining of TSCs, cells were plated on coverslips and fixed in ice-cold methanol for 10 min. Cells were then blocked for 30 min in PBT/BSA. Primary antibodies incubations were for 1-2 h at room temperature, followed by washes in PBT/BSA and incubation with the appropriate AlexaFluor-conjugated secondary antibodies for 1 h at room temperature. Counterstaining was performed with DAPI.

Primary antibodies and dilutions used were: CXADR 1:100 (R&D Systems AF2654), MCT1 1:100 (EMD Millipore AB1286-I), MCT4 1:100 (EMD Millipore AB3314P), Embigin 1:100 (ThermoFisher Scientific 12-5839-82), ZO1 1:200 (ThermoFisher Scientific 339100), NCAM1 1:100 (R&D AF2408-SP), STRA6 1:100 (Novus Biologicals NBP3-12353), SOX2 1:200 (R&D AF2018), KRT18 1:100 (Research Diagnostics RDI-PRO 61028), and NICOL1 1:100 (St Johns STJ196219). All dilutions were in PBT/BSA.

#### Resazurin-based cell viability assay

Cells were plated in 96-well clear, flat-bottom microplates (Corning Life Sciences 353072) at a density of 5 × 10^4^ cells per well in 100 µl culture medium and were cultured for 24 hours. Cell viability was assessed in stem cell conditions and in Remove and Inhibit conditions after 2, 4, 6, 8 or 24 hours using the Resazurin Cell Viability Kit (Cell Signaling Technology 11884S). The resazurin solution (10% of cell culture volume) were added to the plate for 24 h and cells were incubated at 37 °C. The flourescene was measured with a 560 nm excitation filter and a 615 nm emission filter in SpectraMax® ID5 microplate reader (Molecular Devices).

#### Proliferation analysis

To assess *Cxadr* KO TSCs for potential differences in proliferation rates, 3 WT and 3 KO clones were plated at a seeding density of 60,000 cells / well in a 6-well plate and assessed after 2 days and 4 days of differentiation. Cell counting was performed with an automated cell counter (Luna II, Logosbio). Unpaired t-test in GraphPad (10.0.2) was used to compare proliferation rates over time.

#### Cell fusion analysis

To assess cell fusion capacity between *Cxadr* KO and WT TSCs, membrane-specific immunofluorescent staining was performed against ZO1 on 3-day and 4-day differentiated TSCs as described above. Cells with continuous, smooth ZO1 staining were counted as single, non-syncytial cells, and those with ruffled or discontinuous membrane containing ≥2 nuclei were counted as syncytial. The syncytialisation index was determined by calculating the number of fused cells over total cell count across 5 images per clone. Results are displayed as a percentage, where error bars show standard error of means (S.E.M.) of at least three replicates. Unpaired t-test in GraphPad (10.0.2) was used for statistical analysis of significance (p < 0.05).

#### RT-qPCR expression analysis

Potential defects in TSC maintenance and differentiation capacity were investigated by analysing the expression levels and dynamics of trophoblast marker genes in mutant and control TSCs in stem cell conditions and following 2, 3 and 4 days of differentiation. Total RNA was extracted using TRI reagent (Sigma T9424), and 2 µg was used for cDNA synthesis with RevertAid H-Minus reverse transcriptase (Thermo Scientific EP0451). Quantitative (q)PCR was performed using SsoAdvanced SYBR Green Supermix (BioRad 1725274) and intron-spanning primer pairs (Supplementary Data 22) on a Bio-Rad CFX96 or CFX384 thermocycler. Normalised expression levels are displayed as mean relative to the vector control sample; error bars indicate standard error of the means (S.E.M.) of at least three replicates. ANOVA was performed in GraphPad (10.0.2) to calculate statistical significance of expression differences (p < 0.05).

### scRNA-seq Library preparation and sequencing

Trophoblast cell cultures were rinsed twice with PBS before treatment with 0.25% trypsin for 5 min at 37 °C. The enzymatic reaction was stopped using 10% FBS/PBS. Cells were collected and filtered through a 70μm strainer for t0, t1, t4 and t24 treatments. For t36 and t48 treatments the cells were not filtered. Cells were centrifuged twice to remove traces of FBS, then counted and diluted to 200,000 cells/mL in PBS/0.1% BSA (A7906-100G, Sigma).

Cell suspensions were processed as per the Drop-seq protocol described by^75^. In short: Microfluidic devices were manufactured using the Drop-seq 125um design in polydimethylsiloxane (PDMS) by replica moulding from a SU-8 master and plasma-bonded to glass slides. The device channels were rendered hydrophobic by flowing Aquapel through, flushing the excess and baking at 65 °C for 20 min. Single cells suspended in PBS/0.1% BSA and 120 primer-barcoded beads/µl (MACOSKO-2011-10, ChemGenes, Wilmington, USA) in 2x lysis buffer were loaded into 3 ml syringes. Using syringe pumps cells (4 ml/h) and beads (4 ml/h) were encapsulated in QX200™ Droplet Generation Oil for EvaGreen (#1864005, Biorad, Hertfordshire, UK) (15 ml/h) through a microfluidic device with a 125 µm channel. A magnetic stirrer was used in the bead suspension to avoid settling. The final concentration of lysis buffer achieved in the droplets was 3% Ficoll PM-400(F5415, Sigma), 0.1% Sarcosyl (L7414, Sigma), 0.01 M EDTA (AM9260G, Ambion), 0.1 M Tris pH 7.5 (15567027, Sigma) and 25 mM DTT (Sigma) which resulted in cell lysis and release of mRNA. Droplets were collected in a Falcon tube on ice for 15 min per run once stable droplet formation was achieved. After mRNA binding to the oligo-dT portion of the bead primer, the droplets were broken to pool beads for reverse transcription (EP0743, ThermoFisher). Exonuclease (EN0581, ThermoFisher) treatment removed unused primers from the beads prior to PCR amplification at up to 8,000 beads/tube ( ~ 400 cells/tube). All primer sequences used are detailed in the published Drop-Seq protocol and were purchased from IDT (Coralville, Iowa, USA).

Samples were pooled with up to 4,000 cells/sample and the DNA purified using Ampure XP beads (A63880, Beckman Coulter) before determining concentration with an Agilent HS DNA kit (5067-4626, Agilent, Stockport UK). DNA was fragmented and tagged with Illumina adapter and index sequences using Nextera Sequencing kit (FC-131-109, Illumina, Little Chesterford, UK) and 600 pg DNA/sample. Samples were diluted 1:1 in ddH_2_0 water before double-sided purification with Ampure XP using 0.6 × beads to remove large fragments and 1 × beads twice to remove contaminating primers.

Samples were analysed for size distribution and concentration using an Agilent HS DNA kit and sequenced in multiple pools across multiple lanes of Illumina Nextseq, HiSeq2500 and HiSeq4000 flow cells due to institutional upgrades (Supplementary Data 23).

For the WT and Cxadr KO scRNAseq dataset three independently derived WT and *Cxadr* KO TSC clones each, grown in either stem cell conditions or differentiated for 24 h in Inhibit or Remove conditions, were used for scRNA-seq analysis using Evercode™ WT Mini v3 single cell whole transcriptome kit (Parse Bioscience).

Cells were fixed with the Evercode Cell Fixation v3 kit (Version 1.1, Parse Bioscience) following the manufacturer’s instructions. Cells were lifted and counted with an automated cell counter (Luna II, Logosbio). Approximately 1 million cells per sample were used for fixation. Cells were pelleted, then resuspended in prefixation master mix before straining into a new tube. Cells were incubated in fixative master mix and permeabilization buffer, followed by Perm Stop buffer, cell pelleting and resuspension in cell storage buffer. Cells were strained again, counted, then frozen and stored at -80 °C until further processing.

Fixed cells were processed with a Parse Evercode WT v3 kit as per the manufacturer’s instructions. Cells were counted and loaded using the Parse Biosciences Evercode WT Sample Loading Table v2 template into barcoding plates to yield 5000 barcoded cells per biological replicate. 8 sublibraries of 12,500 cells from all samples were generated after cell barcoding. All sublibraries were pooled and sequenced in one batch on 2 lanes of NovaSeq X 25B flowcell (R1:64, R2:58, I1:8, I2:8 with 10% PhiX) to yield average 60,000 reads per cell.

### Single-cell data set analysis

The library pools were demultiplexed and individual FQ files were created for R1 and R2 in each library. Demultiplexing of raw fastq files into Nextera-indexed fastq files was performed using a custom script (dropseq_demultiplex.sh). The script contains all Nextera index name:sequence pairs to be used in the sequencing runs and calls demuxbyname.sh from BBTools (v39.05^76^).

In order to reduce read length-induced batch effects R1 and R2 for each library were trimmed. Trimming fastq files from different sequencing runs with varying read lengths was performed with a custom protocol making calls to Cutadapt (v4.0^77^), fastp (v0.23.4^78^) and BBTools (v39.05^76^). Read 1 contains the sequences barcodes, read 2 the transcript sequences. Steps performed are 1. removal of first 5 bp from Read 2; 2. adaptive trimming to high quality (Q20) runs within Read 2 reads, lower quality start and end or reads removed; 3. trimming of all Read 2 reads to a fixed length determined by the shortest read cycles used across sequencing runs (i.e., 50 bp); 4. Final step is to re-pair read 1 and read 2 fastq files so they contain the same compliment of reads and in the same order.

This improved the quality of data and increased number of genes per cell (mean nFeature_RNA = 810, median nFeature_RNA = 614.

Reads were aligned to a custom transcriptome built from bulk RNA-seq datasets for the mTSC cell line used as described in^79^.

Samples with less than 40% alignment rate were removed. Expression of all cells from each sample was added to create bulk-like RNA-seq dataset. Cumulative sample counts underwent variance stabilizing transformation (function vst from R package DESeq2^80^) and PCA was computed (prcomp function from R package stats, v3.5.3). Sample to sample correlation was calculated by using rcorr function on transformed counts (R package hmisc v4.5.0) to compute a matrix of Pearson’s r correlation coefficients for all possible pairs of columns of a matrix (Supplementary Data 25).

Libraries that were re-sequenced multiple times were merged after confirmation of lab book notes using sample correlation and number of shared UMIs (r2 > 0.85 & number of shared UMIs > 100) (Supplementary Data 25). scRNA-seq analysis was performed using package Seurat (v.3.2.0)^81^. We removed cells with fewer than 500 genes detected, leaving 136291 single cells in total. For all single-cell analysis, we performed the same initial normalization using R package sctransform (v.0.3.2^82^, that performs regularized negative binomial regression for the UMI counts. Sctransform also calculated scaled expression for downstream dimensional reduction. Dimensional reduction (PCA, UMAP) and finding neighbouring cells was performed with Seurat using the first 30 dimensions. To run UMAPs parameters these were used: reduction = “harmony”, dims = 1:30, min.dist = 0.01, n.neighbors = 25, repulsion.strength = 2, spread = 2 L.

UMAPs of gene expression were generated using the FeaturePlot function in Seurat 3.2 with colours cols=c(“grey”,“darkslateblue”, “magenta”) and order=T.

Plotting of gene expression on dot plots was done using the Seurat Function DotPlot with settings scale.max=40, dot.scale=8 and cols = c(“green”,“purple”), using SCT assay and slot data.

Marker genes were determined by Seurat FindMarkers function with P_adj_ < 0.05 and l2fc > 0.6 cutoff. Heatmaps were generated using R package ComplexHeatmap (v.1.20.0).

Average expression per cluster was calculated using the AverageExpression function in Seurat on SCT assay, slot scale.data.

#### Outlier detection

Mean expression of genes per sample was calculated for quality control and 1 sample outlier was detected on sc-to-bulk PCA (1 sample outlier), as well as single-cell PCA and UMAP. Additional outlier was identified during assessment of heterogeneity of T0 cell fraction and was removed.

#### Batch integration

Since the dataset was sequenced in batches, batch effect was investigated and identified. Several tools were compared for best performance in batch correction/integration: Seurat Integration (with/without reference), Harmony, scaling out and BUSSeq. Harmony (v.1.0)^83^ was best to correct for batch effect with settings: reduction = “pca”, assay.use = “SCT” (Supplementary Data 26).

#### Resolution

Resolution of clustering was assessed using R package clustree (v.0.4.3), separation on UMAP and number of differentially expressed genes in pairwise clusters to avoid under and overclustering. Resolution 0.8 was chosen for Together dataset as well as Inhibit only and Removal only, while resolution 0.4 was chosen for T24 and T0 only datasets. Clusters smaller than 100 cells were removed from analysis (cluster 20 in Together dataset).

#### Cell Cycle Scoring

Cells were scored for the cell cycle phase using the Cell Cycle Scoring function in Seurat.

#### Cluster Order

R Package Tempora (v.0.1.0)^30^ was used to estimate cluster order. This cluster order was used in all subsequent figures that contain cells from different time points.

#### Pseudotime

Pseudotime trajectory was calculated using R package Monocle3 (v.0.1.2)^29^ using top 3000 most variable genes, with monocle object using UMAP embedding and clusters calculated in Seurat for consistency. Function learn_graph with options: close_loop = FALSE and use_partition = FALSE was used to build trajectory. Pseudotime was then calculated using function order_cells with earliest principal node calculated with helper function form Monocle3, that selects closest vertex with highest number of cells from T0 (TSCs). Branches of pseudotime trajectories were selected by assigning earliest principal node with highest number of T0 cells as a start and end principal node of differentiated JZP or LP cluster as end, and connecting the shortest path between the points. Branches were then plotted on UMAP with cells coloured by cluster, while global overview of pseudotime was plotted on UMAP with cells coloured by pseudotime. Monocle gene expression modules were calculated with default settings. Modules of interest were selected on the basis of their co-localisation with the potential ectoplacental cone and labyrinth progenitor cells.

Gene expression in modules of interest was plotted on UMAP with the following criteria:

1. Presence in top 10 genes (by monocle score) in Remove or Inhibit dataset for modules of interest. 2. Genes present in the top 10 list for only one dataset were added to the plot of the other dataset for side-by-side comparison. 3. Known markers for labyrinth progenitor or junctional zone progenitor identity 4. Cxadr.

#### Selection of Cxadr

From markers identified in the Monocle gene expression modules associated with the clusters for JZP and LP identity we filtered the list to genes expressed in the cell surface derived using the search term uniprot-locations_(location__Cell+membrane + [SL-0039]_)-filtered---Mus_Musculus. From those genes we selected ones which had validated antibodies readily available: *Abcb1* (Thermofisher MA1-26528 and BS-0563R), *Emb* (ThermoFisher 12-5839-82), *Col13a1* (ThermoFisher PA5-62179 and PA5-101301), *Hbegf* (ThermoFisher PA5-109805 and Santa Cruz sc-365182 AF647), *Cxadr* (Sigma 05-644 and R&D systems AF2654), *Fgfbp1* (Stratech BS-1768R-A647-BSS). We found that the antibodies for EMB and CXADR (R&D systems) showed the most specific staining.

#### DEG and Term Enrichment analysis

Cluster markers were calculated for all cluster pairs using Seurat function FindMarkers with default log2 fold threshold 0.25. GO analysis and pathway enrichment was performed using enrichR R package (v.3.0)^84^ and databases: KEGG_2019_Mouse, GO_Biological_Process_2018, GO_Cellular_Component_2018, GO_Molecular_Function_2018, BioCarta_2016, WikiPathways_2019_Mouse. Heatmaps were generated using R package ComplexHeatmap (v.1.20.0)^85^. Terms for reduced heatmaps were chosen on the basis of relevance to processes related to stem cell differentiation, mitosis, transcription, translation, cell remodelling and mitochondrial respiration. In GO databases enriched pathways were often related to the same process. They were grouped by parent term using the R package rrvgo^86^ with threshold of reduction 0.7 and reduced to child terms that were enriched in the most clusters.

To understand cluster specific related pathways, for each cluster and for all cells, average normalized expression of each gene was calculated to produce scatter plots comparing each cluster with average normalized expression of all dataset. Normalized average counts were log2 transformed. A cut-off >= 1 of abs(log2 mean expression Individual Cluster - log2 mean total expression) was used to identify variable genes, and additional filtering was applied to include only genes that were identified as differentially expressed in any pairwise comparison between clusters. For each cluster, genes with elevated and lower expression levels compared to the mean expression of the total dataset were separately used for pathway enrichment (WikiPathways_2019_Mouse). For rare events when both elevated and lower expressed genes were associated with the same pathway, either elevated or lower expressed group was chosen on the basis of a lower FDR.

#### SCENIC analysis

Transcription Factor regulons and gene regulatory networks were inferred using R package SCENIC (v.1.1.2-2)^32^ and GRNBoost2 algorithm from python library Arboreto (v.0.1.6). This method (GRNBoost2 algorithm) was chosen on the basis of performance, time and memory usage and low sensitivity to the presence of dropouts^87^.

Heatmaps of regulon activity were produced using the R package Pheatmap (v.1.0.12). JZP identity was assigned by detecting clusters with high activity of Ascl2 regulons, and LP identity by Gcm1 regulon. In inhibit dataset, Cluster 3 with highest *Ascl2* regulon activity was assigned as JZP cluster, while cluster 4 with highest activity of the *Gcm1* regulon was assigned as LP cluster. Difference between JZP cluster activity and LP cluster activity was calculated for each regulon. Top 10 regulons for highest and lowest ratio, as well as top 10 regulons with highest activity in T0 cluster (cluster 9) were selected for the heatmap.

#### Single-cell RNA-seq analysis of *Cxadr* KO TSC

Fastq files from the 2 lanes were concatenated to make a single R1 and R2 file per library. Fastq files were processed using the Trailmaker data analysis platform using the mTSC custom transcriptome for the alignment step. Each sample was filtered to exclude low quality cells (UMIs/cell, Mitochondrial reads, Doublets). The dataset was split by treatment to make an Inhibit and Remove dataset. The samples in each dataset were integrated using Harmony. Seurat objects were built for Inhibit and Remove conditions and are available on Figshare along with data filtering and analysis settings. Cluster marker analysis was performed at resolution 0.7 and results tables with DEGs for each cluster are also available in Figshare.

#### Cell proportion comparisons

Cell proportion comparisons in the WT vs CXADR KO scRNA-seq dataset were performed using the *propeller* function in the package speckle (v1.2.0)^88^.

The Inhibit or Remove dataset were subsetted to the 24 h timepoint cells for the comparison. The input dataframe was created using the Seurat function FetchData, containing each cell ID, sample of origin, cluster ID and group. Propeller was run with arguments robust = T, trend = T, transform = “logit”.

### Statistics and Reproducibility

The statistical analysis of single-cell RNA-seq datasets was performed in R using the packages described in the section Single Cell Dataset Analysis. All the computational analyses were conducted using the Linux clusters at the University of Cambridge High Performance Computing Service and the Linux workstations of School of Biological Science computing.

The statistical analysis of other datasets was performed in Graphpad Prism v. 10.0.2.

Based on our extensive experience with mouse KO TSC lines and considering the necessity to perform qPCR reactions within the same microwell plate for all genes analysed, at least three independent KO clones and at least three independent wild-type clones that were derived in parallel to each KO from the same starting TSC population, were obtained from every CRISPR-Cas9 KO experiment targeting *Cxadr* and *Nicol1*. RT-qPCRs for trophoblast stem cell and differentiation markers were run in triplicate on samples from 3 to 5 time-points for three KOs, as well as the corresponding three wild-type clones that were co-derived in parallel to the KO clones from the same parental TSC population. The experiments were not randomised. Single-cell sequencing was repeated using 3-4 independent cultures of trophoblast stem cells at each time point. When KO TSCs were used 3 independent closes were used. Libraries (ie samples) were randomly assigned to sequencing batches.

In the Drop-Seq single cell analysis cells with fewer than 500 genes detected were removed.

Measurements of TSC fusion indices of wild-type and *Cxadr* KO cells were conducted in a blinded manner. For scRNA-seq and RT-qPCR data from wild-type and KO TSCs the data were not blinded as the entire methodology was based on computational quantifications and no bias could have been introduced.

### Reporting summary

Further information on research design is available in the Nature Portfolio Reporting Summary linked to this article.

## Supplementary information

Supplementary Information

Peer review File

Description of Additional Supplementary Files

Supplementary Data 1-26

Reporting Summary

## Source data

Source Data

## Data Availability

The scRNA-Seq data generated in this study have been deposited in the European Nucleotide Archive (ENA) database under the accession code: PRJEB68188. The processed Seurat R objects, Monocle and SCENIC analysis data are available at Figshare (10.6084/m9.figshare.24591180). All other data generated in this study are provided in the Supplementary Information and the Source Data files. Source data are provided with this paper.

## Code availability

Code used in this study is available at Zenodo (10.5281/zenodo.10159534).

## References

[1] Brosens, I., Pijnenborg, R., Vercruysse, L. & Romero, R. The “Great Obstetrical Syndromes” are associated with disorders of deep placentation. *Am. J. Obstet. Gynecol.* **204**, 193–201 (2011). 21094932 10.1016/j.ajog.2010.08.009 PMC3369813

[2] Smith, G. C., Pell, J. P. & Walsh, D. Pregnancy complications and maternal risk of ischaemic heart disease: a retrospective cohort study of 129 290 births. *Lancet* **357**, 2002–2006 (2001). 11438131 10.1016/S0140-6736(00)05112-6

[3] Barker, D. J., Bull, A. R., Osmond, C. & Simmonds, S. J. Fetal and placental size and risk of hypertension in adult life. *Br. Méd. J.* **301**, 259 (1990). 2390618 10.1136/bmj.301.6746.259 PMC1663477

[4] Petrou, S., Sach, T. & Davidson, L. The long‐term costs of preterm birth and low birth weight: results of a systematic review. *Child.: Care, Heal. Dev.* **27**, 97–115 (2001). 10.1046/j.1365-2214.2001.00203.x 11251610

[5] Simmons, D. G., Fortier, A. L. & Cross, J. C. Diverse subtypes and developmental origins of trophoblast giant cells in the mouse placenta. *Dev. Biol.* **304**, 567–578 (2007). 17289015 10.1016/j.ydbio.2007.01.009

[6] Marsh, B. & Blelloch, R. Single nuclei RNA-seq of mouse placental labyrinth development. *Elife* **9**, (2020). 10.7554/eLife.60266 PMC7669270 33141023

[7] Jiang, X. et al. A differentiation roadmap of murine placentation at single-cell resolution. *Cell Discov.* **9**, 30 (2023). 36928215 10.1038/s41421-022-00513-z PMC10020559

[8] Tanaka, S., Kunath, T., Hadjantonakis, A.-K., Nagy, A. & Rossant, J. Promotion of Trophoblast Stem Cell Proliferation by FGF4. *Science* **282**, 2072–2075 (1998). 9851926 10.1126/science.282.5396.2072

[9] Okae, H. et al. Derivation of Human Trophoblast Stem Cells. *Cell Stem Cell* **22**, 50–63.e6 (2018). 29249463 10.1016/j.stem.2017.11.004

[10] Turco, M. Y. et al. Trophoblast organoids as a model for maternal–fetal interactions during human placentation. *Nature* **564**, 263–267 (2018). 30487605 10.1038/s41586-018-0753-3 PMC7220805

[11] Haider, S. et al. Self-Renewing Trophoblast Organoids Recapitulate the Developmental Program of the Early Human Placenta. *Stem Cell Rep.* **11**, 537–551 (2018). 10.1016/j.stemcr.2018.07.004 PMC6092984 30078556

[12] Rossant, J. & Cross, J. C. Placental development: lessons from mouse mutants. *Nat. Rev. Genet* **2**, 538–548 (2001). 11433360 10.1038/35080570

[13] Hemberger, M., Hanna, C. W. & Dean, W. Mechanisms of early placental development in mouse and humans. *Nat Rev Genet* 1–17 10.1038/s41576-019-0169-4 (2019). 10.1038/s41576-019-0169-4 31534202

[14] Hemberger, M. & Dean, W. The placenta: epigenetic insights into trophoblast developmental models of a generation-bridging organ with long-lasting impact on lifelong health. *Physiol. Rev.* **103**, 2523–2560 (2023). 37171808 10.1152/physrev.00001.2023

[15] Watson, E. D. & Cross, J. C. Development of Structures and Transport Functions in the Mouse Placenta. *Physiology* **20**, 180–193 (2005). 15888575 10.1152/physiol.00001.2005

[16] Erlebacher, A., Price, K. A. & Glimcher, L. H. Maintenance of mouse trophoblast stem cell proliferation by TGF-β/activin. *Dev. Biol.* **275**, 158–169 (2004). 15464579 10.1016/j.ydbio.2004.07.032

[17] Kubaczka, C. et al. Derivation and Maintenance of Murine Trophoblast Stem Cells under Defined Conditions. *Stem Cell Rep.* **2**, 232–242 (2014). 10.1016/j.stemcr.2013.12.013 PMC3923226 24527396

[18] Latos, P. A. et al. Fgf and Esrrb integrate epigenetic and transcriptional networks that regulate self-renewal of trophoblast stem cells. *Nat. Commun.* **6**, 7776 (2015). 26206133 10.1038/ncomms8776 PMC4525203

[19] Adachi, K. et al. Context-Dependent Wiring of Sox2 Regulatory Networks for Self-Renewal of Embryonic and Trophoblast Stem Cells. *Mol. Cell* **52**, 380–392 (2013). 24120664 10.1016/j.molcel.2013.09.002

[20] Natale, D. R. C., Hemberger, M., Hughes, M. & Cross, J. C. Activin promotes differentiation of cultured mouse trophoblast stem cells towards a labyrinth cell fate. *Dev. Biol.* **335**, 120–131 (2009). 19716815 10.1016/j.ydbio.2009.08.022

[21] Latos, P. A. et al. Elf5-centered transcription factor hub controls trophoblast stem cell self-renewal and differentiation through stoichiometry-sensitive shifts in target gene networks. *Genes Dev.* **29**, 2435–2448 (2015). 26584622 10.1101/gad.268821.115 PMC4691948

[22] Motomura, K. et al. Cellular Dynamics of Mouse Trophoblast Stem Cells: Identification of a Persistent Stem Cell Type1. *Biol. Reprod.* **94**, 1–14 (2016). 122. 10.1095/biolreprod.115.137125 PMC6702784 27122635

[23] Chun, J., Han, Y. & Ahn, K. Psx homeobox gene is X‐linked and specifically expressed in trophoblast cells of mouse placenta. *Dev. Dyn.* **216**, 257–266 (1999). 10590477 10.1002/(SICI)1097-0177(199911)216:3<257::AID-DVDY4>3.0.CO;2-0

[24] MacLean, J. A. et al. Rhox: A New Homeobox Gene Cluster. *Cell* **120**, 369–382 (2005). 15707895 10.1016/j.cell.2004.12.022

[25] Hayashi, K. et al. Dynamic Equilibrium and Heterogeneity of Mouse Pluripotent Stem Cells with Distinct Functional and Epigenetic States. *Cell Stem Cell* **3**, 391–401 (2008). 18940731 10.1016/j.stem.2008.07.027 PMC3847852

[26] Ray, S. et al. Hippo signaling cofactor, WWTR1, at the crossroads of human trophoblast progenitor self-renewal and differentiation. *Proc. Natl Acad. Sci. USA* **119**, e2204069119 (2022). 36037374 10.1073/pnas.2204069119 PMC9457323

[27] Streef, T. J. et al. Single-cell analysis of human fetal epicardium reveals its cellular composition and identifies CRIP1 as a modulator of EMT. *Stem Cell Rep.* **18**, 1421–1435 (2023). 10.1016/j.stemcr.2023.06.002 PMC10362506 37390825

[28] Kiyozumi, D. et al. A small secreted protein NICOL regulates lumicrine-mediated sperm maturation and male fertility. *Nat. Commun.* **14**, 2354 (2023). 37095084 10.1038/s41467-023-37984-x PMC10125973

[29] Cao, J. et al. The single-cell transcriptional landscape of mammalian organogenesis. *Nature* **566**, 496–502 (2019). 30787437 10.1038/s41586-019-0969-x PMC6434952

[30] Tran, T. N. & Bader, G. D. Tempora: Cell trajectory inference using time-series single-cell RNA sequencing data. *PLoS Comput. Biol.* **16**, e1008205 (2020). 32903255 10.1371/journal.pcbi.1008205 PMC7505465

[31] Latos, P. A. & Hemberger, M. From the stem of the placental tree: trophoblast stem cells and their progeny. *Development* **143**, 3650–3660 (2016). 27802134 10.1242/dev.133462

[32] Aibar, S. et al. SCENIC: single-cell regulatory network inference and clustering. *Nat. Methods* **14**, 1083–1086 (2017). 28991892 10.1038/nmeth.4463 PMC5937676

[33] Van de Sande, B. et al. A scalable SCENIC workflow for single-cell gene regulatory network analysis. *Nat. Protoc.* **15**, 2247–2276 (2020). 32561888 10.1038/s41596-020-0336-2

[34] Ng, R. K. et al. Epigenetic restriction of embryonic cell lineage fate by methylation of Elf5. *Nat. Cell Biol.* **10**, 1280–1290 (2008). 18836439 10.1038/ncb1786 PMC2635539

[35] Strumpf, D. et al. Cdx2 is required for correct cell fate specification and differentiation of trophectoderm in the mouse blastocyst. *Development* **132**, 2093–2102 (2005). 15788452 10.1242/dev.01801

[36] Russ, A. P. et al. Eomesodermin is required for mouse trophoblast development and mesoderm formation. *Nature* **404**, 95–99 (2000). 10716450 10.1038/35003601

[37] Ji, H. et al. Cell-Type Independent MYC Target Genes Reveal a Primordial Signature Involved in Biomass Accumulation. *Plos One* **6**, e26057 (2011). 22039435 10.1371/journal.pone.0026057 PMC3198433

[38] Rhodes, J. M. et al. Positive regulation of c-Myc by cohesin is direct, and evolutionarily conserved. *Dev. Biol.* **344**, 637–649 (2010). 20553708 10.1016/j.ydbio.2010.05.493 PMC2941799

[39] Guan, F. H. X. et al. The antiproliferative ELF2 isoform, ELF2B, induces apoptosis in vitro and perturbs early lymphocytic development in vivo. *J. Hematol. Oncol.* **10**, 75 (2017). 28351373 10.1186/s13045-017-0446-7 PMC5371273

[40] Vaiman, D., Calicchio, R. & Miralles, F. Landscape of Transcriptional Deregulations in the Preeclamptic Placenta. *Plos One* **8**, e65498 (2013). 23785430 10.1371/journal.pone.0065498 PMC3681798

[41] Minamide, K. et al. IRF2 maintains the stemness of colonic stem cells by limiting physiological stress from interferon. *Sci. Rep.-uk* **10**, 14639 (2020). 10.1038/s41598-020-71633-3 PMC7479133 32901054

[42] Iliopoulos, D. et al. Loss of miR-200 Inhibition of Suz12 Leads to Polycomb-Mediated Repression Required for the Formation and Maintenance of Cancer Stem Cells. *Mol. Cell* **39**, 761–772 (2010). 20832727 10.1016/j.molcel.2010.08.013 PMC2938080

[43] Hemberger, M. et al. UniGene cDNA array-based monitoring of transcriptome changes during mouse placental development. *Proc. Natl Acad. Sci. U. S. Am.* **98**, 13126–13131 (2001). 10.1073/pnas.231396598 PMC60835 11698681

[44] Ballasy, N. N. et al. Padi2/3 Deficiency Alters the Epigenomic Landscape and Causes Premature Differentiation of Mouse Trophoblast Stem Cells. *Cells* **11**, 2466 (2022). 36010543 10.3390/cells11162466 PMC9406452

[45] Adamson, S. L. et al. Interactions between trophoblast cells and the maternal and fetal circulation in the mouse placenta. *Dev. Biol.* **250**, 358–373 (2002). 12376109 10.1016/s0012-1606(02)90773-6

[46] Rivron, N. C. et al. Blastocyst-like structures generated solely from stem cells. *Nature* **557**, 106–111 (2018). 29720634 10.1038/s41586-018-0051-0

[47] Parast, M. M. et al. PPARγ Regulates Trophoblast Proliferation and Promotes Labyrinthine Trilineage Differentiation. *PLoS ONE* **4**, e8055 (2009). 19956639 10.1371/journal.pone.0008055 PMC2778869

[48] Anson-Cartwright, L. et al. The glial cells missing-1 protein is essential for branching morphogenesis in the chorioallantoic placenta. *Nat. Genet.* **25**, 311–314 (2000). 10888880 10.1038/77076

[49] Zhu, D., Gong, X., Miao, L., Fang, J. & Zhang, J. Efficient Induction of Syncytiotrophoblast Layer II Cells from Trophoblast Stem Cells by Canonical Wnt Signaling Activation. *Stem Cell Rep.* **9**, 2034–2049 (2017). 10.1016/j.stemcr.2017.10.014 PMC5785677 29153986

[50] Radford, B. N. et al. Defects in placental syncytiotrophoblast cells are a common cause of developmental heart disease. *Nat. Commun.* **14**, 1174 (2023). 36859534 10.1038/s41467-023-36740-5 PMC9978031

[51] Simmons, D. G. et al. Early patterning of the chorion leads to the trilaminar trophoblast cell structure in the placental labyrinth. *Development* **135**, 2083–2091 (2008). 18448564 10.1242/dev.020099 PMC3159581

[52] Nadeau, V. & Charron, J. Essential role of the ERK/MAPK pathway in blood-placental barrier formation. *Development* **141**, 2825–2837 (2014). 24948605 10.1242/dev.107409

[53] Outhwaite, J. E., Patel, J. & Simmons, D. G. Secondary Placental Defects in Cxadr Mutant Mice. *Front. Physiol.* **10**, 622 (2019). 31338035 10.3389/fphys.2019.00622 PMC6628872

[54] Murray, A., Sienerth, A. R. & Hemberger, M. Plet1 is an epigenetically regulated cell surface protein that provides essential cues to direct trophoblast stem cell differentiation. *Sci. Rep.-uk* **6**, 25112 (2016). 10.1038/srep25112 PMC4848516 27121762

[55] Perez-Garcia, V. et al. Placentation defects are highly prevalent in embryonic lethal mouse mutants. *Nature* **555**, 463–468 (2018). 29539633 10.1038/nature26002 PMC5866719

[56] Ouseph, M. M. et al. Atypical E2F repressors and activators coordinate placental development. *Dev. Cell* **22**, 849–862 (2012). 22516201 10.1016/j.devcel.2012.01.013 PMC3483796

[57] Weijts, B. G. M. W. et al. E2F7 and E2F8 promote angiogenesis through transcriptional activation of VEGFA in cooperation with HIF1. *Embo J.* **31**, 3871–3884 (2012). 22903062 10.1038/emboj.2012.231 PMC3463843

[58] Mizuno, M. et al. The role of E2F8 in the human placenta. *Mol. Med Rep.* **19**, 293–301 (2019). 30387815 10.3892/mmr.2018.9617 PMC6297733

[59] Zhang, C. et al. Proteolysis of methylated SOX2 protein is regulated by L3MBTL3 and CRL4DCAF5 ubiquitin ligase. *J. Biol. Chem.* **294**, 476–489 (2019). 30442713 10.1074/jbc.RA118.005336 PMC6333883

[60] Dhaliwal, N. K., Abatti, L. E. & Mitchell, J. A. KLF4 protein stability regulated by interaction with pluripotency transcription factors overrides transcriptional control. *Genes Dev.* **33**, 1069–1082 (2019). 31221664 10.1101/gad.324319.119 PMC6672055

[61] Uy, G. D., Downs, K. M. & Gardner, R. L. Inhibition of trophoblast stem cell potential in chorionic ectoderm coincides with occlusion of the ectoplacental cavity in the mouse. *Development* **129**, 3913–3924 (2002). 12135928 10.1242/dev.129.16.3913

[62] Hayashi, S., Lewis, P., Pevny, L. & McMahon, A. P. Efficient gene modulation in mouse epiblast using a Sox2Cre transgenic mouse strain. *Mech. Dev.* **119**, S97–S101 (2002). 14516668 10.1016/s0925-4773(03)00099-6

[63] Wenzel, P. L. & Leone, G. Expression of Cre recombinase in early diploid trophoblast cells of the mouse placenta. *genesis* **45**, 129–134 (2007). 17299749 10.1002/dvg.20276

[64] Copp, A. J. Death before birth: clues from gene knockouts and mutations. *Trends Genet* **11**, 87–93 (1995). 7732578 10.1016/S0168-9525(00)89008-3

[65] Endele, S., Nelkenbrecher, C., Bördlein, A., Schlickum, S. & Winterpacht, A. C4ORF48, a gene from the Wolf-Hirschhorn syndrome critical region, encodes a putative neuropeptide and is expressed during neocortex and cerebellar development. *neurogenetics* **12**, 155–163 (2011). 21287218 10.1007/s10048-011-0275-8

[66] Yang, J. et al. The secreted micropeptide C4orf48 enhances renal fibrosis via an RNA-binding mechanism. *J. Clin. Investig.* **134**, e178392 (2024). 38625739 10.1172/JCI178392 PMC11093611

[67] Marsman, R. F. J. et al. Coxsackie and Adenovirus Receptor Is a Modifier of Cardiac Conduction and Arrhythmia Vulnerability in the Setting of Myocardial Ischemia. *J. Am. Coll. Cardiol.* **63**, 549–559 (2014). 24291282 10.1016/j.jacc.2013.10.062 PMC3926969

[68] Yuen, S., Smith, J., Caruso, L., Balan, M. & Opavsky, M. A. The coxsackie–adenovirus receptor induces an inflammatory cardiomyopathy independent of viral infection. *J. Mol. Cell. Cardiol.* **50**, 826–840 (2011). 21352828 10.1016/j.yjmcc.2011.02.011

[69] Fechner, H. et al. Induction of Coxsackievirus-Adenovirus–Receptor Expression During Myocardial Tissue Formation and Remodeling. *Circulation* **107**, 876–882 (2003). 12591759 10.1161/01.cir.0000050150.27478.c5

[70] Sharma, V., Perry, D. J. & Eghtesady, P. Role of coxsackie‐adenovirus receptor in cardiac development and pathogenesis of congenital heart disease. *Birth Defects Res* **113**, 535–545 (2021). 33369284 10.1002/bdr2.1860

[71] Koi, H. et al. Differential Expression of the Coxsackievirus and Adenovirus Receptor Regulates Adenovirus Infection of the Placenta1. *Biol. Reprod.* **64**, 1001–1009 (2001). 11207218 10.1095/biolreprod64.3.1001

[72] Krivega, M., Geens, M. & Van de Velde, H. CAR expression in human embryos and hESC illustrates its role in pluripotency and tight junctions. *Reproduction* **148**, 531–544 (2014). 25118298 10.1530/REP-14-0253

[73] Fitzgerald, B. et al. Villous trophoblast abnormalities in extremely preterm deliveries with elevated second trimester maternal serum hCG or inhibin-A. *Placenta* **32**, 339–345 (2011). 21388678 10.1016/j.placenta.2011.01.018

[74] Hoffman, M. K. The great obstetrical syndromes and the placenta. *BJOG: Int. J. Obstet. Gynaecol.* **130**, 8–15 (2023). 10.1111/1471-0528.17613 PMC10834844 37530495

[75] Macosko, E. Z. et al. Highly Parallel Genome-wide Expression Profiling of Individual Cells Using Nanoliter Droplets. *Cell* **161**, 1202–1214 (2015). 26000488 10.1016/j.cell.2015.05.002 PMC4481139

[76] Bushnell, B. BBMap. https://sourceforge.net/projects/bbmap/ (2014).

[77] Martin, M. Cutadapt removes adapter sequences from high-throughput sequencing reads. *EMBnetJ* **17**, 10–12 (2011).

[78] Chen, S. Ultrafast one‐pass FASTQ data preprocessing, quality control, and deduplication using fastp. *iMeta* **2**, (2023). 10.1002/imt2.107 PMC10989850 38868435

[79] Pertea, M., Kim, D., Pertea, G. M., Leek, J. T. & Salzberg, S. L. Transcript-level expression analysis of RNA-seq experiments with HISAT, StringTie and Ballgown. *Nat. Protoc.* **11**, 1650–1667 (2016). 27560171 10.1038/nprot.2016.095 PMC5032908

[80] Love, M. I., Huber, W. & Anders, S. Moderated estimation of fold change and dispersion for RNA-seq data with DESeq2. *Genome Biol.* **15**, 550 (2014). 25516281 10.1186/s13059-014-0550-8 PMC4302049

[81] Stuart, T. et al. Comprehensive Integration of Single-Cell Data. *Cell* **177**, 1888–1902.e21 (2019). 31178118 10.1016/j.cell.2019.05.031 PMC6687398

[82] Hafemeister, C. & Satija, R. Normalization and variance stabilization of single-cell RNA-seq data using regularized negative binomial regression. *Genome Biol.* **20**, 296 (2019). 31870423 10.1186/s13059-019-1874-1 PMC6927181

[83] Korsunsky, I. et al. Fast, sensitive and accurate integration of single-cell data with Harmony. *Nat. Methods* **16**, 1289–1296 (2019). 31740819 10.1038/s41592-019-0619-0 PMC6884693

[84] Chen, E. Y. et al. Enrichr: interactive and collaborative HTML5 gene list enrichment analysis tool. *BMC Bioinform* **14**, 128 (2013). 10.1186/1471-2105-14-128 PMC3637064 23586463

[85] Gu, Z., Eils, R. & Schlesner, M. Complex heatmaps reveal patterns and correlations in multidimensional genomic data. *Bioinformatics* **32**, 2847–2849 (2016). 27207943 10.1093/bioinformatics/btw313

[86] Sayols, S. rrvgo: a Bioconductor package for interpreting lists of Gene Ontology terms. *microPublication Biol*. **2023**, 10.17912/micropub.biology.000811 (2023). 10.17912/micropub.biology.000811 PMC10155054 37151216

[87] Pratapa, A., Jalihal, A. P., Law, J. N., Bharadwaj, A. & Murali, T. M. Benchmarking algorithms for gene regulatory network inference from single-cell transcriptomic data. *Nat. Methods* **17**, 147–154 (2020). 31907445 10.1038/s41592-019-0690-6 PMC7098173

[88] Phipson, B. et al. propeller: testing for differences in cell type proportions in single cell data. *Bioinformatics* **38**, 4720–4726 (2022). 36005887 10.1093/bioinformatics/btac582 PMC9563678

