# Supplementary material for: Single-cell RNA sequencing identifies CXADR as a fate determinant of the placental exchange surface
Source: Nat Commun. 2025 Jan 2;16:142. doi: 10.1038/s41467-024-55597-w (PMC11695997; doi:10.1038/s41467-024-55597-w)
Supplement: Supplementary file 1 — Supplementary Information [file 41467_2024_55597_MOESM1_ESM.pdf]

Single-cell RNA sequencing identifies CXADR as a fate determinant of the placental exchange surface

Supplementary Figures

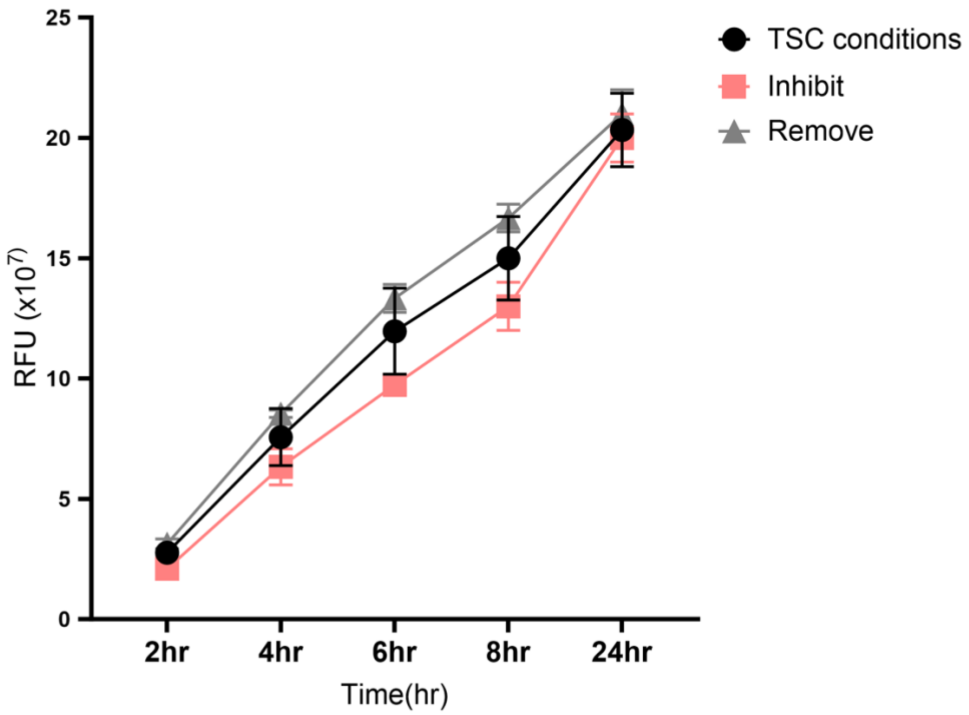

Supplementary Figure 1. TSC viability assessment in Inhibit and Remove conditions.

Assessment of the viability of TSCs grown in stem cell conditions and upon differentiation in Inhibit (i.e., exposure to MEKi) and Remove conditions. RFU = Relative Fluorescence Units.

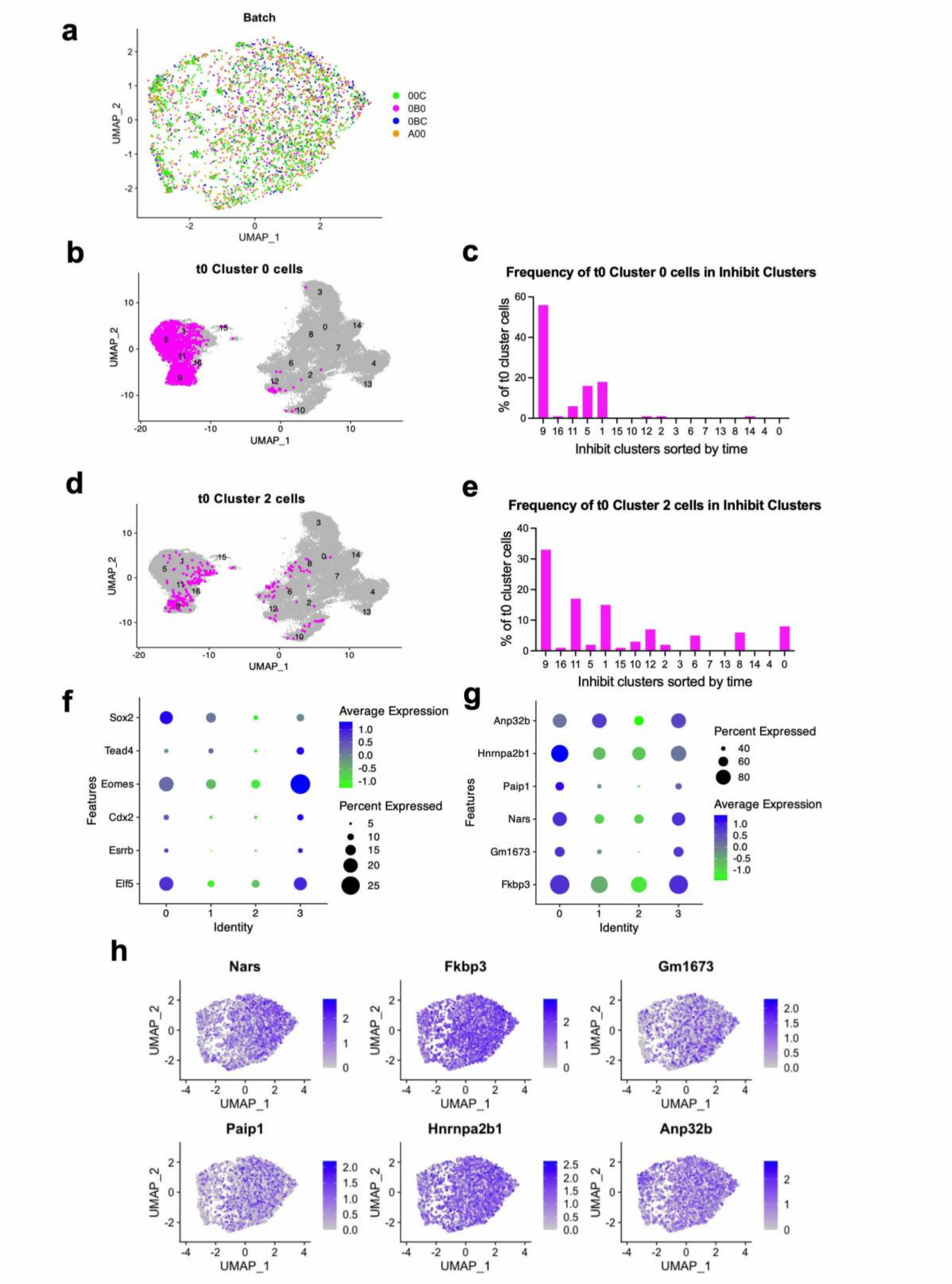

Supplementary Figure 2. Gene expression in t0 dataset.

(**a**) UMAP of t0 dataset coloured by batch – only the analysis batches that contained t0 cells are included in this analysis. Dimensional reduction of the dataset using UMAP (uniform manifold approximation and projection, calculated in Seurat) after Harmony integration at resolution 0.4. Cells on the UMAP were coloured by analysis batch (0BC – blue, 00C – green, 0B0 -red and A00 – black). (**b**) t0 Cluster 0 cells plotted on UMAP plot of Inhibit dataset. (**c**) Frequency of t0 cluster 0

cells in full Inhibit time course. The sum of cells in clusters 10, 12, 2, 3, 6, 7, 13, 8, 14, 4, and 0 was used to calculate the total % of t0 cells clustering together with t24-28 cells. (**d**) t0 cluster 2 cells plotted on UMAP plot of Inhibit dataset. (**e**) Frequency of t0 cluster 2 cells in full Inhibit time course. The sum of cells in clusters 10, 12, 2, 3, 6, 7, 13, 8, 14, 4, and 0 was used to calculate the total % of t0 cluster 2 cells clustering together with t24-28 cells. (**f-g**) DotPlot of expression of mTSC marker genes (**f**) and cluster c0 vs c2 DEGs (**g**) in t0 clusters. The size of the dots represents number of cells that express the marker. The colour of dots represents the average expression of the marker in each cluster, scaled for the dataset. All values were mean centred for each gene. (**h**) UMAP plot of Inhibit dataset with cells coloured by gene expression levels.

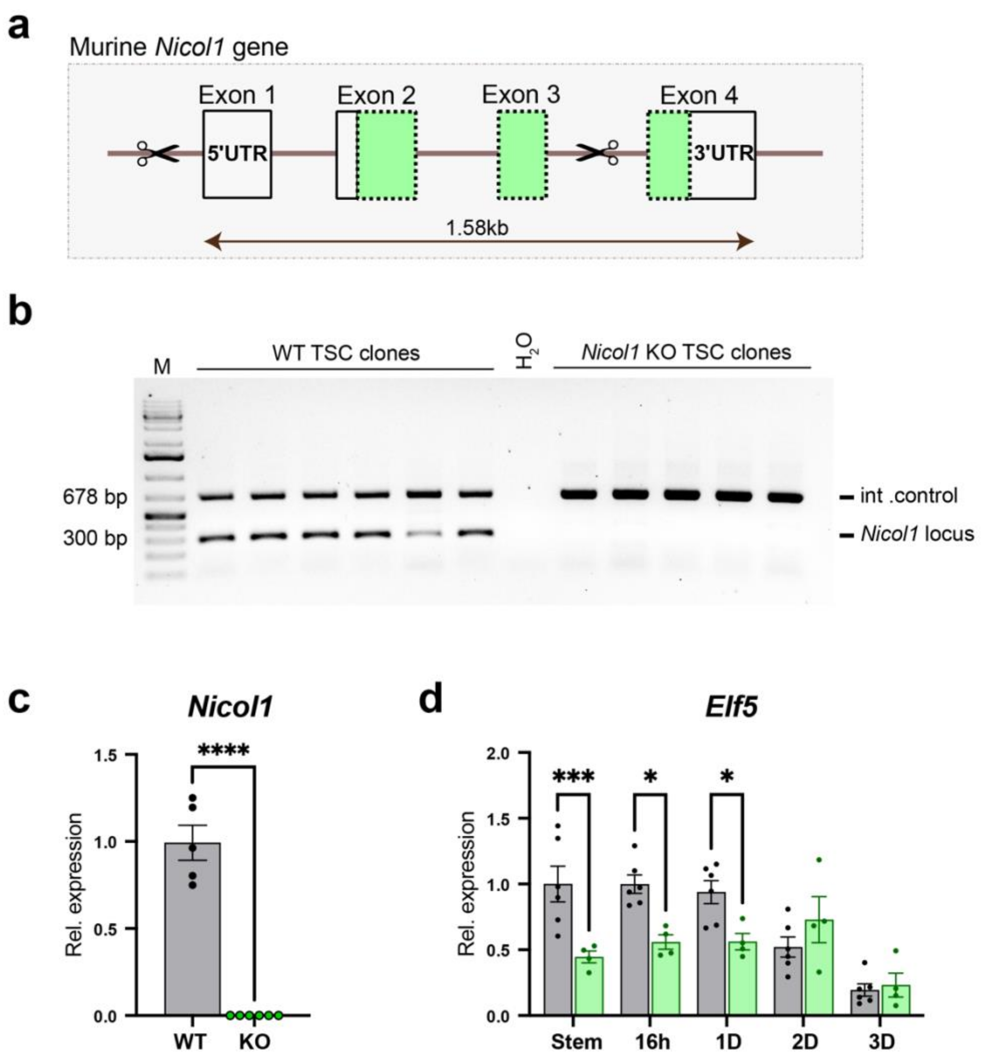

Supplementary Figure 3. Generation of *Nicol1* KO TSCs.

(**a**) Diagram of *Nicol1* gene locus and the guide RNAs designed to knock out the gene. (**b**) Genotyping PCR showing absence of targeted *Nicol1* exons. (**c**) RT-qPCR for *Nicol1* gene expression in wild-type (WT, n=5) and *Nicol1* KO (n=6) TSCs. Data are normalized to WT cells in stem cell conditions and are depicted as mean +/- SEM. Statistical significance was calculated using two-sided unpaired t-test. **** p<0.0001. (**d**) RT-qPCR for stem cell marker *Elf5* in WT (n=6 independently derived TSC clones) and *Nicol1* KO (n=4 independently derived TSC clones) TSCs grown in stem cell conditions and after the indicated time points of differentiation in Remove conditions. Data are normalized to WT cells in stem cell conditions and are depicted as mean +/- SEM. Statistical significance was calculated using 2-way ANOVA. * p<0.05, *** p<0.001.

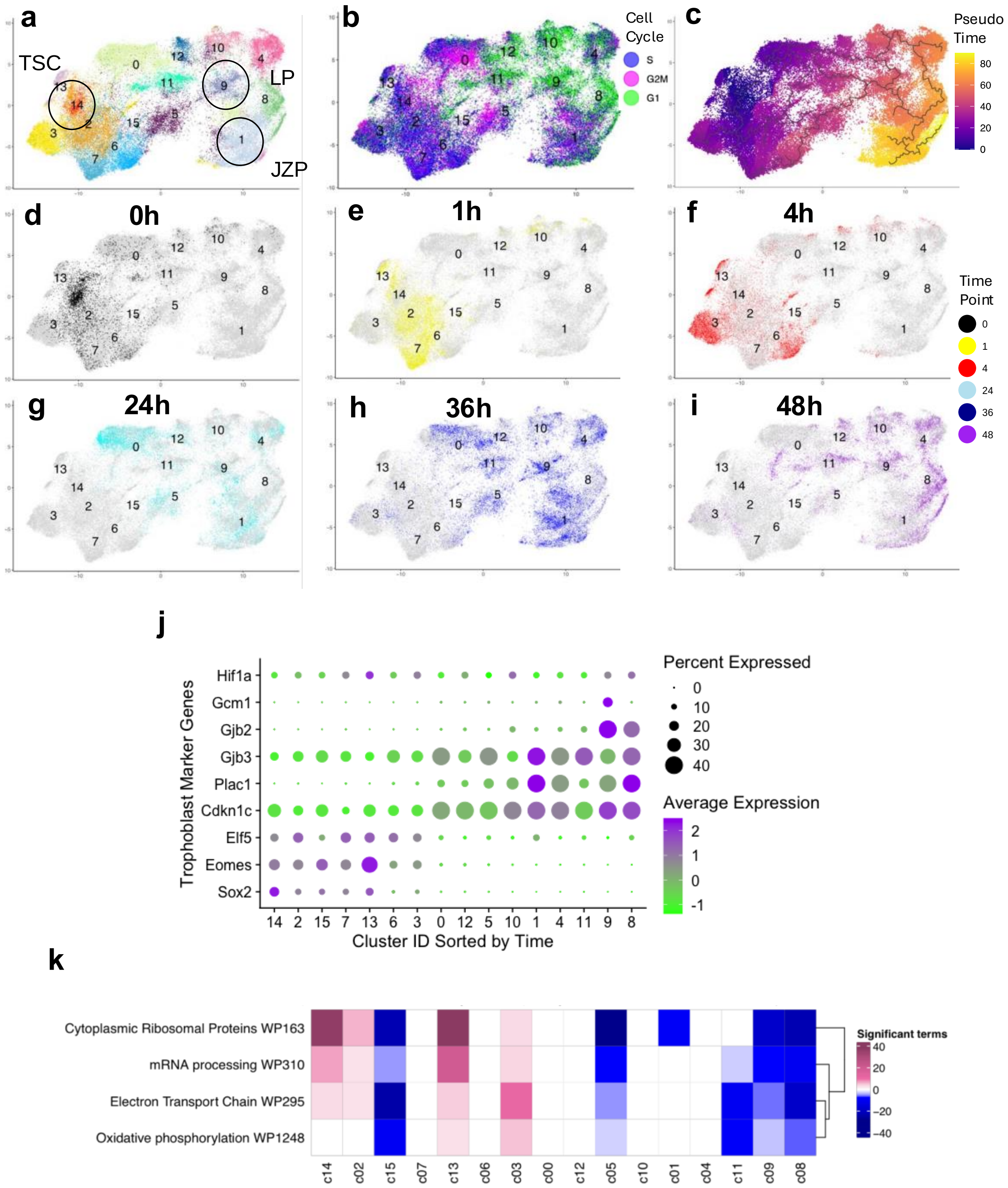

Supplementary Figure 4. Clustering of Remove dataset.
(**a-i**) UMAP grid of the Harmony-integrated Remove TSC differentiation dataset
Dimensional reduction of the dataset using UMAP after harmony integration. **a**) UMAP coloured by cluster, resolution = 0.8 (**b**) UMAP coloured by assigned cell cycle phase using the Seurat function CellCycleScoring, (**c**) UMAP coloured by Pseudotime using Monocle3 **d**) UMAP with only t0 cells coloured, (**e-i**) UMAPs with cells coloured at timepoints: 1h (**e**), 4h (**f**), 24h (**g**), 36h (**h**) and 48h (**i**). (**j**) Trophoblast markers over the course of TSC differentiation in the Harmony-integrated Remove dataset. Markers were selected from literature and grouped by differentiation trajectory. The size of the dots represents number of cells that express the marker. The colour of dots represents the average expression of the marker in each cluster, scaled for the dataset. All values were mean centred for each gene. Clusters were ordered by time using the R package Tempora with

undifferentiated cells on the bottom. (**k**) Heatmap of gene expression in top WikiPathways in the Inhibit TSC differentiation dataset. Pink colour represents enrichment of pathway in specific cluster compared to other clusters, blue colour represents pathways that are underrepresented in specific cluster compared to other clusters. Cluster specific variable genes, identified by comparing average normalised expression for each gene on scatterplots (see Methods for details), were used to calculate pathway enrichment for individual clusters.

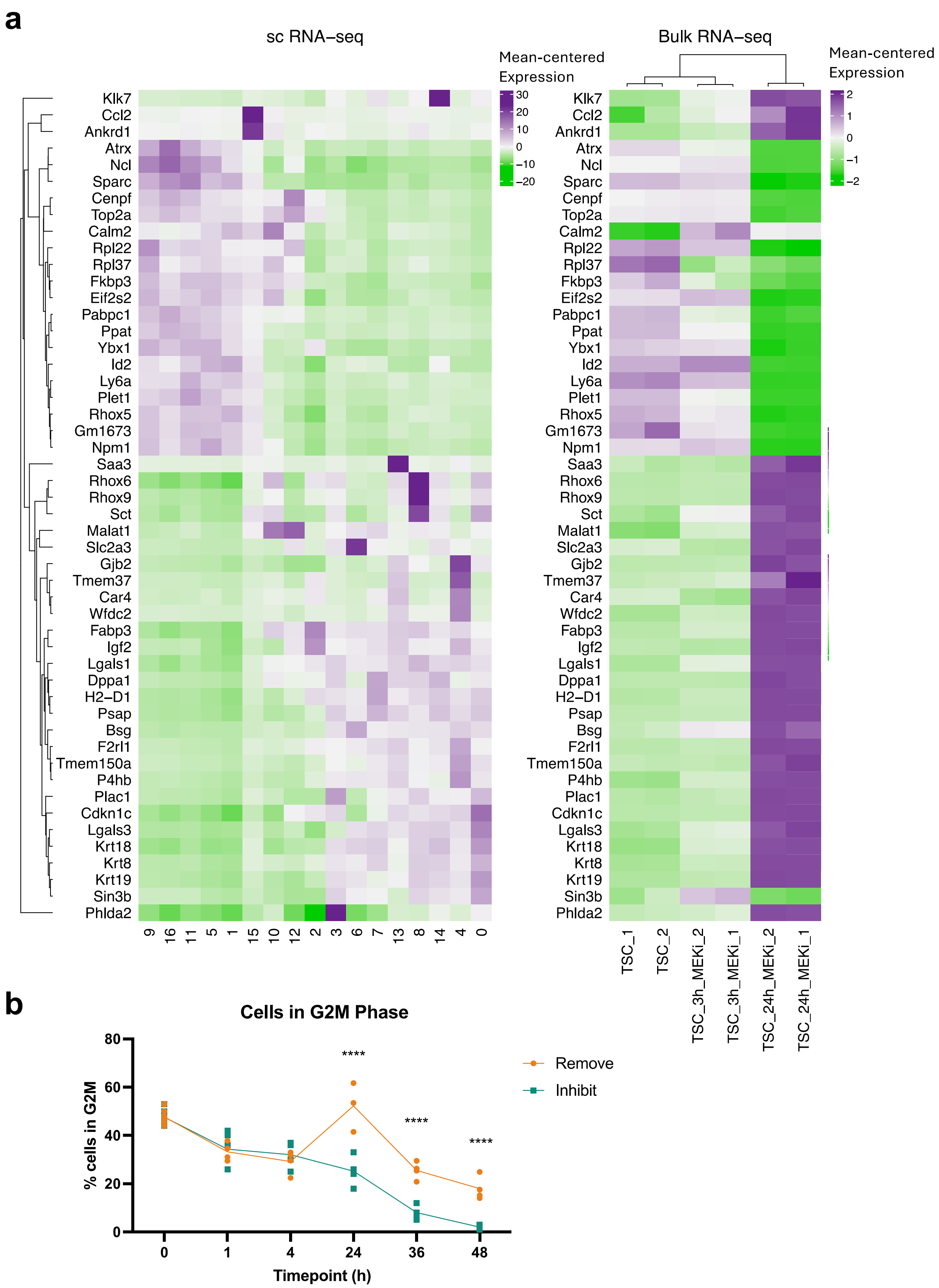

Supplementary Figure 5. Heatmap of Inhibit single cell data and bulk RNAseq ordered by time. (**a**) Heatmap showing expression of top 50 most variable genes in the Inhibit dataset and in a bulk RNA-seq dataset from the same TSCs treated with MEK inhibitor at 0h, 3h and 24h. Genes were

selected using the Seurat function VariableGenes. Genes were hierarchically clustered and clusters were ordered by time based on Tempora. Colour represents average expression of the marker gene in a cluster. (**b**) Percentage of cells in G2M phase as scored by Seurat in Inhibit and Remove datasets at each time point. Data plotted are percentage for each biological replicate. 24h p(adj) = 0.0000000014; 36h p(adj) = 0.0000036286; 48h p(adj) = 0.0000181688 (**** $p<0.0001$ in a 2-way ANOVA with Tukey post-hoc test).

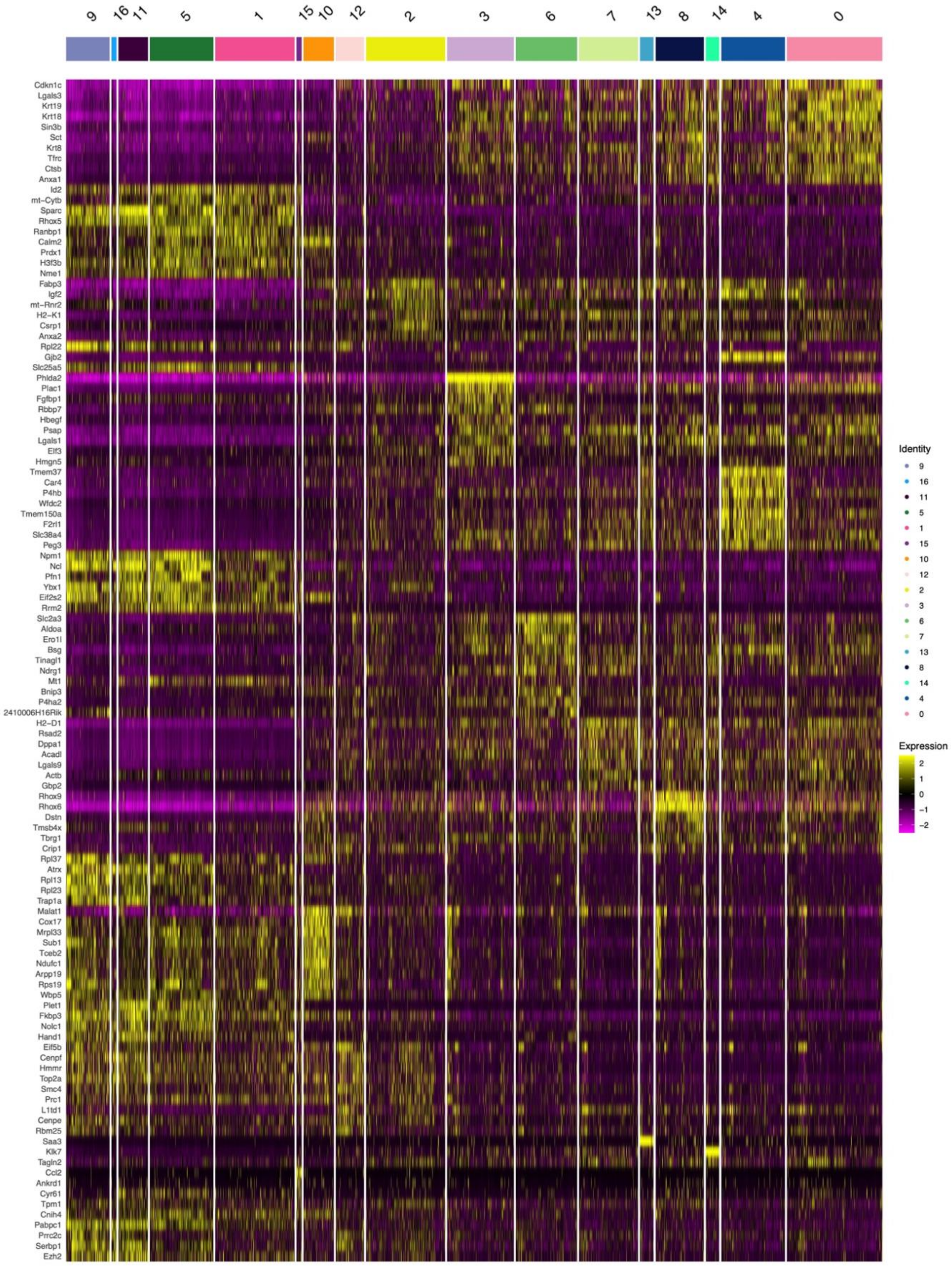

Supplementary Figure 6. Heatmap showing expression of top markers for Inhibit dataset.

Top 10 markers per cluster were selected on the basis of highest absolute fold-change. Genes were hierarchically clustered and clusters arranged in the temporal order identified by Tempora. Colour represents average expression of marker gene in a cluster. Supplementary tables 7 and 8 list all markers detected using function: FindAllMarkers.

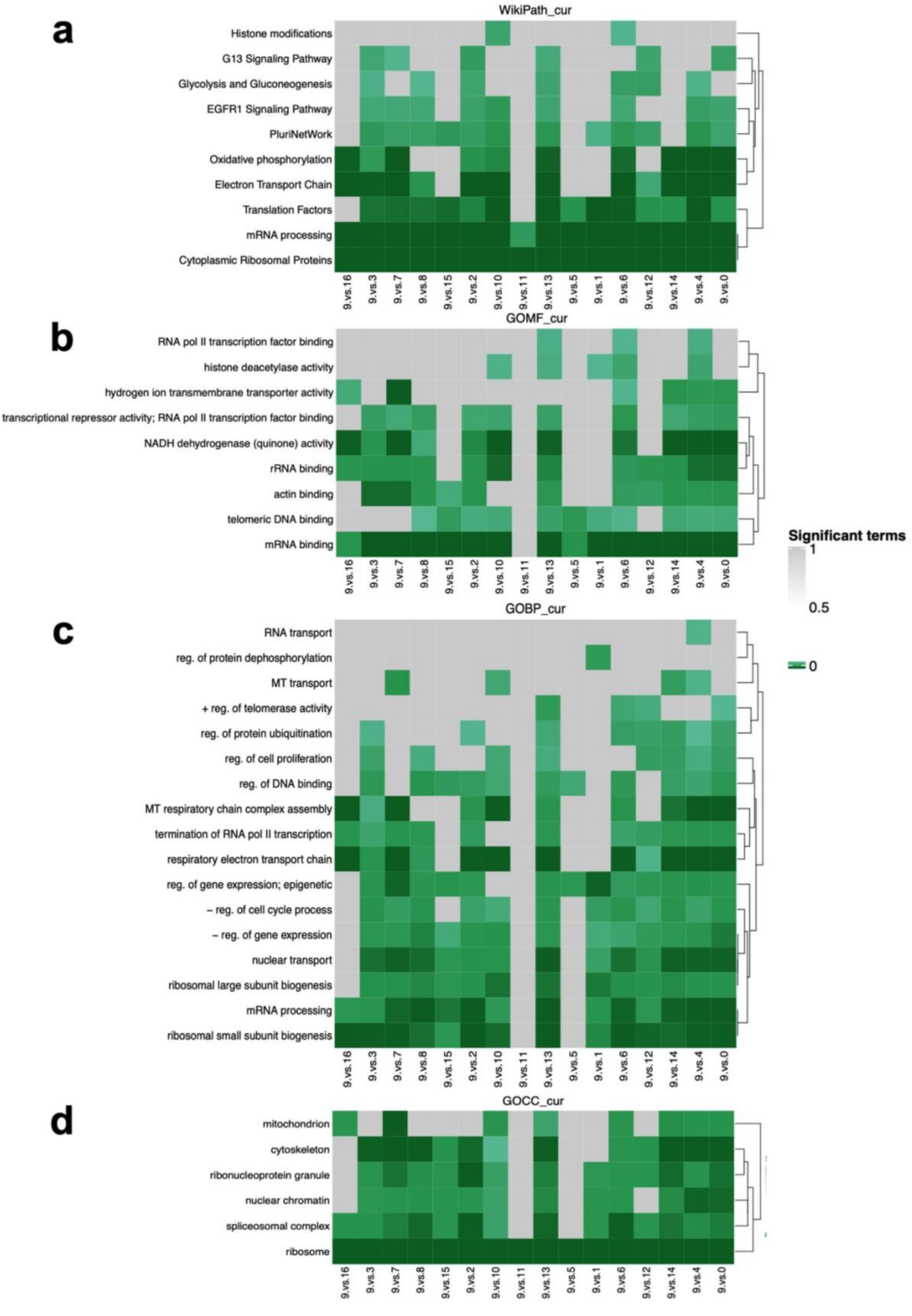

Supplementary Figure 7. Enrichment analysis for Harmony-integrated Inhibit TSC differentiation dataset.
Heatmaps of selected terms from pathway enrichment using databases: (**a**) "WikiPathways_2019_Mouse", (**b**) "GO_Molecular_Function_2018", (**c**)

"GO_Biological_Process_2018", (**d**) “GO_Cellular_Component_2018”. Terms for reduced heatmaps were chosen on the basis of relevance to processes related to stem cell differentiation, mitosis, transcription, translation, cell remodelling and mitochondrial respiration.
In GO databases, enriched pathways were often related to the same process. They were grouped by parent term using rrvgo R package (threshold of reduction: 0.7) and reduced to child terms that were enriched in the most clusters.

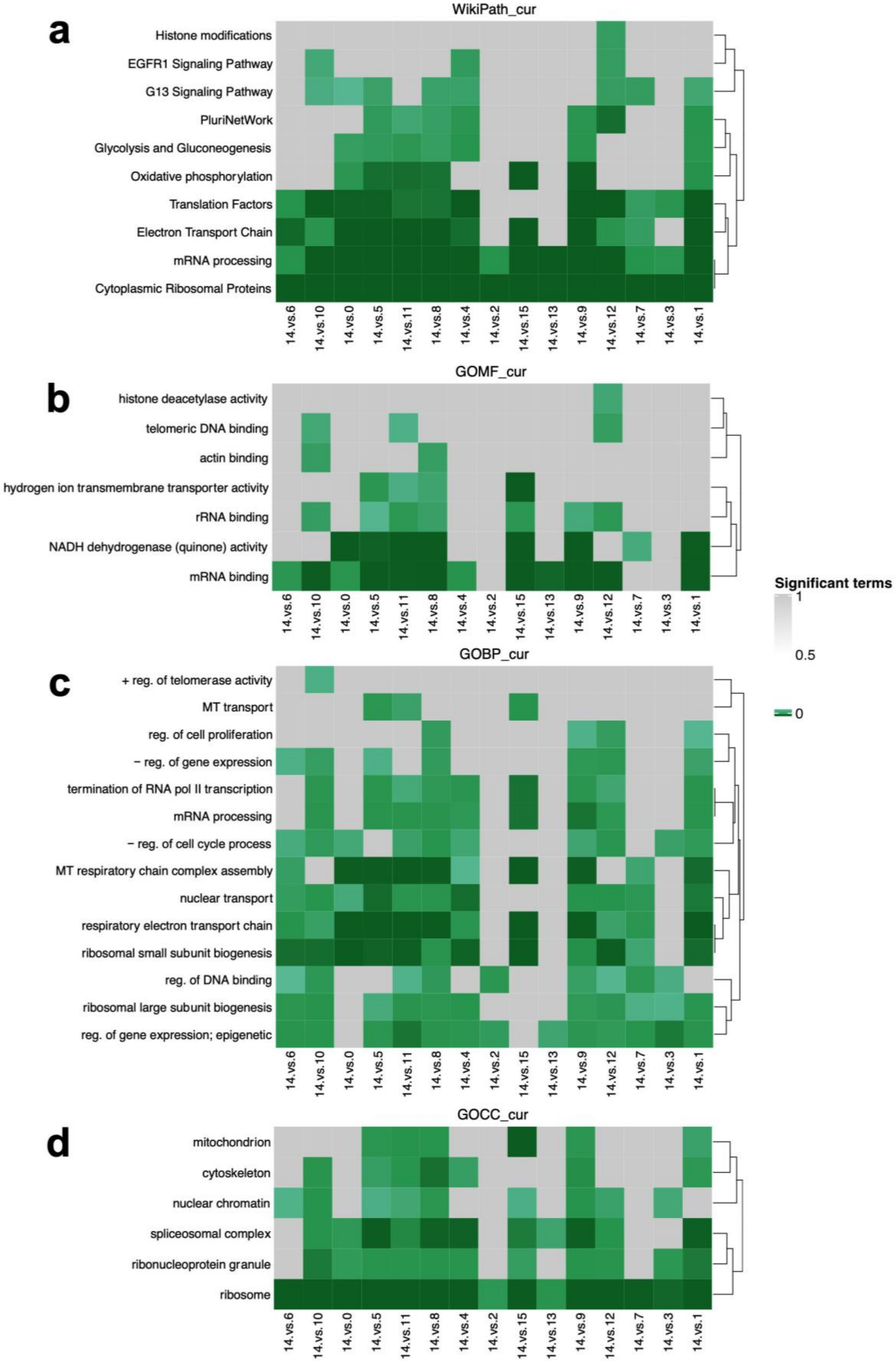
a
WikiPath_cur
Histone modifications
EGFR1 Signaling Pathway
G13 Signaling Pathway
PluriNetWork
Glycolysis and Gluconeogenesis
Oxidative phosphorylation
Translation Factors
Electron Transport Chain
mRNA processing
Cytoplasmic Ribosomal Proteins
14.vs.6
14.vs.10
14.vs.0
14.vs.5
14.vs.11
14.vs.8
14.vs.4
14.vs.2
14.vs.15
14.vs.13
14.vs.9
14.vs.12
14.vs.7
14.vs.3
14.vs.1
b
GOMF_cur
histone deacetylase activity
telomeric DNA binding
actin binding
hydrogen ion transmembrane transporter activity
rRNA binding
NADH dehydrogenase (quinone) activity
mRNA binding
Significant terms
1
0.5
0
c
GOBP_cur
+ reg. of telomerase activity
MT transport
reg. of cell proliferation
− reg. of gene expression
termination of RNA pol II transcription
mRNA processing
− reg. of cell cycle process
MT respiratory chain complex assembly
nuclear transport
respiratory electron transport chain
ribosomal small subunit biogenesis
reg. of DNA binding
ribosomal large subunit biogenesis
reg. of gene expression; epigenetic
d
GOCC_cur
mitochondrion
cytoskeleton
nuclear chromatin
spliceosomal complex
ribonucleoprotein granule
ribosome

Supplementary Figure 8. Enrichment analysis for Harmony-integrated Remove TSC differentiation dataset.

Heatmaps of selected terms from pathway enrichment using databases: (**a**) "WikiPathways_2019_Mouse", (**b**) "GO_Molecular_Function_2018", (**c**) "GO_Biological_Process_2018", (**d**) “GO_Cellular_Component_2018”. Terms for reduced heatmaps were chosen on the basis of relevance to processes related to stem cell differentiation, mitosis, transcription, translation, cell remodelling and mitochondrial respiration.

In GO databases, enriched pathways were often related to the same process. They were grouped by parent term using rrvgo R package (threshold of reduction: 0.7) and reduced to child terms that were enriched in the most clusters.

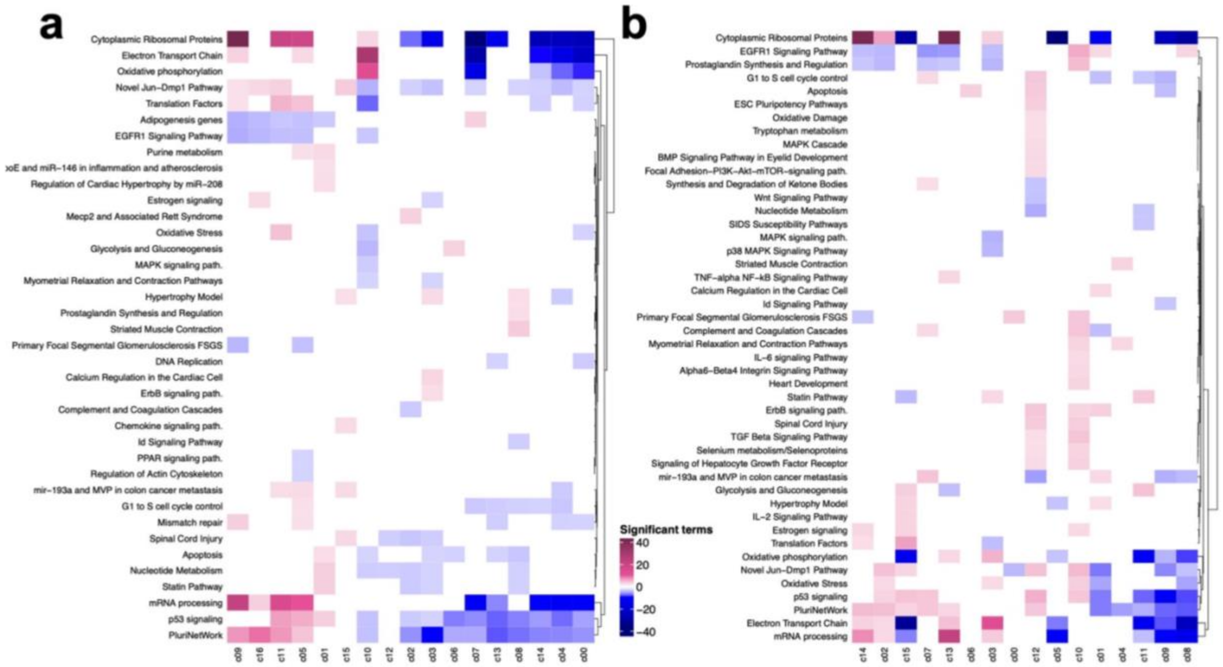

Supplementary Figure 9. Activity of enriched pathways in Inhibit and Remove datasets.
(**a-b**) Heatmap of gene expression in top WikiPathways in the (**a**) Inhibit and (**b**) Remove dataset. Pink colour represents enrichment of pathway in specific cluster compared to other clusters, blue colour represents pathways that are underrepresented in specific cluster compared to other clusters. Cluster specific variable genes, identified by comparing average normalised expression for each gene on scatterplots (see Methods for details), were used to calculate pathway enrichment for individual clusters.

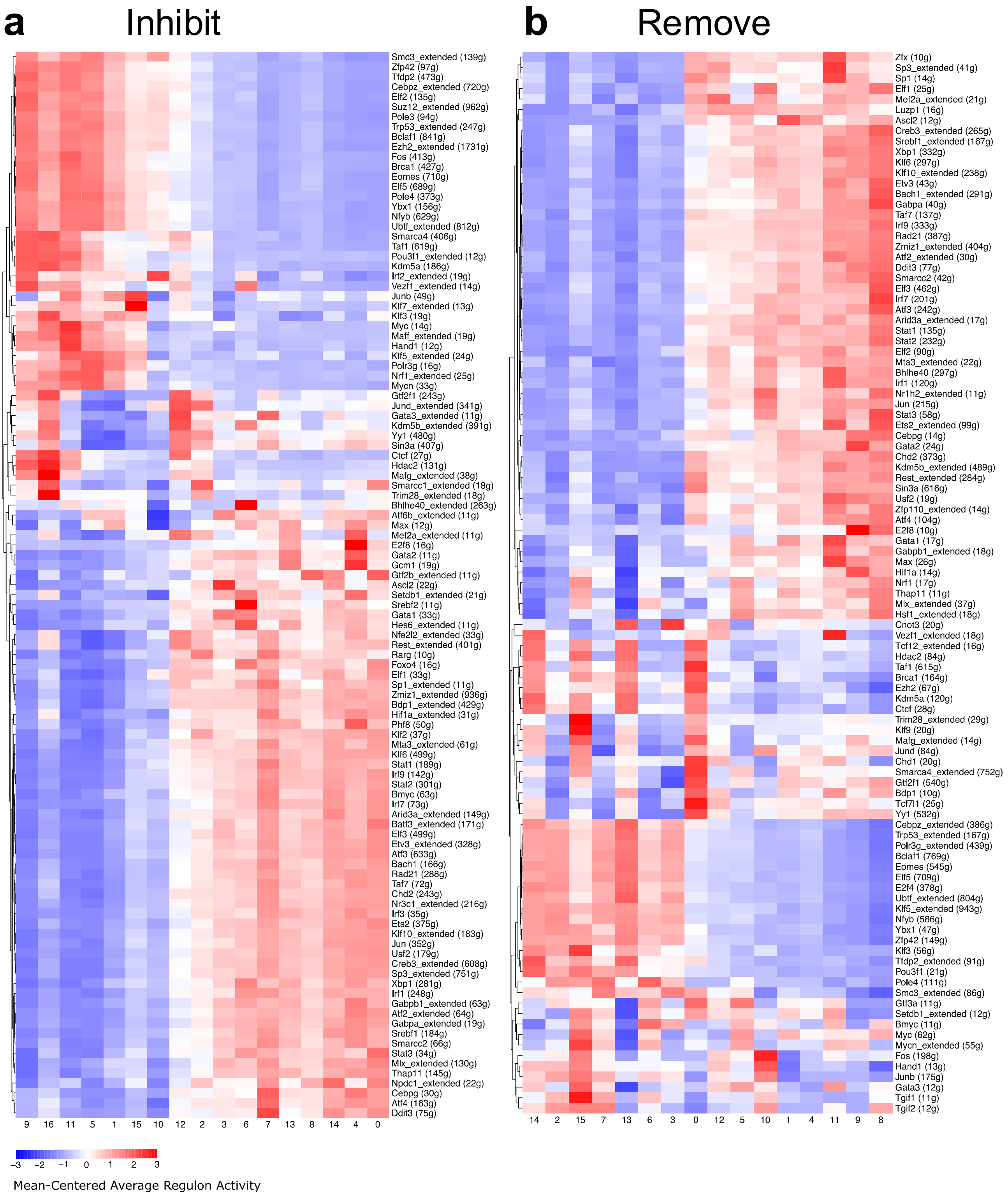

Supplementary Figure 10. SCENIC analysis.

Heatmap of significant transcription factor regulons detected in the dataset with SCENIC in the Inhibit (**a**) and Remove datasets (**b**). Colour represents average score for the activity of the regulon for each cluster. All values were mean centred for each regulon. Clusters were ordered by Tempora time scoring. SCENIC evaluates the activity of the gene regulatory networks in each cell by first identification of sets of genes that are co-expressed with transcription factors, then removing false positives and indirect targets by cis-regulatory motif analysis using RCisTarget and scoring each cell for regulon activity using the AUCell algorithm.

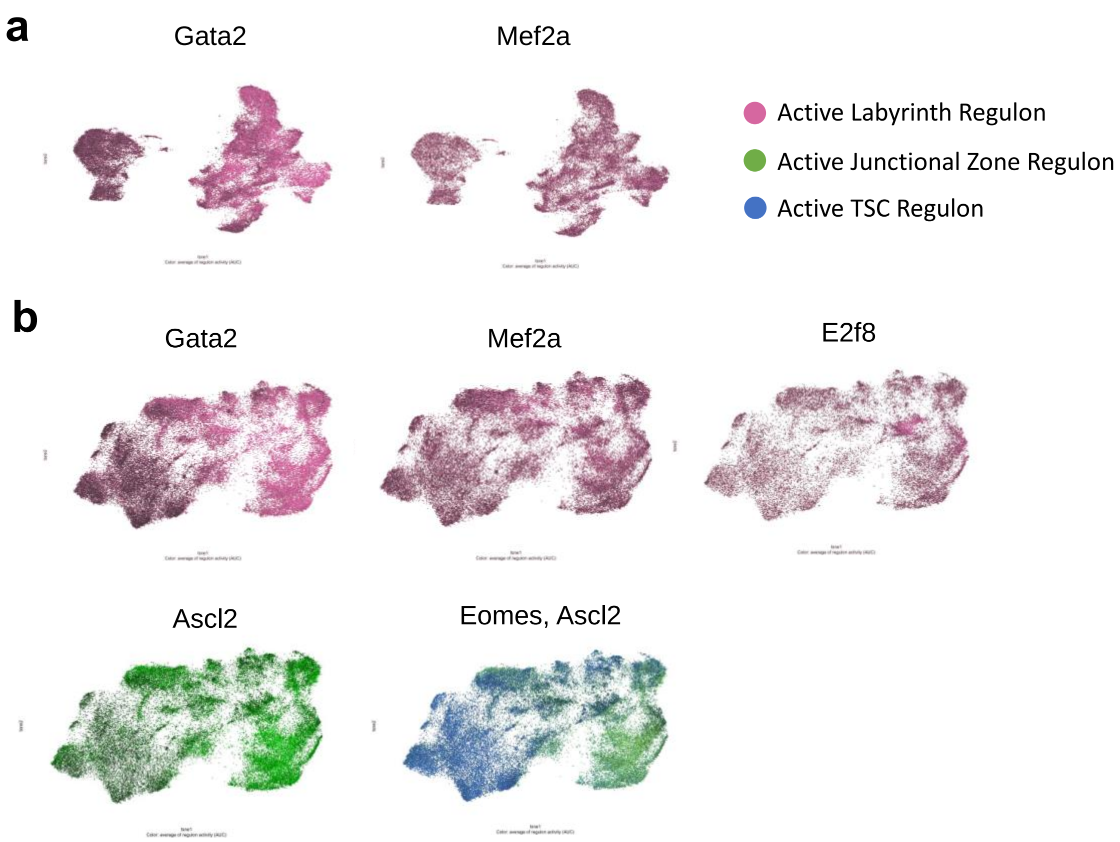

Supplementary Figure 11. UMAPs representing regulon activity in TSC dataset.
Specific SCENIC transcription factor regulons co-localize with TSCs, and cells associated with JZP and LP lineage. Cells were coloured by AUC score for regulons of interest. (**a**) UMAP plot of Inhibit dataset coloured by regulon activity: *Gata2*, *Mef2a*. (**b**) UMAP plot of Remove dataset coloured by regulon activity, calculated in SCENIC. The TSCs-specific regulon *Eomes* is coloured blue, JZP regulon *Ascl2* - coloured green.

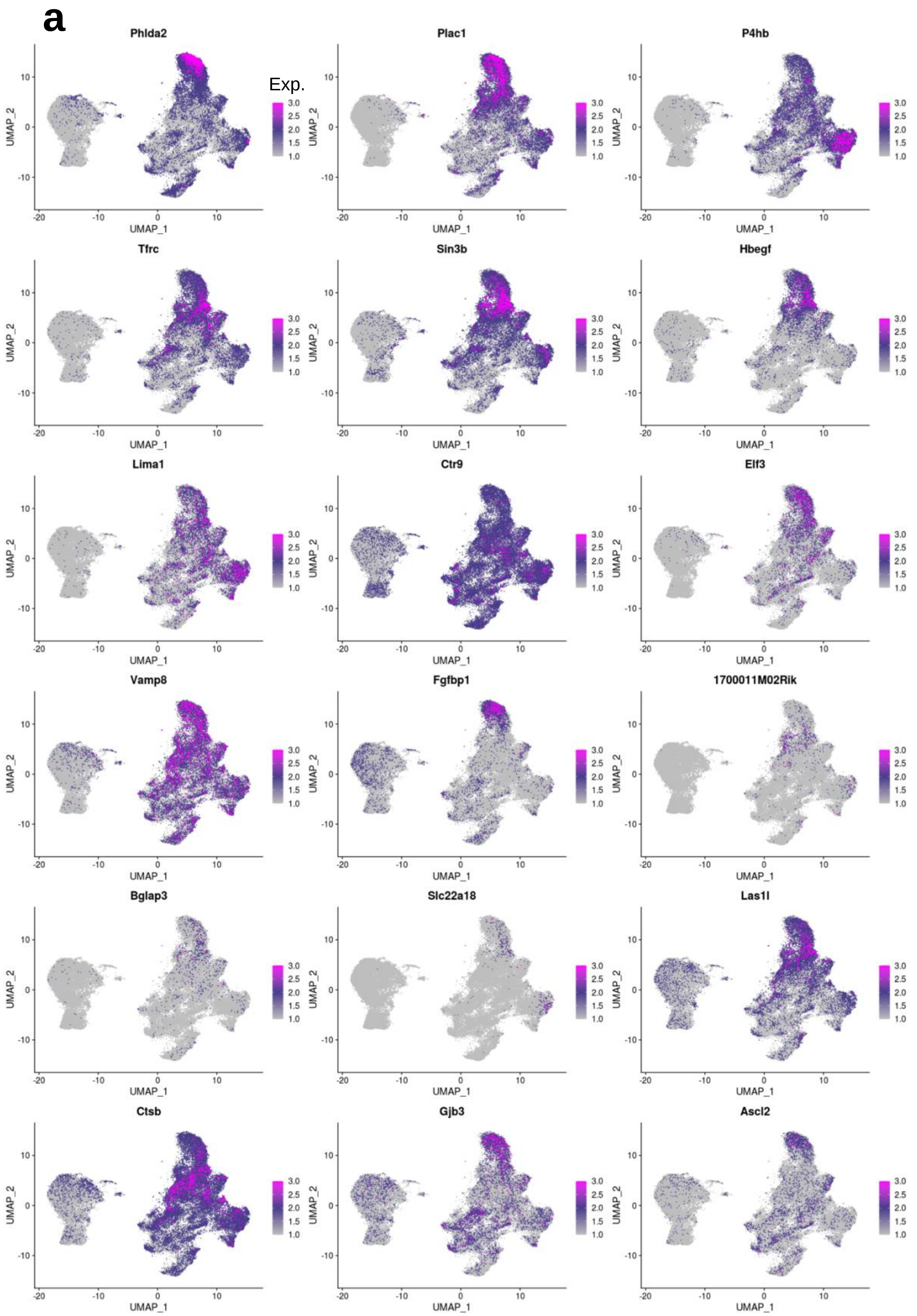
a
Exp.
Phlda2
Plac1
P4hb
Tfrc
Sin3b
Hbegf
Lima1
Ctr9
Elf3
Vamp8
Fgfbp1
1700011M02Rik
Bglap3
Slc22a18
Las1l
Ctsb
Gjb3
Ascl2
UMAP_1
UMAP_2

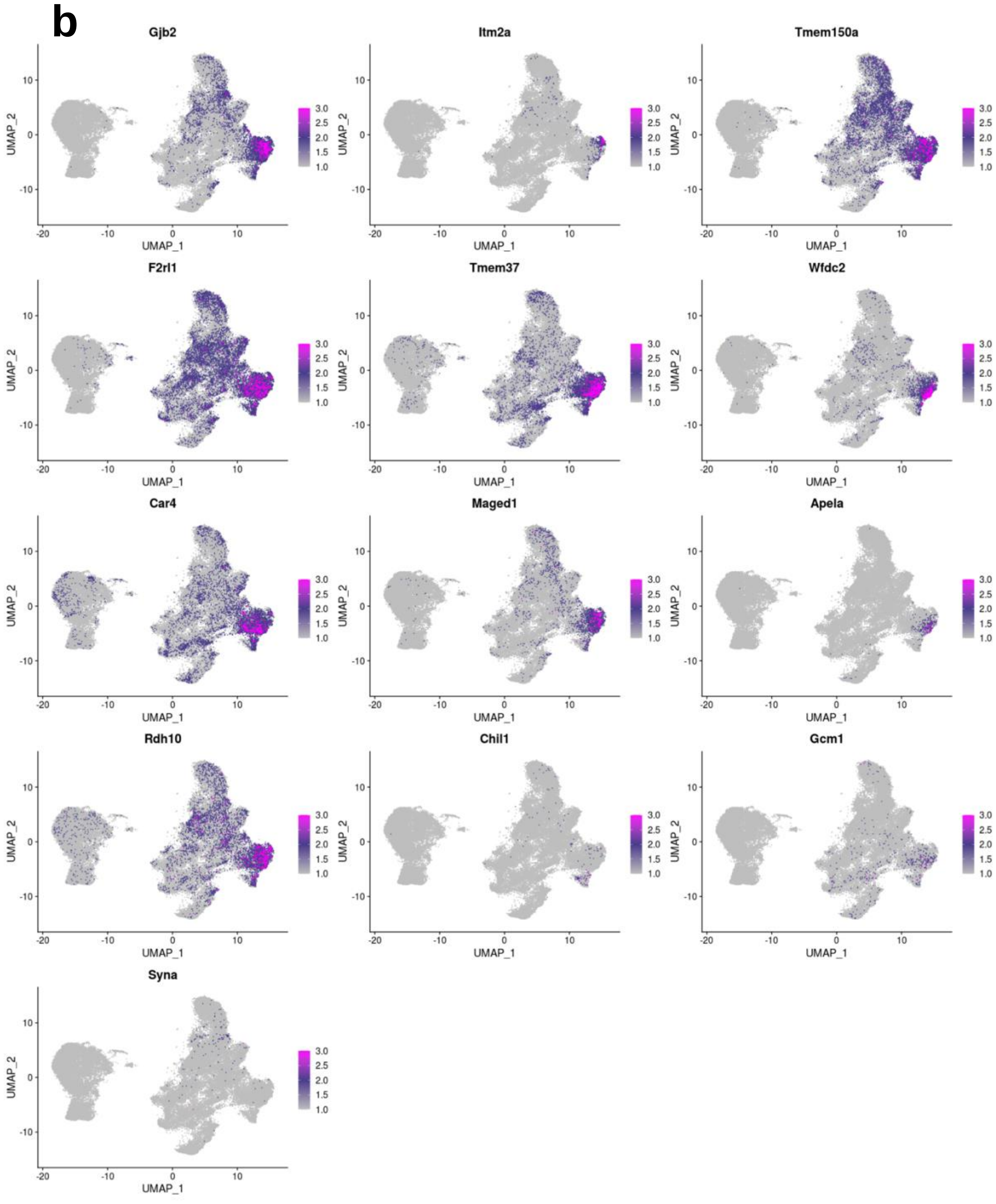
b
Gjb2
Itm2a
Tmem150a
F2rl1
Tmem37
Wfdc2
Car4
Maged1
Apela
Rdh10
Chil1
Gcm1
Syna
UMAP_1
UMAP_2
3.0
2.5
2.0
1.5
1.0

c

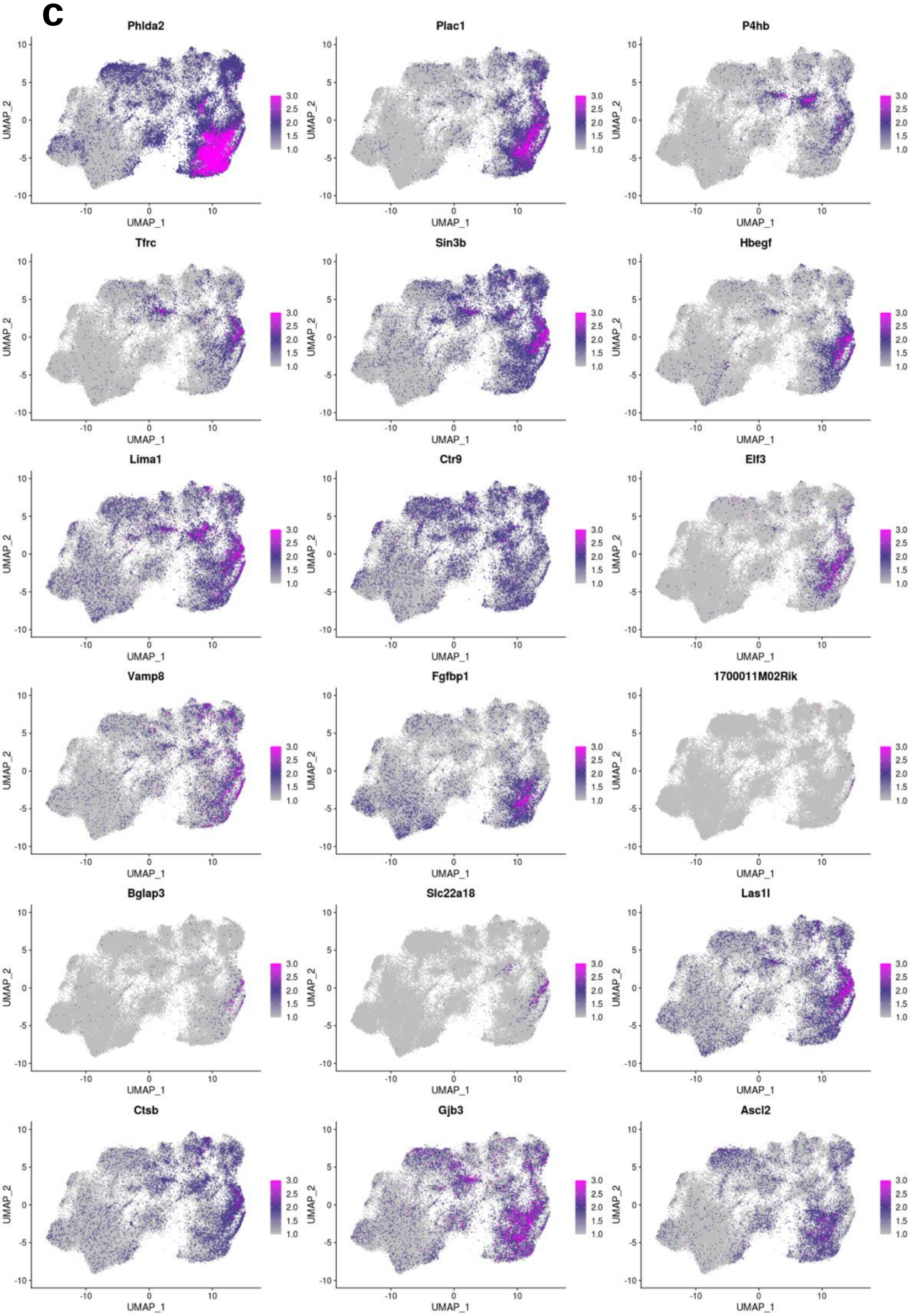
Phlda2
Plac1
P4hb
Tfrc
Sin3b
Hbegf
Lima1
Ctr9
Elf3
Vamp8
Fgfbp1
1700011M02Rik
Bglap3
Slc22a18
Las1l
Ctsb
Gjb3
Ascl2
UMAP_1
UMAP_2

d

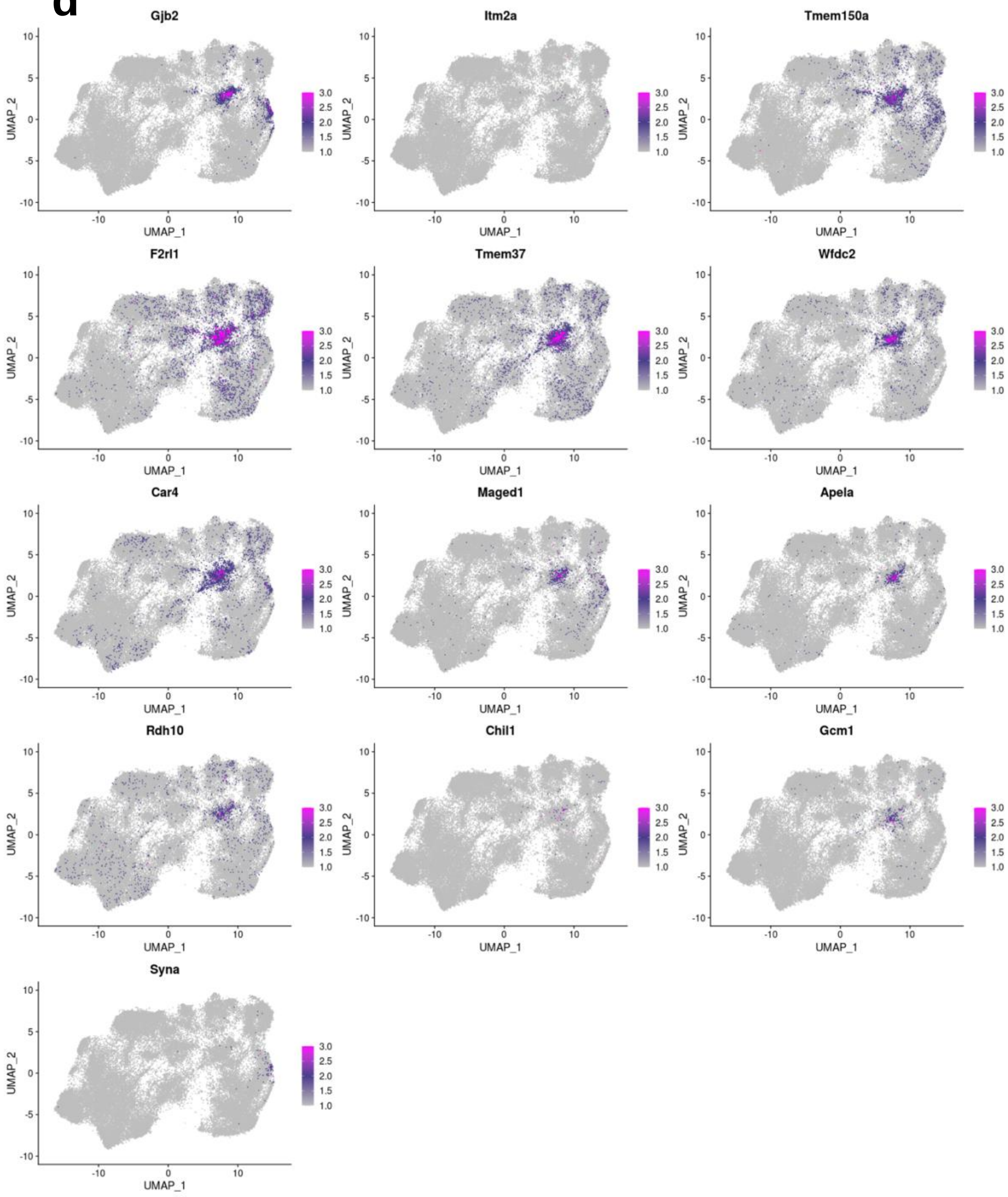

Supplementary Figure 12. JZP- and LP-specific genes associated with gene modules identified by Monocle3

Monocle pseudotime – UMAPs with markers and top genes for the LB and the JZP gene expression modules in Inhibit and Remove datasets. Genes of interest were identified by selecting the 10 genes with the highest Monocle score associated with the module in the Inhibit and Remove datasets, plus any known marker genes present in the module and merging the lists. Cells were coloured by gene expression. All values were mean centered for each gene. (**a**) Inhibit dataset JZP module (**b**) Inhibit dataset LP module (**c**) Remove dataset JZP module (**d**) Remove dataset LP module

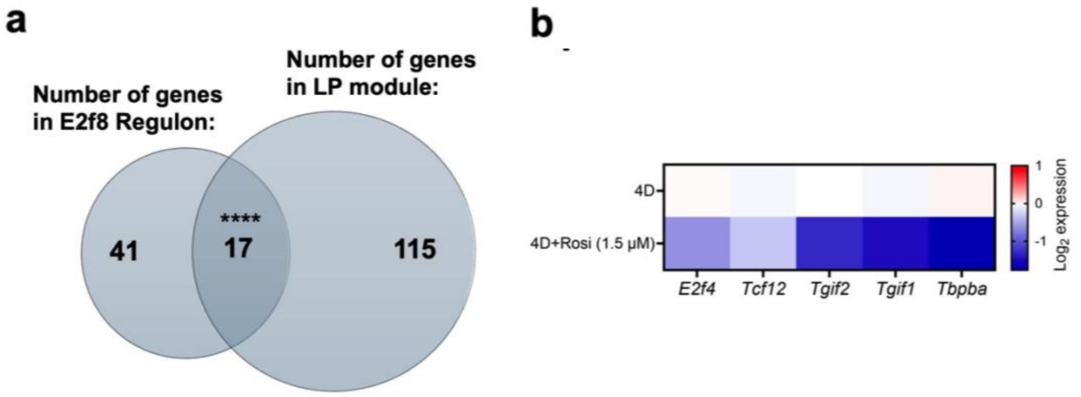

Supplementary Figure 13. Pseudotime and Scenic analysis.
(**a**) Venn diagram of overlap of genes in the LP module from Monocle analysis and the gene targets of E2F8 from SCENIC analysis **** p<0.0001 (hypergeometric test). (**b**) Change in expression of JZ markers after 4 days under Remove conditions +/- rosiglitazone treatment

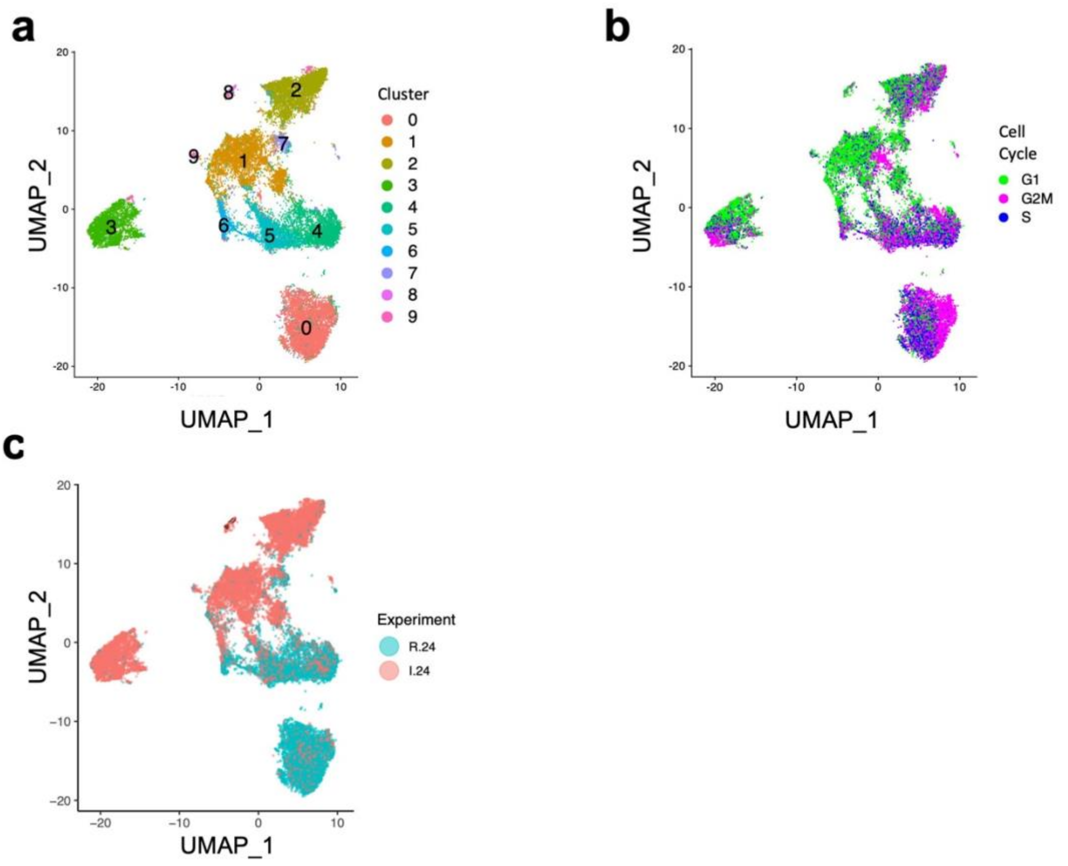

Supplementary Figure 14. UMAP grid of the Harmony-integrated t24 TSC differentiation dataset. Dimensional reduction of the dataset using UMAP after harmony integration. (**a**) UMAP coloured by cluster, resolution = 0.4. (**b**) UMAP coloured by assigned cell cycle phase (red=G2M, blue=S, green=G1) using the Seurat function CellCycleScoring, (**c**) UMAP with cells coloured by treatment (pink = Inhibit t24 and teal = Remove t24).

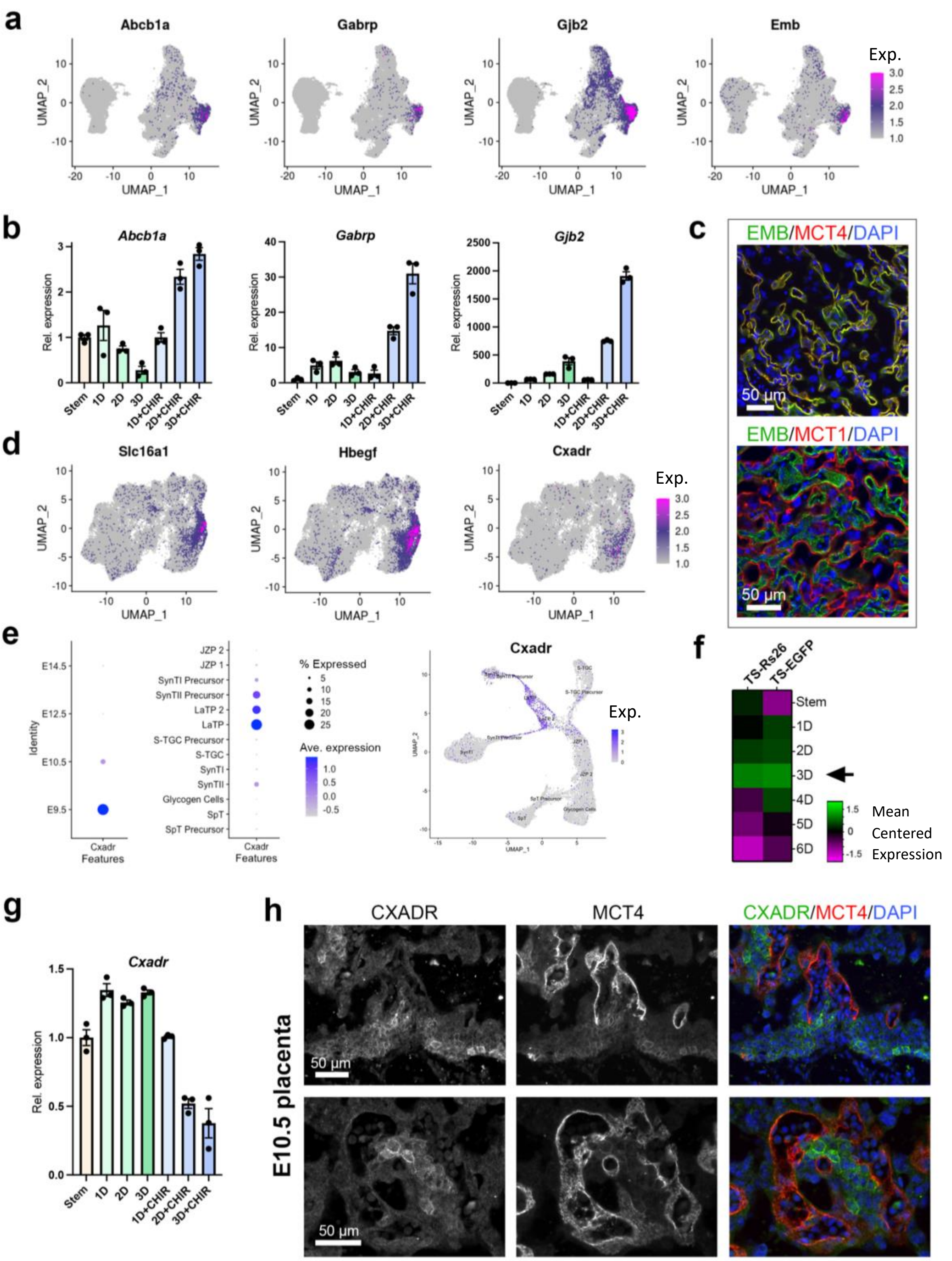

Supplementary Figure 15. Characterisation of TSC differentiation trajectories.
(**a**) UMAP plots on the Inhibit data of known (*Gjb2*) or newly identified, presumptive SynT-II markers. (**b**) RT-qPCR for *Abcb1a, Gabrp* and *Gjb2* on TSC in stem cell conditions and upon differentiation for 1-3 days in standard Remove conditions with and without the addition of CHIR99021 (CHIR). Addition of CHIR is known to drive SynT-II differentiation specifically. All three markers are hence SynT-II- enriched. Data are of three biological replicates, normalised to TSC ("Stem") values and shown as mean +/- SEM. (**c**) Immunofluorescence staining of E12.5 placenta for Embigin (EMB) co-

stained for SynT-II marker MCT4 as well as SynT-I marker MCT1. Images are representative of three independent experiments. (**d**) UMAP plots for *Slc16a1* (*Mct1*), *Hbegf* and *Cxadr* on the Remove dataset. *Slc16a1* and *Hbegf* are established SynT-I markers. (**e**) *Cxadr* enrichment in snRNA-seq data of placentas (Marsh & Blelloch, 2020). Analysis by developmental stage reveals highest expression at E9.5. Separation by cell type reveals *Cxadr* enrichment in labyrinth precursors. Pseudotime analysis highlights enrichment specifically in labyrinth precursors at the junction of SynT-I and -II lineage bifurcation. (**f**) Heatmap of normalised expression data of *Cxadr* in two TSC lines across a 6-day differentiation time course in Remove conditions. Mean values shown are of three independent experiments. (**g**) RT-qPCR analysis of *Cxadr* in differentiating TSCs as in (**b**) shows lack of induction with SynT-II differentiation. Data are of three biological replicates, normalised to TSC (Stem) values and are shown as mean +/- SEM. (**h**) E10.5 placentas stained for CXADR (green) and SynT-II marker MCT4 (red). Images are representative of n=4 placentas.

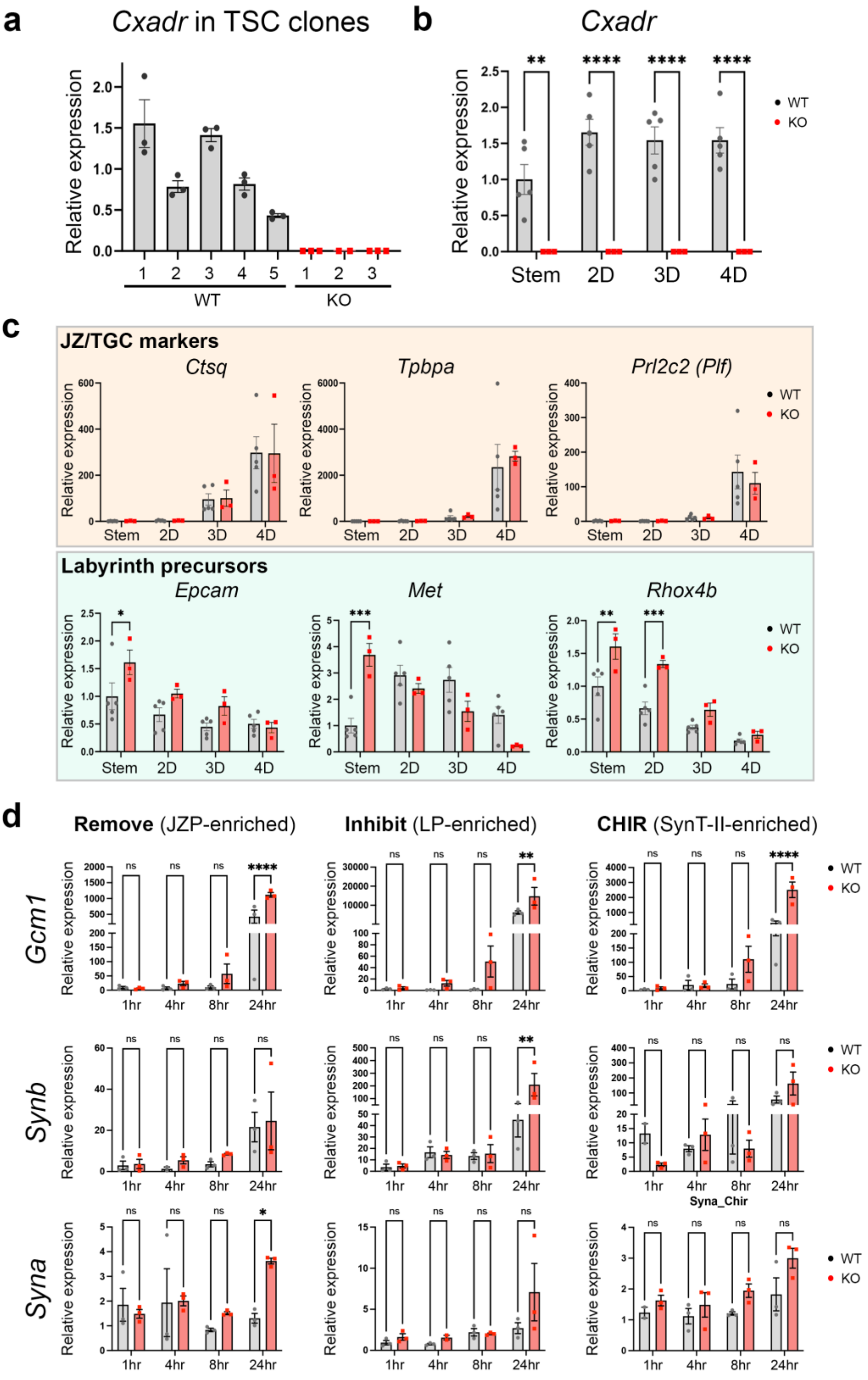

Supplementary Figure 16. Additional marker gene expression data on *Cxadr* KO TSCs.
(**a**) Confirmation of *Cxadr* ablation by RT-qPCR analysis of WT (n=5) and KO (n=3) TSC clones with primers positioned inside the deleted exon 2. Data are displayed as mean +/- SEM of three technical replicates per TSC clone. (**b**) RT-qPCR analysis of *Cxadr* expression in WT (n=5) and KO (n=3) TSC

clones grown in stem cell conditions and after and 2-, 3-, and 4-day differentiation in Remove conditions. Data are normalized to WT Stem conditions and are depicted as mean +/- SEM. (**c**) Relative expression levels of trophoblast cell type-specific markers between WT (n=5) and KO (n=3) TSC clones in stem cell conditions and after 2-, 3-, and 4-day differentiation in Remove conditions. (**d**) RT-qPCR analysis of syncytiotrophoblast markers *Gcm1* (SynT-II precursor), *Synb* (SynT-II) and *Syna* (SynT-I) in WT and *Cxadr* KO TSCs upon culture in the indicated conditions. Data in (**b**-**d**) are normalized to WT cells in stem cell conditions and are depicted as mean +/- SEM. Statistical significance was calculated using 2-way ANOVA. * $p<0.05$, ** $p<0.01$, *** $p<0.001$, **** $p<0.0001$.

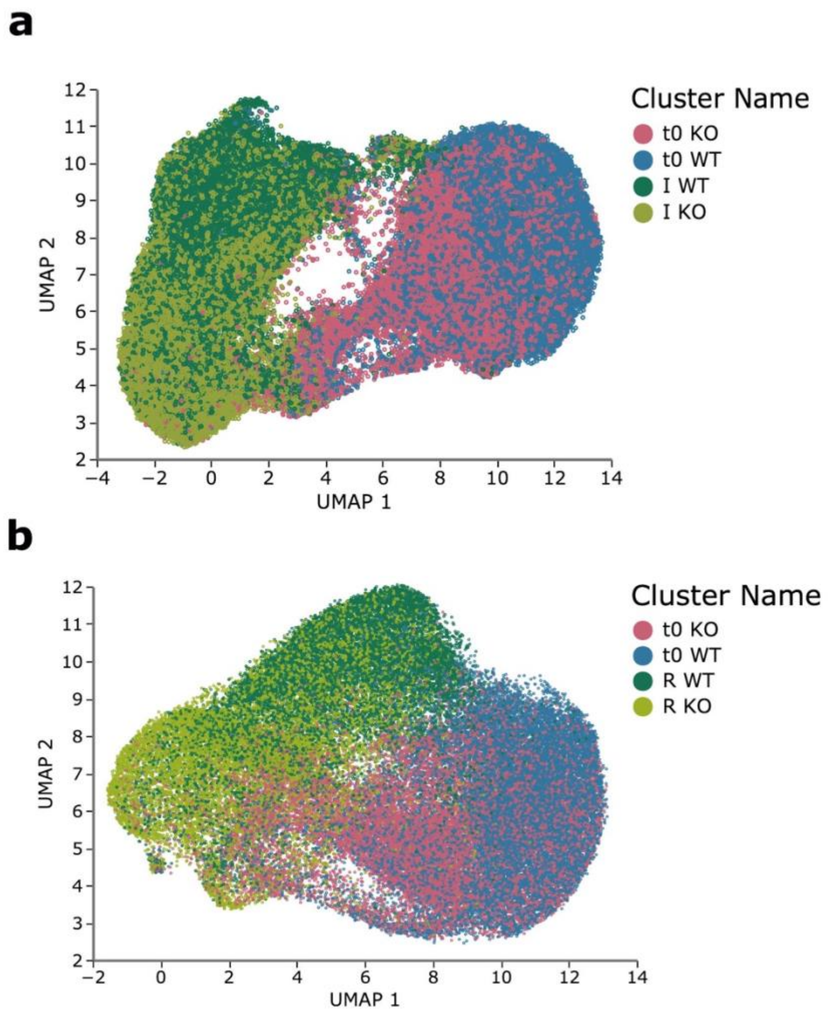

Supplementary Figure 17. Sample distribution of WT and *Cxadr* KO mTSCs.
(a-b). UMAP plots of mTSC datasets coloured by experimental condition - t0 in KO and WT cells and t24 I/R *Cxadr* WT and I/R *Cxadr* KO. **(a)** Inhibit dataset **(b)** Remove Dataset.

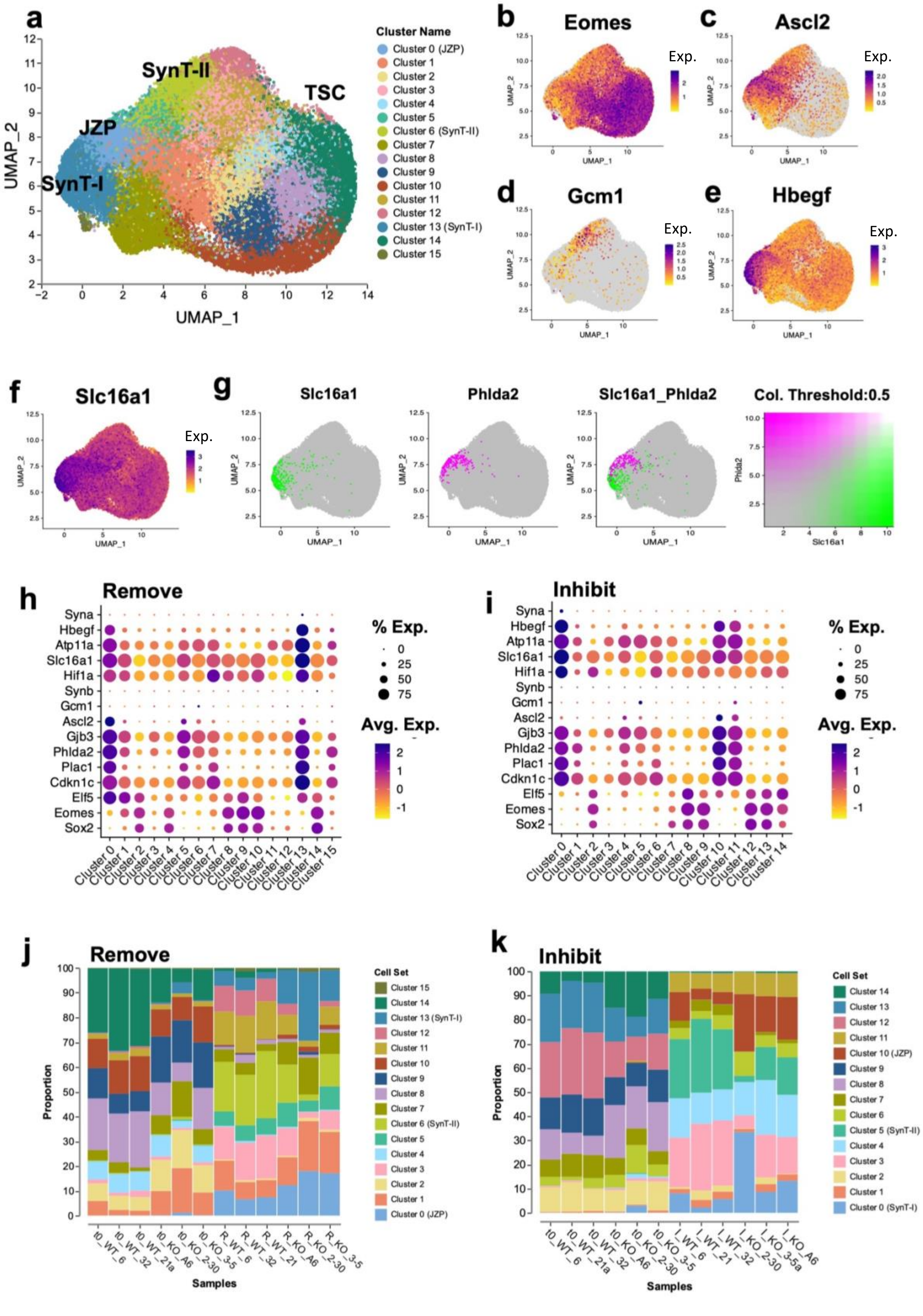

Supplementary Figure 18. Extended single cell RNA-seq analysis of *Cxadr* KO TSCs.
(**a**) UMAP of Remove dataset at t0 and t24 containing WT and *Cxadr* KO cells (resolution 0.7). Cluster 6 is labelled as SynT-II, Cluster 13 is labelled as SynT-I, Cluster 0 is labelled as JZP. (**b**) UMAP plot of

Eomes expression. (**c**) UMAP plot of *Ascl2* expression. (**d**) UMAP plot of *Gcm1* expression. (**e**) UMAP plot of *Hbegf* expression. (**f**) UMAP plot of *Slc16a1* expression. (**g**) UMAP plot of the locations of the top 10% cells by *Slc16a1* (green) and *Phlda2* (magenta) gene expression in the Remove dataset and the overlap between those populations (white). Exp. = expression (**h-i**) DotPlot of expression of trophoblast marker genes (TSC markers: *Sox2*, *Eomes*, *Elf5*; JZP markers: *Cdkn1c*, *Phlda2*, *Plac1*, *Gjb3*, *Ascl2*; SynT-II markers: *Gcm1*, *Synb*; SynT-I markers: *Hif1a*, *Slc16a1*, *Atp11a*, *Synb*, *Hbegf*) in the (**h**) Remove and (**i**) Inhibit dataset. For b-I Exp. = expression. (**j-k**) Stacked bar plot of the contribution of each cluster to each sample of origin ((**j**) Remove dataset, (**k**) Inhibit dataset).
