# Supplementary material for: Single-cell RNA sequencing identifies CXADR as a fate determinant of the placental exchange surface
Source: Nat Commun. 2025 Jan 2;16:142. doi: 10.1038/s41467-024-55597-w (PMC11695997; doi:10.1038/s41467-024-55597-w)
Supplement: Supplementary file 3 — Description of Additional Supplementary Files [file 41467_2024_55597_MOESM3_ESM.pdf]

Description of Additional Supplementary Files

Supplementary Data 1 - All sequencing samples sorted by run

Supplementary Data 2 - t0 dataset_Number of cells per cluster resolution= 0.4, 500gpc, Harmony integrated

Supplementary Data 3 – t0 dataset markers "Findallmarkers" with l2fc>0.25

Supplementary Data 4- t0 pairwise cl0 vs cl 2 l2fc>0.25

Supplementary Data 5 – Inhibit dataset_Number of cells per cluster resolution= 0.8, 500gpc, Harmony integrated

Supplementary Data 6 – Remove dataset_Number of cells per cluster resolution= 0.8, 500gpc , Harmony integrated

Supplementary Data 7 – Inhibit dataset markers "Findallmarkers" with l2fc>0.25

Supplementary Data 8 – Remove dataset markers "Findallmarkers" with l2fc>0.25

Supplementary Data 9 – Inhibit dataset_500g_res.0.8_P-val(adj) of Enriched pathways_BioCarta_matrix_l2fc0.25

Supplementary Data 10 – Inhibit dataset_500g_res.0.8_P-val(adj) of Enriched pathways_GOBP_matrix_l2fc0.25

Supplementary Data 11- Inhibit dataset_500g_res.0.8_P-val(adj) of Enriched pathways_GOCC_matrix_l2fc0.25

Supplementary Data 12 – Inhibit dataset_500g_res.0.8_P-val(adj) of Enriched pathways_GOMF_matrix_l2fc0.25

Supplementary Data 13 – Inhibit dataset_500g_res.0.8_P-val(adj) of Enriched pathways_Kegg_matrix_l2fc0.25

Supplementary Data 14 – Inhibit dataset_500g_res.0.8_P-val(adj) of Enriched pathways_WP_matrix_l2fc0.25

Supplementary Data 15 – Remove dataset_500g_res.0.8_P-val(adj) of Enriched pathways_BioCarta_matrix_l2fc0.25

Supplementary Data 16 – Remove dataset_500g_res.0.8_P-val(adj) of Enriched pathways_GOBP_matrix_l2fc0.25

Supplementary Data 17 – Remove dataset_500g_res.0.8_P-val(adj) of Enriched pathways_GOCC_matrix_l2fc0.25

Supplementary Data 18 – Remove dataset_500g_res.0.8_P-val(adj) of Enriched pathways_GOMF_matrix_l2fc0.25

Supplementary Data 19 – Remove dataset_500g_res.0.8_P-val(adj) of Enriched pathways_Kegg_matrix_l2fc0.25

Supplementary Data 20 – Remove dataset_500g_res.0.8_P-val(adj) of Enriched pathways_WP_matrix_l2fc0.25

Supplementary Data 21 - T24 dataset_Number of cells per cluster resolution= 0.4, 500gpc, Harmony integrated

Supplementary Data 22 - Guide RNA and primer sequences

Supplementary Data 23 – Individual Sample table

Supplementary Data 24 – R2 sample to sample correlations (Single cell to bulk)

Supplementary Data 25 – Merged Sample table

Supplementary Data 26 – Integration Methods
