# Supplementary material for: Single-cell RNA sequencing identifies CXADR as a fate determinant of the placental exchange surface
Source: Nat Commun. 2025 Jan 2;16:142. doi: 10.1038/s41467-024-55597-w (PMC11695997; doi:10.1038/s41467-024-55597-w)
Supplement: Supplementary file 5 — Reporting Summary [file 41467_2024_55597_MOESM5_ESM.pdf]

nature portfolio

Corresponding author(s): D. S. Charnock-Jones and M Hemberger

Last updated by author(s): Dec 4, 2024

Reporting Summary

Nature Portfolio wishes to improve the reproducibility of the work that we publish. This form provides structure for consistency and transparency in reporting. For further information on Nature Portfolio policies, see our Editorial Policies and the Editorial Policy Checklist.

Statistics

For all statistical analyses, confirm that the following items are present in the figure legend, table legend, main text, or Methods section.

n/a	Confirmed	
☐	☒	The exact sample size (*n*) for each experimental group/condition, given as a discrete number and unit of measurement
☐	☒	A statement on whether measurements were taken from distinct samples or whether the same sample was measured repeatedly
☐	☒	The statistical test(s) used AND whether they are one- or two-sided<br>*Only common tests should be described solely by name; describe more complex techniques in the Methods section.*
☒	☐	A description of all covariates tested
☐	☒	A description of any assumptions or corrections, such as tests of normality and adjustment for multiple comparisons
☐	☒	A full description of the statistical parameters including central tendency (e.g. means) or other basic estimates (e.g. regression coefficient) AND variation (e.g. standard deviation) or associated estimates of uncertainty (e.g. confidence intervals)
☐	☒	For null hypothesis testing, the test statistic (e.g. *F*, *t*, *r*) with confidence intervals, effect sizes, degrees of freedom and *P* value noted<br>*Give P values as exact values whenever suitable.*
☒	☐	For Bayesian analysis, information on the choice of priors and Markov chain Monte Carlo settings
☒	☐	For hierarchical and complex designs, identification of the appropriate level for tests and full reporting of outcomes
☐	☒	Estimates of effect sizes (e.g. Cohen's *d*, Pearson's *r*), indicating how they were calculated

Our web collection on statistics for biologists contains articles on many of the points above.

Software and code

Policy information about availability of computer code

Data collection	The standard Illumina sequencing machine software was used to collect RNA sequencing data
Data analysis	Cutadapt (v4.0), fastp (v0.23.4) and BBTools (v39.05), DESeq2 (v. 1.30), stats (v3.5.3), hmisc (v4.5.0), clustree (v.0.4.3), Seurat (v.3.2.0), sctransform (v.0.3.2), Harmony (v.1.0), Tempora (v.0.1.0), Monocle3 (v.0.1.2), speckle (v1.2.0), enrichR (v.3.0), ComplexHeatmap (v.1.20.0), rrvgo (v 1.1.3), SCENIC (v.1.1.2-2), Arboreto (v.0.1.6), Pheatmap (v.1.0.12), GraphPad (10.0.2)<br>Demultiplexing of raw fastq files into Nextera indexed fastq files was performed using a custom script (dropseq_demultiplex.sh).<br>Trailmaker: Available at: http://app.trailmaker.parsebiosciences.com Analysis performed on: 28/08/2024<br>Code used in this study is available at GitHub doi: 10.5281/zenodo.10159534

For manuscripts utilizing custom algorithms or software that are central to the research but not yet described in published literature, software must be made available to editors and reviewers. We strongly encourage code deposition in a community repository (e.g. GitHub). See the Nature Portfolio guidelines for submitting code & software for further information.

Data

Policy information about availability of data

All manuscripts must include a data availability statement. This statement should provide the following information, where applicable:

- Accession codes, unique identifiers, or web links for publicly available datasets
- A description of any restrictions on data availability
- For clinical datasets or third party data, please ensure that the statement adheres to our policy

All the computational analyses were conducted using the Linux clusters at the University of Cambridge High Performance Computing Service and the Linux workstations of School of Biological Science computing. The RNA-Seq datasets generated during the current study are available in the European nucleotide archive (ENA, https:// www.ebi.ac.uk/ena) with the following accession code: PRJEB68188. The Seurat R objects, Monocle and SCENIC analysis results are available in Figshare: [https://figshare.com/s/2eb6b0b653070e0a48c2]

Research involving human participants, their data, or biological material

Policy information about studies with human participants or human data. See also policy information about sex, gender (identity/presentation), and sexual orientation and race, ethnicity and racism.

Reporting on sex and gender	N/A no human participants or biological material were used in this study
Reporting on race, ethnicity, or other socially relevant groupings	N/A
Population characteristics	N/A
Recruitment	N/A
Ethics oversight	N/A

Note that full information on the approval of the study protocol must also be provided in the manuscript.

Field-specific reporting

Please select the one below that is the best fit for your research. If you are not sure, read the appropriate sections before making your selection.

☒ Life sciences ☐ Behavioural & social sciences ☐ Ecological, evolutionary & environmental sciences

For a reference copy of the document with all sections, see nature.com/documents/nr-reporting-summary-flat.pdf

Life sciences study design

All studies must disclose on these points even when the disclosure is negative.

Sample size	Based on our extensive experience with mouse KO TSC lines and considering the necessity to perform qPCR reactions within the same microwell plate for all genes analysed, at least three independent KO clones and at least three independent wild-type clones that were derived in parallel to each KO from the same starting TSC population, were obtained from every CRISPR-Cas9 KO experiment targeting Cxadr and Nicol1. RT-qPCRs for trophoblast stem cell and differentiation markers were run in triplicate on samples from 3 to 5 time-points for three KOs, as well as the corresponding three wild-type clones that were co-derived in parallel to the KO clones from the same parental TSC population.
Data exclusions	In the Drop-Seq single cell analysis cells with fewer than 500 genes detected were removed.
Replication	Single cell sequence was repeat using 3-4 independent cultures of trophoblast stem cells at each time point. When KO TSCs were used 3 independent closes were used
Randomization	Libraries (ie samples) were randomly assigned to sequencing batches
Blinding	Measurements of TSC fusion indices of wild-type and Cxadr KO cells were conducted in a blinded manner. For RT-qPCR data from wild-type and KO TSCs the data were not blinded as the entire methodology was based on computational quantifications and no bias could have been introduced.

Reporting for specific materials, systems and methods

We require information from authors about some types of materials, experimental systems and methods used in many studies. Here, indicate whether each material, system or method listed is relevant to your study. If you are not sure if a list item applies to your research, read the appropriate section before selecting a response.

Materials & experimental systems

n/a	Involved in the study
☐	☒ Antibodies
☐	☒ Eukaryotic cell lines
☒	☐ Palaeontology and archaeology
☐	☒ Animals and other organisms
☒	☐ Clinical data
☒	☐ Dual use research of concern
☒	☐ Plants

Methods

n/a	Involved in the study
☒	☐ ChIP-seq
☒	☐ Flow cytometry
☒	☐ MRI-based neuroimaging

Antibodies

Antibodies used

CXADR 1:100, R&D Systems AF2654, lot number VFT012012
MCT1 1:100, EMD Millipore AB1286-I, lot number 3824852
MCT4 1:100, EMD Millipore AB3314P, lot number 4021707
EMBIGIN 1:100, ThermoFisher Scientific 12-5839-82, lot number 2134158
ZO1 1:200, ThermoFisher Scientific 339100, lot number YD370969
NCAM1 1:100, R&D AF2408-SP, lot number VOK0223031
STRA6 1:100, Novus Biologicals NBP3-12353, lot number A764.Pb1.AP
SOX2 1:200, R&D AF2018, lot number KOY0420121
KRT18 1:100, Research Diagnostics RDI-PRO 61028, lot number 103259
NICOL1 1:100, St Johns STJ196219, lot number 9RC112RC11

Validation

CXADR, single band on western blot (manufacture's product data sheet)
MCT1 and MCT4 staining patterns are well-established and are localized to SynT-I and SynT-II, respectively. The antibodies were verified in double IF staining experiments to recapitulate this exact staining pattern on mouse placentas this shows juxtaposition but non-overlap of staining patterns in the placental labyrinth, as expected (see Fig. 6g).
EMBIGIN antibody has been used in FACS analysis, doi: 10.1016/j.stem.2019.06.003.
ZO1 antibody validated by siRNA mediated knockdown (manufacture's website).
NCAM1 antibody knock-out validated (manufacture's website)
SOX2, single band on western blot and nuclear staining in stem cells (manufacture's product data sheet)
STRA6, KRT18 and NICOL1 antibodies exhibited the expected staining pattern (subcellular distribution, intensity staining on TSC differentiation) as expected from other supporting data in our manuscript.
All secondary antibodies were verified in no-primary antibody control staining experiments that produced no signals.

Eukaryotic cell lines

Policy information about cell lines and Sex and Gender in Research

Cell line source(s)

TSC lines used in this study were TS-EGFP and TS-Rs26, both a kind gift of the Rossant laboratory (Toronto, Canada). Tanaka, S., Kunath, T., Hadjantonakis, A.-K., Nagy, A. & Rossant, J. Promotion of Trophoblast Stem Cell Proliferation by FGF4. Science 282, 2072–2075 (1998). The sex of the cell lines was female.

Authentication

TSCs express markers distinct in their combination for trophoblast stem cells, and are capable of differentiating into syntytiotrophoblast and trophoblast giant cells, again cell types that are unique to the trophoblast lineage.

Mycoplasma contamination

Upon arrival to the lab and prior to knock-out generation via CRISPR, stock TS-Rs26 cells were tested for mycoplasma using a PCR kit (e-Myco, Cat. No 25233, iNtRON Biotechnology DR). All stocks were negative. All cell lines are routinely tested for mycoplasma every 6 months, and are proven mycoplasma-free.

Commonly misidentified lines
(See ICLAC register)

None used

Animals and other research organisms

Policy information about studies involving animals; ARRIVE guidelines recommended for reporting animal research, and Sex and Gender in Research

Laboratory animals

Species: Mus musculus. Strain: C57BL/6N. Age range: 8-20 weeks.

Wild animals

Provide details on animals observed in or captured in the field; report species and age where possible. Describe how animals were

Wild animals	*caught and transported and what happened to captive animals after the study (if killed, explain why and describe method; if released, say where and when) OR state that the study did not involve wild animals.*
Reporting on sex	*Indicate if findings apply to only one sex; describe whether sex was considered in study design, methods used for assigning sex. Provide data disaggregated for sex where this information has been collected in the source data as appropriate; provide overall numbers in this Reporting Summary. Please state if this information has not been collected. Report sex-based analyses where performed, justify reasons for lack of sex-based analysis.*
Field-collected samples	*For laboratory work with field-collected samples, describe all relevant parameters such as housing, maintenance, temperature, photoperiod and end-of-experiment protocol OR state that the study did not involve samples collected from the field.*
Ethics oversight	All animal work was conducted with approval by the University of Calgary's animal care committee, and with appropriate Health Sciences Animal Care Committee (HSACC)-approved animal use protocols in place (protocol number AC18-0191).

Note that full information on the approval of the study protocol must also be provided in the manuscript.

Plants

Seed stocks	*Report on the source of all seed stocks or other plant material used. If applicable, state the seed stock centre and catalogue number. If plant specimens were collected from the field, describe the collection location, date and sampling procedures.*
Novel plant genotypes	*Describe the methods by which all novel plant genotypes were produced. This includes those generated by transgenic approaches, gene editing, chemical/radiation-based mutagenesis and hybridization. For transgenic lines, describe the transformation method, the number of independent lines analyzed and the generation upon which experiments were performed. For gene-edited lines, describe the editor used, the endogenous sequence targeted for editing, the targeting guide RNA sequence (if applicable) and how the editor was applied.*
Authentication	*Describe any authentication procedures for each seed stock used or novel genotype generated. Describe any experiments used to assess the effect of a mutation and, where applicable, how potential secondary effects (e.g. second site T-DNA insertions, mosiacism, off-target gene editing) were examined.*
