# Supplementary material for: Single-cell RNA sequencing identifies CXADR as a fate determinant of the placental exchange surface
Source: Nat Commun. 2025 Jan 2;16:142. doi: 10.1038/s41467-024-55597-w (PMC11695997; doi:10.1038/s41467-024-55597-w)
Supplement: Supplementary file 2 — Peer review File [file 41467_2024_55597_MOESM2_ESM.pdf]

nature portfolio

Peer Review File

Single-cell RNA sequencing identifies CXADR as a fate determinant of the placental exchange surface

Open Access This file is licensed under a Creative Commons Attribution 4.0 International License, which permits use, sharing, adaptation, distribution and reproduction in any medium or format, as long as you give appropriate credit to the original author(s) and the source, provide a link to the Creative Commons license, and indicate if changes were made. In the cases where the authors are anonymous, such as is the case for the reports of anonymous peer reviewers, author attribution should be to 'Anonymous Referee' followed by a clear attribution to the source work. The images or other third party material in this file are included in the article's Creative Commons license, unless indicated otherwise in a credit line to the material. If material is not included in the article's Creative Commons license and your intended use is not permitted by statutory regulation or exceeds the permitted use, you will need to obtain permission directly from the copyright holder. To view a copy of this license, visit http://creativecommons.org/licenses/by/4.0/.

REVIEWER COMMENTS

Reviewer #1 (Remarks to the Author):

In their manuscript titled 'Single Cell RNA Sequencing Identifies CXADR as a Determinant of Placental Exchange Surface Fate,' Angelova et al. employed a single-cell transcriptomics approach in combination with the time-course differentiation of mouse trophoblast stem cells (TSCs) using two distinct methods: Inhibition (inhibit MEK pathway) and Removal (CM and FGF4). The authors unveiled early trophoblast lineage diversification trajectories and identified multiple putative novel regulators. Specifically focusing on lineage specification towards junctional zone cell precursors (JZP) and labyrinth precursors (LP), the manuscript aimed to pinpoint molecular drivers. Notably, the authors functionally validated Cxadr as a key factor in balancing differentiation between the two syncytial layers of the mouse labyrinth.

While the data presented in the manuscript holds promise for offering novel insights into cell fate determinants controlling trophoblast lineage specification, it appears that the manuscript was prepared hastily. There are so many errors, inadequate descriptions in the text and legends, and inconsistent labels in the figures, and all these make it challenging to comprehend the findings. Although the validation of Cxadr is carried out, its importance in trophoblast differentiation has been previously reported. Given the prediction of multiple novel regulators from the current study, performing functional validations on a few newly identified factors would enhance the robustness and significance of the research.

Comments:

Are the expression profiles of LP and JZP cells in the current study similar to those observed in vivo? It is crucial to investigate whether they exhibit comparable expression patterns to bolster the significance of the study.

Concerns regarding Figure 1 and Figure S1: In the main text, the authors did not clearly specify the nomenclature for individual time course samples and each batch. In Figure S1a, only seven datasets are presented; are these distinct batches, and what does the identity on the X-axis (00C, 0B0, 0BC, etc.) signify? The figure legends lack adequate information in several instances. Furthermore, in Figure 2a, only four batches are depicted, leaving three batches unaccounted for (identified in Figure S1).

Regarding Line 111: The study predicts a small subset of cells already primed for differentiation, but ultimately, all cells appear to differentiate (Figure 3). If this holds true, what distinguishes primed TSCs from other TSCs, aside from minor expression differences? Are primed TSCs faster in differentiation?

Lines 112-124: To underscore the data's value in identifying critical TSC factors, such as Nicol1, additional validation may be essential. The main text highlights the identification of multiple novel regulators, emphasizing the necessity of functional validations for novel regulators associated with the JZP/LP lineages and SynT-II markers (line 383).

Concerning Line 145 and Figure S3: The heatmap provided does not correspond to the Remove data. Throughout the figures, there are instances of mislabeled panels, missing legends, and inconsistencies in labels.

Other specific points of correction include Line 158 (no Figure S3b), Line 176 (Gjb3 is not present), Line 181 (clarification on the correct number of top marker genes: 10 or 5?), and Figure 3K (inconsistencies in legends regarding the 17 clusters).

Line 219 raises a query about the definition of "regulon," necessitating detailed explanations and references if applicable. Similarly, Line 266 requests information on the source of E2F8 targets, which is insufficiently provided in multiple instances.

Supplementary Figure 8 lacks a description in the main text.

Line 396 and Figure 6a: The process of selecting Cxadr as a candidate regulator is unclear, especially as the expression patterns of Cxadr and Mct1 differ (Figure 6a). A detailed explanation is necessary.

Figure 7e is missing its legend.

Concerns about Figure 7b and Figure S15a are raised. Cxadr expresses only detectable levels in TSCs, yet KO confirmation was performed in TSCs.

Lines 518-520 call for the testing of Cxadr expression levels, considering the enrichment of JZP differentiation in Remove conditions and favored LP differentiation in Inhibit conditions.

The manuscript should incorporate a discussion on human trophoblast differentiation, particularly focusing on the multiple novel regulators predicted in the study, including CXADR.

Reviewer #2 (Remarks to the Author):

This manuscript by Angelova and colleagues entitled "Single cell RNA sequencing identifies CXADR as a fate determinant of the placental exchange surface”" used single cell RNA sequencing of mouse TSCs in their stem state culture and under two differentiating conditions, remove condition (removal FGF and MEF conditioned medium) and inhibit condition (chemical inhibition of MEK) to capture gene expression changes during early stages of trophoblast differentiation during mouse placentation. In particular, authors used scRNA-seq to better understand temporal progression of TSC differentiation to Syncytiotrophoblast lineage to better understand development of SynT-I and SynT-II populations. The authors further focused on specific role of the Cxadr gene, which was earlier shown to be important for placental labyrinth development. The authors generated a Cxadr-KO mouse TSC line and found that the loss of CXADR promotes SynT differentiation but severely diminishes Gcm1 expression in differentiating TSCs, a finding which recapitulated earlier observation in Cxadr mutant mice. Thus, the authors concluded that cell-autonomous function of Cxadr gene is a gatekeeper to balance SynT-I vs. SynT-II differentiation.

The study is interesting and provides novel information a better picture of gene expression dynamics during early stages of mouse trophoblast differentiation using mouse TSC as a model system. Thus, the study could be informative to the field.

However, enthusiasm is diminished due to breadth of approaches and conclusions are almost entire reliant on scRNA-seq data and associated RT-PCR data. Additional experimental approaches are necessary to make definitive conclusions about the claims made for phenotypic variations of the stem-state and differentiating cells. The conclusion about the role of Cxadr is confusing based on expression during TSC differentiation and phenotype of Cxadr-KO TSCs. Different experimental conditions for SynT differentiation for different experiments have also complicated the interpretation of the data. Given the already characterized placental phenotype of Cxadr-KO mouse model, the new data related to CXADR seems incremental. More analyses are necessary. My concerns are described below and should be addressed.

1. The entire characterization of cell types in stem and differentiating stages rely on data from scRNA-seq analyses and a fold change of ≥1.5 or ≥1.2. This is concerning and needs more validation. For example, as mentioned in the manuscript, it is well known that mouse TSC cultures have heterogeneous cell morphology and contains cells in true stem-state vs. differentiating state. However, the level of morphological heterogeneity varies from culture to culture and duration of culture. Thus, authors should show the images of TSC colonies that are representative of cultures that they have used for their scRNA-seq analyses in stem state as well as differentiating conditions (Time course).

2. The differentiating cells in stem state condition (t0 clusters 1 and 2 in Fig. 2a) needs better characterization for their differentiation trajectory. Does these cells have some induction of Hand1, Gcm1 and Cxadr? Additional experimental approaches (IHC, Immunofluorescence) are needed to show the markers that authors claim to be specific to true TSCs (such as Nicol1). This is important to show whether expression levels are varying in cells with different morphology. Authors should also test whether MEK activation reduces number cells of t0 clusters 1 and 2. Also, authors should also test that prolonged MEK inhibition is not inducing apoptosis markers.

3. As noted, mouse TSC differentiation system (especially the remove system) is not a very robust model for SynT differentiation due to transient and non-synchronous differentiation. Thus, data presented in Figs. 3, 4 and 5 and in Supplementary Figs. 5, 6 and 7 need to be supported by cell culture images (inhibit vs. remove conditions), and additional experimental approaches (IHC or IF) for marker expressions (such as E2F8, PHF8, Plac1, Klf8, B2m, Phlda2 etc). This will provide a better visual understanding of cell populations that were undergoing differentiation process. Does the inhibit condition clearly show SynT formation?

4. It is necessary to better characterize SynT-II only differentiation with CHIR. Cell colony images with MCT1 and MCT4 expression and SynT formation should be shown. The introduction of Emb gene is sudden (lines 378-379) in the manuscript and is not clear whether a similar expression pattern (overlapping with MCT4) was observed in the cell culture model.

5. The induction of Cxadr in Mct1-expressing cluster (Fig. 6a, Supplementary Fig. 14D) is different from what is shown in vivo (Fig. 6F), in which the MCT1 and CXADR expression seems mutually exclusive. Authors should co-immunostain at earlier developmental stages to test whether there is any overlapping expression.

6. One of the major conclusions of this manuscript is that Cxadr function in differentiating LPs promotes Gcm1 induction and SynT-II differentiation and suppresses SynT-I differentiation. This is further supported by the fact that SynT-I formation is excessively induced in Cxadr-KO TSCs. Thus, Cxadr expression in SynT-I/JZP progenitors should negatively regulates SynT-I formation and

promote JZP differentiation. it is surprising that there is no effect on JZP and TGC marker expression. Also, it is not clear how expression of Cxadr in JZP/SynT-I progenitor induces Gcm1 in SynT-II progenitors? Does Cxadr function inhibit proliferation of JZP/SynT-I progenitor thereby allowing relative expansion of Gcm1-expressing progenitors? What is the Gcm1 expression pattern in early time points (4-24h) of differentiation of Cxadr-KO cells, especially with inhibit condition or with CHIR?

7. Excessive SynT-I formation in Cxadr-KO TSCs is a different phenotype than what is observed in Cxadr-mutant mouse placenta, in which no significant alteration was observed for Syna expression and SynT-I formation. The authors need to perform scRNA-seq analyses with Cxadr-KO TSCs in stem and differentiating state (time course) to better understand the dynamics of the differentiation patterns of Cxadr-KO TSCs. Otherwise, the presented data with Cxadr-KO TSCs does not generate any definitive conclusion.

Minor Comments:

8. The introduction is too short and abruptly ends. Line 62 is confusing.

9. TGFb should be written as TGFβ.

10. The legend of X-axis of Fig. 3J is confusing.

11. There is no reference mentioned for E2F8 and PHF8 targets (line 268).

12. Synb expression (Fig. 7G) is not reduced (rather increased) in later time points in Cxadr-KO TSCs. Authors should clearly describe this.

13. The mentioning of Sox2-Flp mice in the discussion (583-585) is not necessary. Rather it raises question why the authors have not tried that approach to definitively conclude trophoblast-specific function of CXADR.

14. The relevance of this study, as presented, in the context of human placentation is rather thin. There is no clearly distinguishable SynT-I and SynT-II like populations in human placenta and CXADR is either not expressed or very lowly expressed in human trophoblast cells. Relevance to human placentation is very superficially mentioned in the abstract and at the end of the discussion. Authors should better extrapolate the relevance of their findings in the context of human placentation.

Reviewer #3 (Remarks to the Author):

This submission by Angelova et al. contains the seeds of a strong paper, but additional analyses and a change in focus are required. The first five figures and thirteen supplementary figures, centred around single-cell RNA-seq of differentiating TSCs, are generally competently conducted and useful but mostly a laundry list of genes and sequencing data. The authors identify new genes and regulons associated with stem and differentiated state but do not perform any genetic experiments to demonstrate their importance. Then around Line 384/Figure 6A, they make a very biologically important claim: that "JZP and SynT-1 precursors share similar differentiation trajectories". However, the evidence presented for this is lacking. In Figure 6A, a cluster of cells is circled and labeled "SynT-I prec". It is unclear how these cells are assigned (is it simply Slc16a1 expression?). Nothing is shown about the developmental trajectory that gives rise to these cells, and the evidence that they have a similar trajectory to JZP is fairly limited. They are next to JZP on the UMap and they are positive for Phlda2 (but so are SunT-II precursors). There may be any number of ways to show that JZP and SynT-I precursors share similar trajectories (for example, where do SynT-1 precursors appear in the trajectories in 4A and 4B?) but as the most important biological finding this requires more bioinformatic support, especially given the enormous amount of analysis earlier in the paper.

The subsequent observation that Cxadr loss promotes SynT-I and inhibits SynT-II differentiation is valuable and intriguing. Unless I am mistaken though, it seems to work against a model in which SynT-I and JZP precursors share similar trajectories, to the extent it implies some "branch point" where LTPs become either SynT-I or SynT-II, rather than SynT-I and JZP arising from the same branch.

Comments:

- Last sentence of abstract is unnecessary and out of place. This is an abstract for paper, not a grant application. Likewise, the first few paragraphs contain a lot more effusive language about the importance of studying pregnancy disorders and value of TSCs than is really necessary.

- Redraw figure 1 to show that both cell types form in both conditions, albeit with a modest bias toward LP in "Inhibit" and JZP in "remove

- Figures are called out of order (S2 before S1B, 4A-D after the entire rest of Figure 4). and sometimes wrong. Line 145 presumably refers to S4. Line 158 presumably refers to S4B.

- The batch names in Figure S1A (e.g. 00C) are not explained. Is there some sort of code that makes these letters and numbers make sense or are these the same as "batch 1, batch 2 etc."

- It is not supportable to claim that Nicol1 may play a role in the maintenance of the TSC state simply because it is expressed in TSCs, let alone that it "likely" does in the discussion. Likewise, discussion of genes and regulons specific to one lineage should not assume a biological role simply on the basis of enrichment in a lineage.

Reviewer #4 (Remarks to the Author):

In this manuscript, Angelova and Prater et al. perform extensive single cell RNA sequencing of differentiating Trophoblast stem cells (TSCs). Using two distinct conditions, either removal of Conditioned media (CM) and FGF or inhibition of MEK pathway (a downstream effector of FGF signaling) the authors aim to identify lineage driving factors of the JZP and LP lienages respectively. Additionally, they identify and validate Cxadr as a marker of LP cells involved in labyrinth cell maturation.

Some general remarks on the readability of this manuscript.

Please consider including a UMAP etc with the sample of origin (inhibit vs remove) and cell fate annotations labeled. This would greatly improve the readability of the manuscript as different lineages are difficult to follow just from cluster numbers. The text mentions the following cluster annotation: TSC cluster = c9, JZP cluster = c3, LP cluster = c4 but it would be helpful to show this and other fate annotations in a figure.

Plot titles, axis labels and figure legends/color scales are either missing or not legible in some of the figures (eg. Fig S10) making it hard to follow the text.

There are seemingly many instances of the wrong figure being referenced in the text? For instance Line 158 (L158) refers to fig S3 while it should be S4.

While the single cell data generated by the authors is quite unique and valuable, the analysis performed could be improved to better support the claims made in the paper.

It was unclear whether there were still any JZP lineage cells produced in the inhibit and LP lineage cells produced in the remove conditions. If so, I wonder if there are any differences between JZP cells obtained from the two conditions and if it impacts the identification of cell state drivers (same for LP). It would be helpful if the authors could comment on this/perform analysis to show presence or lack of such differences/ include this as a caveat in the text.

L146-150: Multiple claims have been made here about transcriptional differences/similarities based on qualitative assessment of distances between clusters on a UMAP. It has been widely

shown that distances on a 2D UMAP embedding do not always correspond to cell state differences. Some more rigorous ways to compare cell clusters would be - number of DE genes, comparing distances in a higher dimension space (e.g. PCA) or along a kNN graph.

L157: “Moreover, cells underwent this transition quicker in Inhibit conditions than in Remove conditions”. It is unclear what this claim is based on, some quantitative analysis supporting it would be greatly helpful. Same for this - L177-179: “In the Remove dataset, trophoblast differentiation markers showed similar temporal trends, but these were slightly delayed with TSC markers persisting longer into the time course”

L261-262: “The Remove dataset showed an increase in JZP markers Ascl2 and Plac1 starting earlier in pseudotime and being more pronounced than in the Inhibit dataset”. It will be helpful to include a plot making this comparison quantitatively (with statistical testing) in addition to the current pseudo times plots across main and supplementary figures.

For Figs 6e.f, it would be helpful if the authors could walk the readers through the observations. Perhaps, also including some quantitative image analysis of multiple fields of views in addition to the representative images (similar to fig 7f).

Point-by-point response to the reviewers' comments

Type of comment:	Comment:	Response:
	**Reviewer 1:**	
1. Remarks to author	In their manuscript titled 'Single Cell RNA Sequencing Identifies CXADR as a Determinant of Placental Exchange Surface Fate,' Angelova et al. employed a single-cell transcriptomics approach in combination with the time-course differentiation of mouse trophoblast stem cells (TSCs) using two distinct methods: Inhibition (inhibit MEK pathway) and Removal (CM and FGF4). The authors unveiled early trophoblast lineage diversification trajectories and identified multiple putative novel regulators. Specifically focusing on lineage specification towards junctional zone cell precursors (JZP) and labyrinth precursors (LP), the manuscript aimed to pinpoint molecular drivers. Notably, the authors functionally validated Cxadr as a key factor in balancing differentiation between the two syncytial layers of the mouse labyrinth.	
1A. Remarks to author: Additional validation	While the data presented in the manuscript holds promise for offering novel insights into cell fate determinants controlling trophoblast lineage specification, it appears that the manuscript was prepared hastily. There are so many errors, inadequate descriptions in the text and legends, and inconsistent labels in the figures, and all these make it challenging to comprehend the findings. Although the validation of Cxadr is carried out, its importance in trophoblast differentiation has been previously reported. Given the prediction of multiple novel regulators from the current study, performing functional validations on a few newly identified factors would enhance the robustness and significance of the research.	We apologize for the errors in figure referencing in our previous manuscript and have made the best of our efforts to remedy this in the current revision.<br><br>In our revision, we provide extensive additional functional validation of our data. For one, we follow up on *Nicol1*, a gene we had identified in our scRNA-seq data as a novel TSC gene. We demonstrate its expression in TSCs but acute down-regulation upon onset of differentiation (**new Fig. 2h, i**). Moreover, for this revision we also generated KO TSCs for *Nicol1*, a substantial and time-consuming undertaking. Our data demonstrate that *Nicol1* is indeed critical for stem cell

		maintenance (**new Fig. 2j, k** and **Suppl. Fig. 3**), as *Nicol1* KO TSCs exhibit greatly diminished expression levels of an entire repertoire of TSC markers. These additional insights elevate the significance of our data tremendously.<br><br>As to the novelty of our data around the functional relevance of CXADR, it is important to note that its role in trophoblast has NOT been previously reported. In fact it was argued that CXADR has no function in trophoblast (Outhwaite et a., 2019). Here, we demonstrate a key role of CXADR in regulating cell fusion rates towards the placental syncytiotrophoblast lineages, which is a key requirement for placenta formation and fetal survival. We bolster our insights by providing another entire set of scRNA-seq data on WT and KO cells for *Cxadr* (**new Fig. 8** and **Suppl. Fig. 17**). These data strongly corroborate the conclusions we had drawn from the marker-specific analysis and demonstrate that CXADR functions as a novel gatekeeper balancing SynT-I vs SynT-II differentiation.<br><br>Collectively, we believe that we have overhauled our manuscript substantially and provide many more fundamental experimental data and insights that have strengthened the conclusions tremendously.
2.. In-vivo validation	Are the expression profiles of LP and JZP cells in the current study similar to those observed in vivo? It is crucial to investigate whether they exhibit comparable expression patterns to bolster the significance of the study.	We performed important validation experiments for established LP and JZP marker genes by RT-qPCR (e.g., *Phda2*, *Gjb3*, *Atp11a*, *Gcm1*, *Synb*) and by immunostaining (STRA6, NCAM1) on our TSCs that were differentiated such that specific lineages are favoured. Of note, all of these markers are well-known for their trophoblast cell-type specific expression pattern in vivo. By using these markers, we confirmed that the described differentiation strategies indeed result in time-sensitive enrichments for JZP and LP populations in Remove and Inhibit conditions, respectively (**new Figs. 1c, 1d**), and that

		genes that cluster in the SynT-II branch of the UMAP are indeed SynT-II-enriched (**Suppl. Fig. 15b, c**).
3. Batches in dataset	Concerns regarding Figure 1 and Figure S1: In the main text, the authors did not clearly specify the nomenclature for individual time course samples and each batch. In Figure S1a, only seven datasets are presented; are these distinct batches, and what does the identity on the X-axis (00C, 0B0, 0BC, etc.) signify? The figure legends lack adequate information in several instances. Furthermore, in Figure 2a, only four batches are depicted, leaving three batches unaccounted for (identified in Figure S1).	We have improved the clarity of the sample description. We have added more information about replicate cultures and clarified that we sequenced and analysed the scRNA-seq data in batches of pooled libraries (**Supplementary table 25**). We had added more supplemental figures (with new data) so have replaced Fig. S1<br><br>In Fig. 2a only the 4 batches which contained t0 cells were included in this particular analysis.
4. Differences of primed TSCs to other TSCs	Regarding Line 111: The study predicts a small subset of cells already primed for differentiation, but ultimately, all cells appear to differentiate (Figure 3). If this holds true, what distinguishes primed TSCs from other TSCs, aside from minor expression differences? Are primed TSCs faster in differentiation?	To address this point, we chose to co-stain TSCs grown in stem cell conditions for SOX2, an acute stem cell marker, as well as for cytokeratin 18 (KRT18) which is strongly up-regulated in cluster 2 cells. Indeed, we found that small cell groups with a clearly more flattened, differentiated appearance have lost SOX2 but are strongly positive for KRT18, even in TSCs grown in stem cell conditions. This cell cluster can be visibly identified by morphological changes including a more flattened appearance and larger nuclei. This result corroborates the notion that cluster 2 identifies a small sub-population of cells in TSC cultures that are prone to differentiate. These data are shown in the **new Fig. 2g**.
5. Functional validation	Lines 112-124: To underscore the data's value in identifying critical TSC factors, such as Nicol1, additional validation may be essential. The main text highlights the identification of multiple novel regulators, emphasizing the necessity of functional validations for novel regulators associated with the JZP/LP lineages and SynT-II markers (line 383).	We now present additional data that demonstrate the strictly stem cell state-associated expression of *Nicol1* and its acute down-regulation upon onset of differentiation (new **Fig. 2h, i**).<br><br>Furthermore, we have generated *Nicol1* KO TSCs and show that these exhibit reduced expression levels of TSC marker genes (new **Fig. 2j, k** and **Suppl. Fig. 3**). This is an important and extensive additional validation that confirms the relevance of the scRNA-seq data in our current study.

		In addition, we provide important new experiments shown in the **new Figs. 1c, d** to validate the enrichment of JZP markers in Remove conditions, and conversely of LP markers in Inhibit conditions by RT-qPCR (**Fig. 1c**) and immunostaining (**Fig. 1d**). Validation has also been performed for SynT-II differentiation markers using *Abcb1a*, *Gabrp* and *Gjb2* by RT-qPCR and EMB by immunostaining (**Suppl. Fig. 15b, c**). In addition, we now also validate the JZP trajectory by assessing expression dynamics of *Tcf12*, *Tgif2*, *Tgif1* and *E2f4,* as well as of *Tpbpa* as control, upon rosiglitazone treatment, which is known to inhibit JZ differentiation (Parast *et al.*, 2009; PMID: 19956639) **(Suppl. Fig 12e).**<br><br>Collectively, these data strongly corroborate that the cells enter the expected differentiation trajectories.
6. Figure edits	Concerning Line 145 and Figure S3: The heatmap provided does not correspond to the Remove data. Throughout the figures, there are instances of mislabeled panels, missing legends, and inconsistencies in labels.	We apologise for the error, this should have been Suppl. Fig. 5a. The text has been corrected (new line 226).
7. Editorial comments	Other specific points of correction include Line 158 (no Figure S3b), Line 176 (Gjb3 is not present), Line 181 (clarification on the correct number of top marker genes: 10 or 5?), and Figure 3K (inconsistencies in legends regarding the 17 clusters).	Apologies, we should have referred to **Suppl. Fig. 5b**.<br>In Fig. 3j, Gjb3 is present, and our text (new line 277) correctly refers to this element<br>Line 181 – The top 10 markers genes were used for the heatmap shown in **Suppl. Fig. 6** and explicitly stated in this figure legend. Tables of all the marker genes are provided in **Supplementary Tables 7 and 8**.
8. Definition of regulon	Line 219 raises a query about the definition of "regulon," necessitating detailed explanations and references if applicable. Similarly, Line 266 requests information on the source of E2F8 targets, which is insufficiently provided in multiple instances.	We have added the following text and an additional citation to SCENIC: “ie modules of co-expressed genes and transcription factors with binding motif support” (new line 288).<br>E2F8 – we have added more detail and support for this (new line 315ff)
9. Missing description of figure	Supplementary Figure 8 lacks a description in the main text.	This has been corrected (new line 261).

10. Explanation for selecting Cxadr	Line 396 and Figure 6a: The process of selecting Cxadr as a candidate regulator is unclear, especially as the expression patterns of Cxadr and Mct1 differ (Figure 6a). A detailed explanation is necessary.	We clarified this point in the text: "Next, we wanted to pursue this observation that early SynT-I precursors share a similar differentiation trajectory to JZP. Given the fact that *Slc16a1* (*Mct1*) and *Hbegf* are widely used markers specific to SynT-I cells in the murine labyrinth (Nadeau and Charron, 2014; Radford et al., 2023) but clustered in the UMAPs in a cell group partially overlapping with JZP markers such as *Phlda2*, we asked whether other genes that shared a similar cluster enrichment as *Slc16a1* were also LP markers. Interrogating the Monocle modules to this effect identified the Coxsackie virus and adenovirus receptor (*Cxadr*) as one such candidate (**Fig. 6a** and **Supplementary Fig. 15d**)". The interpretation of the overlapping JZP/SynT-I trajectories is confirmed by our functional CXADR experiments that indeed corroborate this factor as a critical LP gene. Moreover, we have now performed an **entire new scRNA-seq experiment** on three independent WT and *Cxadr* KO clones each, and find a lineage-biased enrichment even already at the t24 (i.e. 24h differentiation in remove or Inhibit) time point. These data are shown in the **new Fig. 8** and **Suppl. Fig. 17**. We are sure the reviewer will appreciate the tremendous efforts that have gone into providing this new data set, which bolsters our conclusions. We hope that this extended justification, as well as the substantial amounts of additional data, have clarified the rationale for selecting *Cxadr* as a candidate gene to be investigated in more detail.
11. Figure legend	Figure 7e is missing its legend.	Apologies, the legend was present, but a paragraph mark was missing in the original manuscript. This formatting error has been corrected.
12. Cxadr expression	Concerns about Figure 7b and Figure S15a are raised. Cxadr expresses only detectable levels in TSCs, yet KO confirmation was performed in TSCs.	We apologize if this was unclear. *Cxadr* is expressed in TSCs, it is up-regulated during subsequent days of differentiation peaking at around 3D, and is then down-regulated in fusing syncytiotrophoblast cells. The KO confirmation was performed

		by PCR genotyping on genomic DNA, by RT-qPCR with primers inside the deleted exon in TSCs (**Suppl. Fig. 16a**) and in differentiating TSCs (**Suppl. Fig. 16b**), and by protein immunostaining where a clear absence of cell membrane staining can be appreciated (**Fig. 7b**). Please note that the heatmap in **Suppl. Fig. 15f** is scaled between min and max expression values to emphasize peak expression at 3D differentiation, it does not mean that *Cxadr* is not expressed in TSCs.
13. Cxadr expression levels testing	Lines 518-520 call for the testing of Cxadr expression levels, considering the enrichment of JZP differentiation in Remove conditions and favored LP differentiation in Inhibit conditions.	We have added additional experiments to further characterize *Cxadr* expression profiles under various differentiation conditions, which -together with the immunostaining data - corroborates the notion of CXADR as a labyrinth progenitor marker. We have added the following text and associated data: "To further characterize the expression dynamics of *Cxadr*, we profiled its expression levels across the 48h Remove-Inhibit time course. *Cxadr* was up-regulated as differentiation progressed, in particular in the Inhibit conditions that are LP-enriched (**Fig. 6e**). Extended TSC differentiation time course experiments in standard Remove conditions revealed that *Cxadr* peaked at 3 days of differentiation, preceding the onset of overt cellular syncytialization (**Supplementary Fig. 15f**). Upon enforced syncytialization which can be achieved by treating TSCs with the WNT activator CHIR, *Cxadr* expression declined (**Supplementary Fig. 15g**), in line with our observations that fusing cells down-regulate CXADR protein (**Fig. 6f**). These data corroborate the notion of *Cxadr* as a LP gene." (new lines 442-460).
14. Discussion of hTSC differentiation	The manuscript should incorporate a discussion on human trophoblast differentiation, particularly focusing on the multiple novel regulators predicted in the study, including CXADR.	We have amended the Discussion to include the role of CXADR in human trophoblast, including the importance of CXADR down-regulation in syncytiotrophoblast for preventing transplacental transmission of viral infections. We would like

		to thank the reviewer for prompting this important addition. (new lines 690 ff).

	**Reviewer #2**	
1.Remarks to author	This manuscript by Angelova and colleagues entitled "Single cell RNA sequencing identifies CXADR as a fate determinant of the placental exchange surface”" used single cell RNA sequencing of mouse TSCs in their stem state culture and under two differentiating conditions, remove condition (removal FGF and MEF conditioned medium) and inhibit condition (chemical inhibition of MEK) to capture gene expression changes during early stages of trophoblast differentiation during mouse placentation. In particular, authors used scRNA-seq to better understand temporal progression of TSC differentiation to Syncytiotrophoblast lineage to better understand development of SynT-I and SynT-II populations. The authors further focused on specific role of the Cxadr gene, which was earlier shown to be important for placental labyrinth development. The authors generated a Cxadr-KO mouse TSC line and found that the loss of CXADR promotes SynT differentiation but severely diminishes Gcm1 expression in differentiating TSCs, a finding which recapitulated earlier observation in Cxadr mutant mice. Thus, the authors concluded that cell-autonomous function of Cxadr gene is a gatekeeper to balance SynT-I vs. SynT-II differentiation.<br><br>The study is interesting and provides novel information a better picture of gene expression dynamics during early stages of mouse trophoblast differentiation using mouse TSC as a model system. Thus, the study could be informative to the field.	

1A. Additional experimental approaches	However, enthusiasm is diminished due to breadth of approaches and conclusions are almost entire reliant on scRNA-seq data and associated RT-PCR data. Additional experimental approaches are necessary to make definitive conclusions about the claims made for phenotypic variations of the stem-state and differentiating cells.	In the revised version of our manuscript, we have added substantial amounts of additional experimental data to further strengthen our conclusions (**new Figs. 1b-d, 2g-k, 6b, 6d-e, 8**; **Suppl. Figs. 1, 3, 12f 15g, 16d** & **17**) including KO of the novel stem cell marker *Nicol1* and single cell sequencing of *Cxadr* KO cells. It should also be pointed out that our data on CXADR are entirely novel insofar as they point to a critical role of this protein in balancing the amounts of cells that enter the two syncytiotrophoblast layer lineages (i.e. it balances SynT-I vs SynT-II differentiation). These insights fundamentally extend previous data that concluded a role for CXADR solely in endothelial cells. Here, we show that CXADR is a critical regulator of the cell fusion dynamics that are essential for normal placental labyrinth formation.
1B. Data interpretation	The conclusion about the role of Cxadr is confusing based on expression during TSC differentiation and phenotype of Cxadr-KO TSCs. Different experimental conditions for SynT differentiation for different experiments have also complicated the interpretation of the data.	We apologize for any confusion caused. Our data show that *Cxadr* is an important regulator of trophoblast cell fusion. Thus, we show that the loss of CXADR membrane localization is prerequisite for syncytialization. In the absence of CXADR, trophoblast cells fuse excessively, and predominantly to SynT-I. Our various differentiation experiments were aimed to identify the fine-tuning role of CXADR in SynT-I and SynT-II differentiation, the latter being promoted by CHIR. We have clarified the rationale of these experiments in the text.
1C. Value of new data on Cxadr	Given the already characterized placental phenotype of Cxadr-KO mouse model, the new data related to CXADR seems incremental. More analyses are necessary. My concerns are described below and should be addressed.	As outlined in response 1A above, our data describing a critical role for CXADR in trophoblast are entirely novel. The addition of extensive scRNA-seq data on *Cxadr* KO TSCs in this revision further deepens these novelty aspects. In a nutshell, <u>previous studies</u> concluded that CXADR has an <u>exclusive function in the embryo</u> to affect placental labyrinth formation. In stark contrast, we identify an essential, cell autonomous function of CXADR in trophoblast cells that relates to guiding the balanced, finely-tuned differentiation dynamics of labyrinth progenitors into SynT-I and SynT-II cells. These insights substantially

		advance our understanding of the cell type-specific functions of CXADR.
2. Changes in morphology in differentiating cells	1. The entire characterization of cell types in stem and differentiating stages rely on data from scRNA-seq analyses and a fold change of ≥1.5 or ≥1.2. This is concerning and needs more validation. For example, as mentioned in the manuscript, it is well known that mouse TSC cultures have heterogeneous cell morphology and contains cells in true stem-state vs. differentiating state. However, the level of morphological heterogeneity varies from culture to culture and duration of culture. Thus, authors should show the images of TSC colonies that are representative of cultures that they have used for their scRNA-seq analyses in stem state as well as differentiating conditions (Time course).	As suggested by the reviewer, we now include representative photographs of the cells at every time point and condition in the **new Fig. 1b**. We also performed substantial amounts of validation experiments that confirm the expected differentiation trajectories in the Remove and Inhibit conditions (**new Figs. 1c-d, 6c-d, Suppl. Fig. 12f**) as well as the identification of TSCs at the cusp of differentiation onset in stem cell conditions (**new Fig. 2g**).
3. Gene expression in t0 clusters	The differentiating cells in stem state condition (t0 clusters 1 and 2 in Fig. 2a) needs better characterization for their differentiation trajectory. Does these cells have some induction of Hand1, Gcm1 and Cxadr? Characterization of *Nicol1* as stem cell gene. Authors should also test whether MEK activation reduces number cells of t0 clusters 1 and 2. Also, authors should also test that prolonged MEK inhibition is not inducing apoptosis markers.	We have added multiple experiments to address this point: Double-staining for SOX2, an acutely sensitive TSC state transcription factor (Adachi et al. 2013, Latos et al., 2015) and for KRT18, a gene we identified to be highly up-regulated in c2-cells, demarcates small cell colonies that are prone to differentiate in standard TSC cultures (**new Fig. 2g**). The c2-induced genes are displayed in **Fig. 2c** and they do include *Hand1*, but it is not possible to sub-divide this small cell cluster into additional groups that would indicate downstream trajectories. As such, in stem cell conditions, these cells are best characterized as having lost their acute stemness state (i.e. SOX2 expression).<br>We have now characterized the expression dynamics of *Nicol1* and verify it as a highly sensitive marker of the TSC state that is abruptly down-regulated with the onset of differentiation (**new Fig. 2h, i**). For this revision, we also generated *Nicol1* KO TSCs and demonstrate the importance of this gene for TSC maintenance (**new Fig. 2j-k, Suppl. Fig. 3**).

		Finally, as suggested we have assessed cell viability rates upon MEK inhibitor treatment and found no detrimental effects during the time frames that this inhibitor was applied (**new Suppl. Fig. 1**).
3A. Validation of mTSC markers	Additional experimental approaches (IHC, Immunofluorescence) are needed to show the markers that authors claim to be specific to true TSCs (such as Nicol1). This is important to show whether expression levels are varying in cells with different morphology.	We have now determined the dynamics of *Nicol1* expression during differentiation by RT-qPCR (**new Fig. 2h**) and of NICOL1 protein by immunostaining (**new Fig. 2i**) and show that this factor is rapidly down-regulated upon TSC differentiation. Moreover, we demonstrate in newly generated KO TSC lines that *Nicol1* is essential for the maintenance of the TSC state (**new Figs. 2j, k Suppl. Fig. 3**).<br>These additional experiments constitute substantial efforts that have gone into this revision and that have strengthened the impact of our study tremendously.
3B. MEK inhibition	Authors should also test whether MEK activation reduces number cells of t0 clusters 1 and 2. Also, authors should also test that prolonged MEK inhibition is not inducing apoptosis markers.	We have tested cell viability upon MEK inhibitor treatment for the duration used in our time course experiments and find no evidence of increased apoptosis rates, i.e. cell viability remains unchanged (**new Suppl. Fig. 1**).
4. Experimental validation of gene expression in differentiation	3. As noted, mouse TSC differentiation system (especially the remove system) is not a very robust model for SynT differentiation due to transient and non-synchronous differentiation. Thus, data presented in Figs. 3, 4 and 5 and in Supplementary Figs. 5, 6 and 7 need to be supported by cell culture images (inhibit vs. remove conditions), and additional experimental approaches (IHC or IF) for marker expressions (such as E2F8, PHF8, Plac1, Klf8, B2m, Phlda2 etc). This will provide a better visual understanding of cell populations that were undergoing differentiation process. Does the inhibit condition clearly show SynT formation?	As requested, we have added substantial amounts of additional data to better characterize the Remove and Inhibit differentiation conditions. We tested various markers by RT-qPCR and depict representative JZ and Labyrinth genes to corroborate the preferential differentiation trajectories in Remove and Inhibit conditions (**new Fig. 1c**). We have also tested a multitude of antibodies against trophoblast cell type-specific markers and now include proof of JZ- and SynT-enriched differentiation in Remove and Inhibit, respectively, in the **new Fig. 1d**, and of non-overlap of precursors in the **new Fig. 6b** and **6d**. Furthermore, we have characterized the t0 cluster 2 cells more carefully and indeed find a striking mutual

		exclusiveness between expression of the TSC marker SOX2 and the cluster 2-enriched factor KRT18 (**new Fig. 2g**).
5. SynT formation images	4. It is necessary to better characterize SynT-II only differentiation with CHIR. Cell colony images with MCT1 and MCT4 expression and SynT formation should be shown.	CHIR has been published previously to specifically induce SynT-II differentiation (PMID: 29153986), and this has been corroborated in our hands (PMID: 36859534), including in the current study in **Suppl. Fig. 15b**.
5A. Emb gene expression in cell culture model	The introduction of Emb gene is sudden (lines 378-379) in the manuscript and is not clear whether a similar expression pattern (overlapping with MCT4) was observed in the cell culture model.	Embigin (Emb) was used as an additional gene to prove the SynT-II identity of cluster 4-cells. In addition to the RT-qPCRs on CHIR-treated TSCs above, we identified a well-working antibody against EMB to validate SynT-II specificity directly on placentas. The perfect overlap with MCT4 verifies this point, which is also in line with Emb having been identified as SynT-II gene in a recent scRNA-seq study (Marsh & Blelloch 2020). This collective evidence provides further confirmation of the SynT-II-directed differentiation of TSCs upon CHIR treatment. We have added additional text to explain this more clearly, including in the Discussion (New lines 410-419 and 601-606).
6. Cxadr and Mct1 expression	5. The induction of Cxadr in Mct1-expressing cluster (Fig. 6a, Supplementary Fig. 14D) is different from what is shown in vivo (Fig. 6F), in which the MCT1 and CXADR expression seems mutually exclusive. Authors should co-immunostain at earlier developmental stages to test whether there is any overlapping expression.	We apologize for this confusion. In the UMAPs (Fig. 6a), the SynT-I precursor enrichment can be clearly made out by well-established SynT-I genes such as *Mct1* (*Slc16a1*) and *Hbegf*. *Cxadr* broadly falls into the same cluster of cells. Yet on the protein level, it is clear that CXADR is down-regulated as cells actually start to fuse, whereas MCT1 staining only becomes evident in mature (i.e. fusing or fused) SynT-I cells. Thus, the discrepancy can be explained by the detection of LP as well as mature SynT-I markers at the mRNA level at early time points of differentiation, whereas their protein dynamics temporally diverge as SynT-I cells mature. This temporal relationship between progenitors and maturing cells can also be seen in the UMAPs in Remove conditions in which differentiation into SynT cells occurs more slowly **(Suppl. Fig. 15d**). Here, *Cxadr*-enriched cells fall just at the margins of the *Hbegf*- and

		Slc16a1-enriched clusters, again corroborating *Cxadr* as an LP gene.
7. Mechanism of Cxadr effect on SynT-II	6. One of the major conclusions of this manuscript is that Cxadr function in differentiating LPs promotes Gcm1 induction and SynT-II differentiation and suppresses SynT-I differentiation. This is further supported by the fact that SynT-I formation is excessively induced in Cxadr-KO TSCs. Thus, Cxadr expression in SynT-I/JZP progenitors should negatively regulates SynT-I formation and promote JZP differentiation. it is surprising that there is no effect on JZP and TGC marker expression. Also, it is not clear how expression of Cxadr in JZP/SynT-I progenitor induces Gcm1 in SynT-II progenitors? Does Cxadr function inhibit proliferation of JZP/SynT-I progenitor thereby allowing relative expansion of Gcm1-expressing progenitors? What is the Gcm1 expression pattern in early time points (4-24h) of differentiation of Cxadr-KO cells, especially with inhibit condition or with CHIR?	We have addressed this point both bioinformatically and by immunostaining to distinguish between the two possible scenarios that (i) cells are bipotential or (ii) precursors cluster in similar regions of the UMAPs but remain distinct cells. Reassuringly, both strategies resulted in the same conclusion and demonstrate the mutual non-exclusiveness between JZP and SynT-I LP cells. This is shown in the **new Figs. 6b, 8g** and **Suppl. Fig. 17g** by highlighting JZP and SynT-I marker expressing single cells that show no evidence for bipotentiality once marker gene expression levels start to rise. Moreover, in the **new Fig. 6d**, we provide immunostaining proof of the mutual non-overlap between JZP and SynT-I marker-positive cells. This has now also been highlighted in the text: "While SynT-I progenitors appear in a similar trajectory to JZP in the UMAP plots, it is important to note that each individual cell retains a distinct fate (**Fig. 6d**) as opposed to exhibiting bipotential characteristics." Thus, although these cell populations follow similar differentiation trajectories on the UMAP, they are not the same cell (new lines 423-431).<br>These data imply that CXADR does not directly up-regulate *Gcm1*, but rather that it acts to suppress cell fusion, and specifically SynT-I maturation. Such a function is well in line with the cell membrane localization of CXADR that is enriched in tight junctions.<br><br>Finally, we have also addressed the reviewer's request of assessing *Gcm1* expression at early time points in WT and *Cxadr* KO TSCs (**new Suppl. Fig. 16d**). In general, it should be noted that SynT-II formation precedes SynT-I formation *in vitro* by 1-2 days. We find that at 24h, *Gcm1* expression is elevated

		in the *Cxadr* KO cells, which is more pronounced in the Remove and CHIR conditions that specifically push TSCs towards JZ and SynT-II cells, respectively. Thus, although the main impact of *Cxadr* deletion is on increasing SynT-I formation, it does regulate the cell fusion dynamics in general. We have now amended the text accordingly to highlight this point and would like to thank the reviewer for requesting this important experiment.
8. scRNAseq of Cxadr KO cells	7. Excessive SynT-I formation in Cxadr-KO TSCs is a different phenotype than what is observed in Cxadr-mutant mouse placenta, in which no significant alteration was observed for Syna expression and SynT-I formation. The authors need to perform scRNA-seq analyses with Cxadr-KO TSCs in stem and differentiating state (time course) to better understand the dynamics of the differentiation patterns of Cxadr-KO TSCs. Otherwise, the presented data with Cxadr-KO TSCs does not generate any definitive conclusion.	Firstly, of note, there is evidence for increased amounts of SynT-I in *Cxadr* KO placentas (Outhwaite et al., 2019, Fig. 3j), even if this has not been explicitly stated in that paper. More importantly, however, we have now carried another large round of scRNA-sequencing using three independently derived WT and *Cxadr* KO cell lines each, as requested. We have included cells in stem cell conditions as well as after 24h of differentiation in Remove and Inhibit conditions. This has been a substantial amount of work. These data are presented in the **new Figs. 8** and **Suppl. Fig. 17**. Reassuringly, the data strongly corroborate our conclusions around the critical role of CXADR in toggling SynT-I vs SynT-II differentiation, which bolsters our manuscript tremendously.
9. Introduction length	8. The introduction is too short and abruptly ends. Line 62 is confusing.	We have revised the Introduction, now ending with a brief summary of key findings, and have also rephrased the highlighted sentence (new line 111).
10. Nomenclature	9. TGFb should be written as TGFβ.	We have amended the spelling accordingly.
11. Figure legend	10. The legend of X-axis of Fig. 3J is confusing.	We had added additional detail in the figure legend to clarify this.
12. Reference for TF targets	11. There is no reference mentioned for E2F8 and PHF8 targets (line 268).	SCENIC uses the presence of transcription factor response elements and transcript correlations to identify candidate targets.

13. Description of Synb expression	12. Synb expression (Fig. 7G) is not reduced (rather increased) in later time points in Cxadr-KO TSCs. Authors should clearly describe this.	*Synb* expression at >48h is not significantly altered between WT and *Cxadr* KO TSCs (Fig. 7g), our results highlight the pertinent, significant changes that occur upon *Cxadr* deletion.
14. Topic of Sox2-flp mice in discussion	13. The mentioning of Sox2-Flp mice in the discussion (583-585) is not necessary. Rather it raises question why the authors have not tried that approach to definitively conclude trophoblast-specific function of CXADR.	The discussion around the previous *Sox2*-Cre mediated embryo-specific deletion of *Cxadr*, and the recently developed *Sox2*-Flp tool to generate trophoblast-specific conditional KOs, is highly pertinent to our work because we identify a trophoblast-specific function of CXADR in the current manuscript. This substantially extends previous data that suggested an embryonic lineage-exclusive role of this membrane protein.
15. Context of study in human placentation in discussion	14. The relevance of this study, as presented, in the context of human placentation is rather thin. There is no clearly distinguishable SynT-I and SynT-II like populations in human placenta and CXADR is either not expressed or very lowly expressed in human trophoblast cells. Relevance to human placentation is very superficially mentioned in the abstract and at the end of the discussion. Authors should better extrapolate the relevance of their findings in the context of human placentation.	We have amended the Discussion and added pertinent information as to the role of CXADR in human development and in the human placenta. Indeed, CXADR has been detected in human trophoblast cells, but - just like in the mouse - CXADR is absent from syncytiotrophoblast cells (Koi et al., 2001). From our data, it is likely that CXADR regulates cell fusion in the human placenta as well. Moreover, previous studies have highlighted that down-regulation in the maternal blood-exposed syncytiotrophoblast layer may act as a protective mechanism against transplacental transmission of viruses. Moreover, CXADR expression in EVTs makes them susceptible to virus-induced apoptosis, rendering the affected pregnancies more susceptible to serious complications such as preeclampsia and miscarriage (Koi et al., 2001).

	**Reviewer #3:**	

1.Remarks to the author	This submission by Angelova et al. contains the seeds of a strong paper, but additional analyses and a change in focus are required. The first five figures and thirteen supplementary figures, centred around single-cell RNA-seq of differentiating TSCs, are generally competently conducted and useful but mostly a laundry list of genes and sequencing data. The authors identify new genes and regulons associated with stem and differentiated state but do not perform any genetic experiments to demonstrate their importance. Then around Line 384/Figure 6A, they make a very biologically important claim: that "JZP and SynT-1 precursors share similar differentiation trajectories". However, the evidence presented for this is lacking. In Figure 6A, a cluster of cells is circled and labeled "SynT-I prec". It is unclear how these cells are assigned (is it simply Slc16a1 expression?). Nothing is shown about the developmental trajectory that gives rise to these cells, and the evidence that they have a similar trajectory to JZP is fairly limited. They are next to JZP on the UMap and they are positive for Phlda2 (but so are SunT-II precursors). There may be any number of ways to show that JZP and SynT-I precursors share similar trajectories (for example, where do SynT-1 precursors appear in the trajectories in 4A and 4B?) but as the most important biological finding this requires more bioinformatic support, especially given the enormous amount of analysis earlier in the paper.<br><br>The subsequent observation that Cxadr loss promotes SynT-I and inhibits SynT-II differentiation is valuable and intriguing. Unless I am mistaken though, it seems to work against a model in which SynT-I and JZP precursors share similar trajectories, to the extent it implies some "branch point" where LTPs become either SynT-I or SynT-II, rather than SynT-I and JZP arising from	Firstly, we would like to thank the reviewer for the supportive assessment.<br><br>In this revision, we have made substantial efforts to further substantiate the observation around the early shared JZP/SynT-I trajectory of cells. In general, cell clusters were assigned lineage identity on the basis of multiple markers, as shown. In all of our scRNA-seq data, i.e. the original Inhibit and Remove datasets as well as in the newly performed scRNA-seq of WT and *Cxadr* KO TSCs, SynT-I progenitors always cluster more closely with JZPs as compared to SynT-II progenitors. Thus, pseudotime trajectories drawn by Monocle show that the trajectories for SynT-I and JZP haven't clearly separated yet while SynT-II cells are clearly separate. This is evident in **Figs. 6a, Suppl. Fig. 15d,** and **the new Figs. 8a** and **Suppl. Figs. 17a**. We have spent considerable efforts to further elaborate on the point of differentiation trajectories and branch points. Thus, it should be noted that shared UMAP localizations are not implying that individual cells are bipotential, they just broadly share a larger fraction of their gene expression profiles. We have now added additional data (**new Fig. 6b, 6d, 8g, Suppl. Fig. 17g**) to demonstrate that JZP and SynT-I markers are not co-expressed on the same cell. To further clarify this point, we depict below a differentiation diagram that is consistent with our interpretation, which we hope will help to clarify the reviewer's point.

	the same branch.	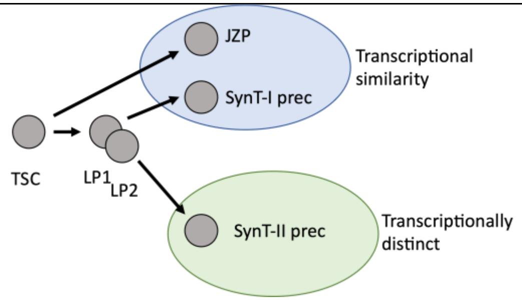
2. Abstract and introduction contents	- Last sentence of abstract is unnecessary and out of place. This is an abstract for paper, not a grant application. Likewise, the first few paragraphs contain a lot more effusive language about the importance of studying pregnancy disorders and value of TSCs than is really necessary.	We feel that it is important for the broader readership to highlight the translational importance of studying placental development and the molecular regulation of the early trophoblast differentiation steps that are of particular importance for pregnancy success. Indeed, other reviewers requested to include a broader discussion of the relevance of the data for human pregnancy conditions, and hence we have amended the manuscript accordingly.
3. Figure 1 visuals	- Redraw figure 1 to show that both cell types form in both conditions, albeit with a modest bias toward LP in "Inhibit" and JZP in "remove	We have taken this point on board and have redrawn Fig. 1a accordingly, thank you for the suggestion.
4. Figure references in text	- Figures are called out of order (S2 before S1B, 4A-D after the entire rest of Figure 4). and sometimes wrong. Line 145 presumably refers to S4. Line 158 presumably refers to S4B.	We have corrected these errors.
5. Batch IDs	- The batch names in Figure S1A (e.g. 00C) are not explained. Is there some sort of code that makes these letters and numbers make sense or are these the same as "batch 1, batch 2 etc."	This nomenclature referred to analysis batches. We have clarified this in the text and replaced Fig. S1.
6. Wording of claims	- It is not supportable to claim that Nicol1 may play a role in the maintenance of the TSC state simply because it is expressed in TSCs, let alone that it "likely" does in the discussion. Likewise, discussion of genes and regulons specific to one lineage should not assume a biological role simply on the basis of enrichment in a lineage.	We have added a substantial amount of additional data, including the generation and analysis of KO TSCs for *Nicol1*. This functional validation confirms the essential role of *Nicol1* as a TSC gene that is critical for maintaining the stem cell state. Thus, we have determined the dynamics of *Nicol1* expression during differentiation by RT-qPCR (**new Fig. 2h**) and of NICOL1

		protein by immunostaining (**new Fig. 2i**) and show that this factor is rapidly down-regulated upon TSC differentiation. Moreover, we demonstrate in newly generated KO TSC lines that *Nicol1* is essential for the maintenance of the TSC state (**new Figs. 2j, k** and **Suppl. Fig. 3**); in the absence of *Nicol1*, TSCs exhibit greatly diminished expression of the repertoire of TSC marker genes.<br>These additional experiments constitute substantial efforts that have strengthened the impact of our study tremendously. However, we agree with the reviewer's comment that enrichment in regulons does not implicitly equate biological function, and we have dampened the language in other parts of the manuscript where we highlight regulon-enriched factors.

	**Reviewer #4**	
1.Remarks to the Author	In this manuscript, Angelova and Prater et al. perform extensive single cell RNA sequencing of differentiating Trophoblast stem cells (TSCs). Using two distinct conditions, either removal of Conditioned media (CM) and FGF or inhibition of MEK pathway (a downstream effector of FGF signaling) the authors aim to identify lineage driving factors of the JZP and LP lienages respectively. Additionally, they identify and validate Cxadr as a marker of LP cells involved in labyrinth cell maturation.	
2. UMAP labelling	Please consider including a UMAP etc with the sample of origin (inhibit vs remove) and cell fate annotations labeled. This would greatly improve the readability of the manuscript as different lineages are difficult to follow just from cluster numbers. The text mentions the following cluster annotation: TSC cluster = c9, JZP cluster = c3, LP cluster = c4 but it would be helpful to show this and other fate annotations in a figure.	We agree with the reviewer that including cell fate annotation would aid the readability. In single cell analyses in tissue this is much easier to do (for example distinguishing endothelial cells from epithelial cells.). However, we are analysing a single cell type on a differentiation trajectory. Some of the clusters are recognisable but for many, classification is less clear-cut. We feel it would be potentially misleading if we assigned definitive labels to the clusters where we are uncertain. Nonetheless we

		agree some labels would be helpful, and hence we have added these where we are sufficiently confident of the assignment.
3. Figure formatting	Plot titles, axis labels and figure legends/color scales are either missing or not legible in some of the figures (eg. Fig S10) making it hard to follow the text.	This has been corrected.
4. Figure referencing in text	There are seemingly many instances of the wrong figure being referenced in the text? For instance Line 158 (L158) refers to fig S3 while it should be S4.	This has been corrected.
5. Analysis of single cell data	While the single cell data generated by the authors is quite unique and valuable, the analysis performed could be improved to better support the claims made in the paper.	Our study is entirely novel insofar as it investigates cell fate trajectories of early trophoblast lineage entry points, and we would like to thank the reviewer for sharing this supportive view around the considerable value of our data. We have now added substantial additional analyses as well as an entirely new scRNA-seq dataset of WT and *Cxadr* KO TSCs. We have improved the bioinformatic analysis of the shared JZP and SynT-I trajectories (**new Figs. 6b, 6d, 8, Suppl. Fig. 17**). Moreover, we have now also added a substantial amount of additional functional data, including the generation of a new TSC KO for *Nicol1* (**new Fig. 2g-k**) as well as a more finely grained characterization of our differentiation strategies (**new Fig. 1b-d**). Collectively, these efforts have strengthened the conclusions of our manuscript tremendously.
6. Differences between cell populations produces with the two differentiation conditions	It was unclear whether there were still any JZP lineage cells produced in the inhibit and LP lineage cells produced in the remove conditions. If so, I wonder if there are any differences between JZP cells obtained from the two conditions and if it impacts the identification of cell state drivers (same for LP). It would be helpful if the authors could comment on this/perform analysis to show presence or lack of such differences/ include this as a caveat in the text.	Our scRNA-seq data as well as our functional data on the cells demonstrate that LP and JZP are produced in both, Remove and Inhibit conditions.<br>We have now performed additional experiments to verify the enrichment of JZPs in Remove and of LPs in Inhibit conditions, respectively, at early time points (**new Fig. 1c, d**). Overall, Inhibit conditions strongly accelerates differentiation in general, whereas differentiation progresses more slowly in the conventional Remove condition (see Fig. 5a for example). To support this observation, we show in **Suppl. Fig. 5b** that in the Inhibit conditions the proportion of cells scored to phase G2M

		is significantly lower from 24h to 48h than the Remove condition. Since the speed of differentiation differs between the two differentiation strategies, we cannot directly compare differentiation “outcomes” between them in our scRNA-seq data (because the cells are generally still at intermediate stages of differentiation and not fully mature yet).
7. Description of clusters on UMAP	L146-150: Multiple claims have been made here about transcriptional differences/similarities based on qualitative assessment of distances between clusters on a UMAP. It has been widely shown that distances on a 2D UMAP embedding do not always correspond to cell state differences. Some more rigorous ways to compare cell clusters would be - number of DE genes, comparing distances in a higher dimension space (e.g. PCA) or along a kNN graph.	We have removed the misleading wording from this paragraph.
8. Temporal trends in differentiation	L157: “Moreover, cells underwent this transition quicker in Inhibit conditions than in Remove conditions”. It is unclear what this claim is based on, some quantitative analysis supporting it would be greatly helpful. Same for this - L177-179: “In the Remove dataset, trophoblast differentiation markers showed similar temporal trends, but these were slightly delayed with TSC markers persisting longer into the time course”	We have added a new figure plotting the fraction of cells scored as being in the G2M phase of the cycle and how this changes over time (new **Suppl. Fig. 5b**). This clearly shows that more of the Inhibit cells have exited the cycle at 24 hours than the Remove cells.
9.Expression of markers in pseudotime	L261-262: “The Remove dataset showed an increase in JZP markers Ascl2 and Plac1 starting earlier in pseudotime and being more pronounced than in the Inhibit dataset”. It will be helpful to include a plot making this comparison quantitatively (with statistical testing) in addition to the current pseudo times plots across main and supplementary figures.	We have softened this statement but as we have already added many additional figures we have not included the suggested plot (new line 311).
10. Description of figures	For Figs 6e.f, it would be helpful if the authors could walk the readers through the observations. Perhaps, also including some quantitative image analysis of multiple fields of views in addition to the representative images (similar to fig 7f).	Fig. 6f (previous 6e) shows a qualitative image of a TSC colony in which the innermost cells are starting to fuse. The onset of fusion is evident by the loss of continuous membrane labelling with the membrane marker ZO1, that instead becomes discontinuous and punctate. The corresponding cells have

		already entirely lost their membrane-localized CXADR staining. This pattern is consistently observed and correlates with the staining behaviour of CXADR in vivo (**Fig. 6g**) as well as with previously reported findings in blastocyst-stage embryos (Krivega et al., 2014; https://doi.org/10.1530/REP-14-0253). The qualitative images of placental staining cannot easily be quantified due to the complexity of the signals, and this would not add additional information to what is shown.

REVIEWERS' COMMENTS

Reviewer #1 (Remarks to the Author):

This revised version of the manuscript by Angelova et al. thoroughly addresses the previous concerns raised by the reviewer by correcting errors, clarifying descriptions, and adding new data. I have no further comments and support the publication of this revised manuscript.

Reviewer #3 (Remarks to the Author):

Most of my concerns have been addressed well, the new figures on Nicol1 (Fig.2 h-k) enhances the paper substantially, and the point re: JZP vs. SynT-1 trajectory has been clarified, albeit with the slightly less exciting result that JZP and SynT-1 cells share a lot of the same transcriptional program rather than genuinely emerging from a bipotent cells.

My only remaining concern is that it would be helpful to have adjacent figures for the “Remove” condition, showing pseudotime and JZP, SynT-1 and SynT-II. It is a struggle to keep track of markers and go back and forth through supplementary figures to try to figure out the path toward JZP, SynT-1 and SynT-II in these cells.

Minor comments:

-Figure S1 caption: “RFU” not explained.

-Figure S3d: Clarify what replicates are (different cell lines?).

-Line 227 – 232: “As TSCs differentiate, the fraction of cells in the S and G1 phases of the cell cycle diminishes”. Should this read “S and G2M:? Certainly the differentiated cells are dominantly G1.

-Figure 7g: clearer if indicated as “d3” rather than “3D”, given that the latter is how “3-dimensional” is typically rendered.

-Somewhere in Figure 8 or Figure S17, please just label the WT and Cxadr KO cells in the single cell RNA-seq UMAP.

-Line 579: This is one of several places where cells that show some differentiation markers are called “prone to differentiation”. This makes it sound like they are undifferentiated but are especially susceptible to differentiation, but this isn’t right. First, they are already differentiating, second we have no evidence that these are more capable of differentiation upon addition of MEKi

for example than other cells in the population. Isn’t it more accurate to say that they have undergone differentiation?

Reviewer #4 (Remarks to the Author):

The authors have sufficiently addressed my comments

	Comment	Our response
	Comment	Our response
1	Most of my concerns have been addressed well, the new figures on Nicol1 (Fig.2 h-k) enhances the paper substantially, and the point re: JZP vs. SynT-1 trajectory has been clarified, albeit with the slightly less exciting result that JZP and SynT-1 cells share a lot of the same transcriptional program rather than genuinely emerging from a bipotent cells.	
2	My only remaining concern is that it would be helpful to have adjacent figures for the “Remove” condition, showing pseudotime and JZP, SynT-1 and SynT-II. It is a struggle to keep track of markers and go back and forth through supplementary figures to try to figure out the path toward JZP, SynT-1 and SynT-II in these cells.	We have created a new figure 4 representing the pseudotime trajectories for Inhibit and Remove in the same figure.
3	Figure S1 caption: “RFU” not explained.	RFU is now defined as Relative Fluorescence Units
4	Figure S3d: Clarify what replicates are (different cell lines?).	We now have clarified in the legend that the data shown is of n=6 WT and n=4 KO independently derived trophoblast stem cell clones.
5	Line 227 – 232: “As TSCs differentiate, the fraction of cells in the S and G1 phases of the cell cycle diminishes”. Should this read “S and G2M:? Certainly the differentiated cells are dominantly G1.	Thank you, we have corrected this sentence (line 193).
6	Figure 7g: clearer if indicated as “d3” rather than “3D”, given that the latter is how “3-dimensional” is typically rendered.	We don’t use any 3D culture methods in this study and the figure is correct and clearly defined. Therefore we have not changed this labelling.
7	Somewhere in Figure 8 or Figure S17, please just label the WT and Cxadr KO cells in the single cell RNA-seq UMAP.	We have added this information in the new Supplementary Figure 17.
8	Line 579: This is one of several places where cells that show some differentiation markers are called “prone to differentiation”. This makes it sound like they are undifferentiated but are especially susceptible to differentiation, but this isn’t right. First, they are already differentiating, second we have no evidence that these are more capable of differentiation upon addition of MEKi for example than other cells in the population. Isn’t it more	We have rephrased the sentence to state “a small subset of cells that has already started to differentiate” (line 440).

	accurate to say that they have undergone differentiation?	
